# Isolation and In Silico SARS-CoV-2 Main Protease Inhibition Potential of Jusan Coumarin, a New Dicoumarin from *Artemisia glauca*

**DOI:** 10.3390/molecules27072281

**Published:** 2022-03-31

**Authors:** Yerlan M. Suleimen, Rani A. Jose, Raigul N. Suleimen, Margarita Y. Ishmuratova, Suzanne Toppet, Wim Dehaen, Aisha A. Alsfouk, Eslam B. Elkaeed, Ibrahim H. Eissa, Ahmed M. Metwaly

**Affiliations:** 1 The International Centre for Interdisciplinary Solutions on Antibiotics and Secondary Metabolites, Republican Collection of Microorganisms, Nur-Sultan 010000, Kazakhstan; syerlan75@yandex.kz; 2 The Laboratory of Engineering Profile of NMR Spectroscopy, Sh. Ualikhanov Kokshetau University, Kokshetau 020000, Kazakhstan; 3 Molecular Design & Synthesis, Department of Chemistry, Catholic University of Leuven, B-3001 Leuven, Belgium; alphmanie@gmail.com (R.A.J.); tsuzanne@kuleuven.be (S.T.); wim.dehaen@kuleuven.be (W.D.); 4 Department of Chemistry, St. Dominic’s College, Mahatma Gandhi University, Kanjirappally 686512, India; 5 Department of Technical Physics, Faculty of Physics and Technology, L.N. Gumilyov Eurasian National University, Nur-Sultan 010010, Kazakhstan; 6 Department of Botany, E.A. Buketov Karaganda University, Karaganda 100024, Kazakhstan; margarita.ishmur@mail.ru; 7 Department of Pharmaceutical Sciences, College of Pharmacy, Princess Nourah bint Abdulrahman University, P.O. Box 84428, Riyadh 11671, Saudi Arabia; aaalsfouk@pnu.edu.sa; 8 Department of Pharmaceutical Sciences, College of Pharmacy, AlMaarefa University, Riyadh 13713, Saudi Arabia; ikaeed@mcst.edu.sa; 9 Pharmaceutical Medicinal Chemistry & Drug Design Department, Faculty of Pharmacy (Boys), Al-Azhar University, Cairo 11884, Egypt; ibrahimeissa@azhar.edu.eg; 10 Pharmacognosy and Medicinal Plants Department, Faculty of Pharmacy (Boys), Al-Azhar University, Cairo 11884, Egypt; 11 Biopharmaceutical Product Research Department, Genetic Engineering and Biotechnology Research Institute, City of Scientific Research and Technological Applications, Alexandria 21934, Egypt

**Keywords:** *Artemisia glauca*, jusan coumarin, new dicoumarin, COVID-19 main protease, molecular similarity, structure fingerprint, DFT, ADMET, toxicity, molecular dynamics

## Abstract

A new dicoumarin, jusan coumarin, (**1**), has been isolated from *Artemisia glauca* aerial parts. The chemical structure of jusan coumarin was estimated, by 1D, 2D NMR as well as HR-Ms spectroscopic methods, to be 7-hydroxy-6-methoxy-3-[(2-oxo-2H-chromen-6-yl)oxy]-2H-chromen-2-one. As the first time to be introduced in nature, its potential against SARS-CoV-2 has been estimated using various in silico methods. Molecular similarity and fingerprints experiments have been utilized for **1** against nine co-crystallized ligands of COVID-19 vital proteins. The results declared a great similarity between Jusan Coumarin and **X77**, the ligand of COVID-19 main protease (PDB ID: 6W63), M^pro^. To authenticate the obtained outputs, a DFT experiment was achieved to confirm the similarity of **X77** and **1**. Consequently, **1** was docked against M^pro^. The results clarified that **1** bonded in a correct way inside M^pro^ active site, with a binding energy of −18.45 kcal/mol. Furthermore, the ADMET and toxicity profiles of **1** were evaluated and showed the safety of **1** and its likeness to be a drug. Finally, to confirm the binding and understand the thermodynamic characters between **1** and M^pro^, several molecular dynamics (MD) simulations studies have been administered. Additionally, the known coumarin derivative, 7-isopentenyloxycoumarin (**2**), has been isolated as well as β-sitosterol (**3**).

## 1. Introduction

Natural products as a base for human treatment were and still are the most productive source [1,2]. Several plants [3,4] and microorganisms [5,6] have been deeply investigated for their healing potential. The curative effect of natural products has been associated with the existence of secondary metabolites such as flavonoids [7,8], isochromenes [9], α-pyrones [10], diterpenes [11], sesquiterpenes [12,13,14], steroids [15], alkaloids [16], and saponins [17,18].

The chemical composition of several plants of the genus *Artemisia* L. was studied and proved the presence of interesting metabolites such as epi-ashantin in *Artemisia sieversiana* [19], hydroaustricin in *Artemisia albida* [20], matricarin in *Artemisia austriaca* [21], and cubreva lactone and cirsineol in *Artemisia umbrosa* [22]. Additionally, sesquiterpene lactones have been isolated from various species as *Artemisia tschernieviana* and *Artemisia sublessingiana* [23]. Further, flavonoids were recorded in *Artemisia albida* [24] and *Artemisia santolinifolia* [25], as well as some *Artemisia* species growing in the Altai Republic and Republic of Khakassia [26]. Likely, the composition and bioactivities of essential oils were discussed for diverse species such as *Artemisia kasakorum* [27], *Artemisia lercheana*, *Artemisia sieversiana* [28,29], *Artemisia umbrosa* [30], five different *Artemisia* species [31], *Artemisia gurganica* [32], *Artemisia proceriformis* [33], *Artemisia terrae-albae* [34], *A. keiskeana* [35], *Artemisia littoricola*, and *Artemisia mandshurica* [36]. *Artemisia commutata* from Mongolia was investigated earlier [37].

*Artemisia glauca* Pall. ex Willd [38] is a perennial herb up to 70 cm high; the shoots are numerous, straight, or rising. The whole plant is felt-pubescent and grayish. Its flower baskets are spherical, on short stems, 1.5–2 mm wide, and collected in a panicled inflorescence. It grows in the steppe zone, in saline wet meadows, in birch forests, along the meadow and rocky slopes of mountains, and along riverbanks, less often as weeds. The general distribution is Europe, eastern and western Siberia, Mongolia, and North America. In Kazakhstan, the area of this species includes the territory of northern and eastern Kazakhstan [39].

Earlier, aromatic acetylenes [40], coumarins [41], heptadeca-1,8 (*cis*), and 16-trien-11,13-diin-15-ol [42] were isolated from *A. glauca*. Similarly, the composition of essential oils of *A. glauca* that grows in Mongolia and Siberia was studied by the GC/MS methods [43,44,45]. On the other hand, the study of the biological activity and chemical composition of *Artemisia glauca* that grows in Kazakhstan was not conducted yet. Various dicoumarin drevatives have been isolated from *Artemisia* L. and exhibited promising bioactivities, such as Arteminorin, the dicoumarin of *Artemisia minor* that showed promising in vitro cytotoxicity against HepG2 cell lines [46]. Additionally, some other dicoumarines of *Artemisia* L. inhibited xanthine oxidase and protein tyrosine phosphatase 1B effectively [47]. Interestingly, the antiviral properties of dicoumarines were reported before [48,49].

Computational (in silico, or cheminformatics) chemistry is a widely applied approach in the field of the pharmaceutical industry and drug discovery that explores the molecular properties of a drug and can expect the interaction of that molecule with a specific protein [50]. The computational chemistry was applied vastly and favorably in several reports that targeted COVID-19 [51,52,53,54,55].

Computational (in silico) tools are essential tools to find lead compounds early in the drug discovery process. Similarity measuring methods are of the most beneficial tools in this particular. The effectiveness of similarity methods vary highly from a bioactivity to another, in a way making it is hard to predict surely. Moreover, any two similarity methods mostly select unlike subsets of actives from a database. Accordingly, it is highly recommended to use more than one similarity method where possible to confirm your results [56,57]. On the other hand, the supposition that compounds that are similar in chemical structure should display alike biological effect is valid in general [57,58]. The best phrase to describe this supposition is that compounds have ‘neighborhood behavior’ [57]. Although, several shocking structure-activity relationships demonstrated significant different biological activities for chemically very similar molecules. Sometimes, optical enantiomers exhibit different biological effects. The difference and similarity of compounds depend on the 3D chemical structure as well as the properties of the binding site in the biological target, not on any unnatural factors [59].

The literature survey indicated that coumarin and bicoumarin scaffolds have promising antiviral activities different types of viruses. For example, several furanocoumarins, namely psoralen, bergapten, imperatorin, heraclenin, heraclenol, saxalin, and oxepeucedanin, inhibited HIV with EC_50_ values of 0.1, 0.354, <0.10, 2.37, 0.115, 2.25, and 1.0 μg/mL, respectively [60,61,62]. In addition, angelicin a furanocoumarin derivative, was reported to inhibit influenza viruses A and B [63]. Furthermore, aesculetin, a dihydroxy coumarin, inhibited HIV with an ED_50_ value of 2.51 μg/mL [64]. Accordingly, we examined the potential inhibitory of **1** against SARS CoV-2. The outcomes of this study will open new insight to reach a promising anti-SARS CoV-2 candidate after deep biological testing at the molecular level.

We here in this research report the isolation as well as the structure elucidation of the new dicoumarin, jusan coumarin (jusan is derived from the Kazak name of the source plant), from *A.* *glauca* aerial parts collected from the east region of Kazakhstan (the Altai Mountains) in addition to two other known metabolites. Since jusan coumarin is introduced for the first time in nature, its anti-COVID-19 potential was estimated. Furthermore, ADMET, and toxicity profiles of jusan coumarin have been examined. Finally, molecular dynamics simulations studies auspicated the accurate binding and interaction of jusan coumarin against M^pro^.

## 2. Results and Discussion

### 2.1. Phytochemistry

#### 2.1.1. Extraction and Isolation

To study finely, the raw material of *A. glauca* (above-ground part, 1.04 kg) was extracted with the chloroform 3 times at boiling temperature. The extracts were combined and made up of 70% ethanol. The ethanol extract was evaporated, and 20 g of extract was obtained. The total extract was subjected to chromatographic separation on a silica gel column (1:20) and eluted from 100% heptane to 100% ethyl acetate and, then, to 100% of MeOH. Fractions of 359 mL were collected and evaporated on a rotary evaporator. The total number of fractions was 89.

Fractions were monitored by thin-layer chromatography, with visual control using an ultraviolet lamp and spraying with anisaldehyde. At elution with heptane-ethyl acetate (7:3) led to the isolation of compound **3**. Further elution with a heptane-ethyl acetate system (5:1), a 265 mg of compound **2** was obtained. Upon further elution with a heptane-ethyl acetate system (1:1) followed by the purification using Sephadex LH-20, 27 mg of a yellow crystalline solid, **1**, was isolated.

#### 2.1.2. Compounds Identification

**Figure 1:**
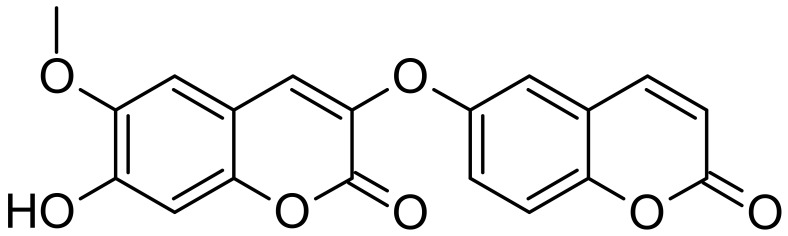
Chemical structure of jusan coumarin.

Compound **1** (Figure 1) showed an m.p. of 235–237 °C. The ^1^H NMR spectrum of **1** showed eight different aromatic signals, three of them were singlets at δ_H_ 6.86 (H-4), δ_H_ 7.87 (H-5), and δ_H_ 7.20 (H-8). Protons 13 and 14 appeared as two doublet signals with *J* = 9.6 Hz at δ_H_ 6.37 and 8.03, respectively. Furthermore, Protons 15, 17, and 18 resonated as an ABX spin system at δ_H_7.18 d (*J* = 2.8 Hz), 7.11 dd (*J* = 2.8 and 8.4 Hz), and 7.70 d (*J* = 8.4 Hz), respectively.

**Figure 2:**
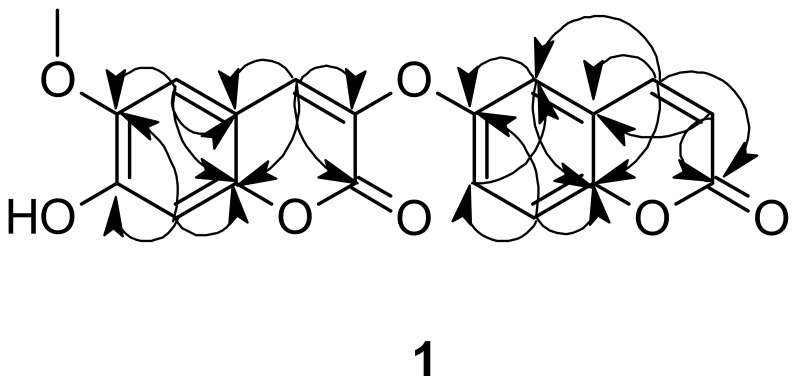
Key HMBC correlations of jusan coumarin.

Finally, a methoxy and a hydroxy group were detected as singlet peaks at δ_H_ 3.88 and 10.3, respectively. The ^13^C spectrum (Table 1) demonstrated 19 carbon signals, all of them were detected in the aromatic region apart from one methoxy at δ_C_ 55.98 and two conjugated carbonyls at δ_C_ 156.95 and 159.95 that assigned for C-2 and 12, respectively. The two carbonyls were found to be highly up fielded due to the strong resonance effect that counteracts the carbonyls’ anisotropic effect. The HSQC and HMBC (Table 1 and Figure 2) spectra assigned every proton to its carbon. The HMBC spectral data confirmed the proposed structure and indicated the attachment of the methoxy group to the carbon atom C-6 (δ_C_147.39). The molecular formula C_19_H_12_O_7_ was determined using HR-ESI-MS experiment, (-ve mode), that showed a pseudo molecular ion peak [M - H]^−^ at *m*/*z* 351.0511 (calcd. for C_19_H_11_O_7_, 351.0504). Compound **1** was verified as a dicoumarin because of two reasons. Firstly, the exact ^1^H NMR integration values of the protons on each side. Secondly, the presence of only one hydroxyl group at δ_H_ 10.3.

**Figure 3:**
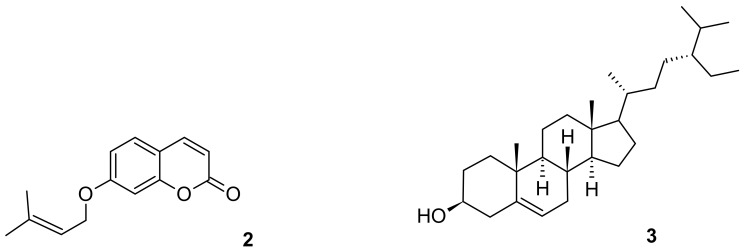
Chemical structures of compounds **2** and **3**.

Compound **2** was obtained as white crystals with m.p. 67–71 °C, ion [M - H] with *m*/*z* 234. Its structure was determined by ^1^H and ^13^C NMR spectroscopy (Table S1). Following the data obtained, compound **2** was identified as 7-isopentenyloxycoumarin (Figure 3), which was previously isolated from *Isocoma pluriflora* [65], *Ferula* species [66], *Notopterygium incisum*, and *Notopterygium franchetii* [67]. Compound **3** was identified as β-sitosterol depending on its m.p., mass, and ^1^H NMR spectral data compared to the published data [68].

### 2.2. Molecular Similarity Study

To figure out the basics of the molecular similarity study, we must refer to the concept that the activity of a molecule resulted from well-studied interactions with certain enzyme target. These enzyme-ligand interactions take place through chemical and physical interactions as hydrophobic and hydrogen bonds interactions. Accordingly, the likeness in the structures of two molecules is expected to cause a likeness in the configuration of H-bond acceptors, donors, and hydrophobic moieties besides the steric configuration. Consequently, a likeness in biological activity is expected too [69].

**Figure 4:**
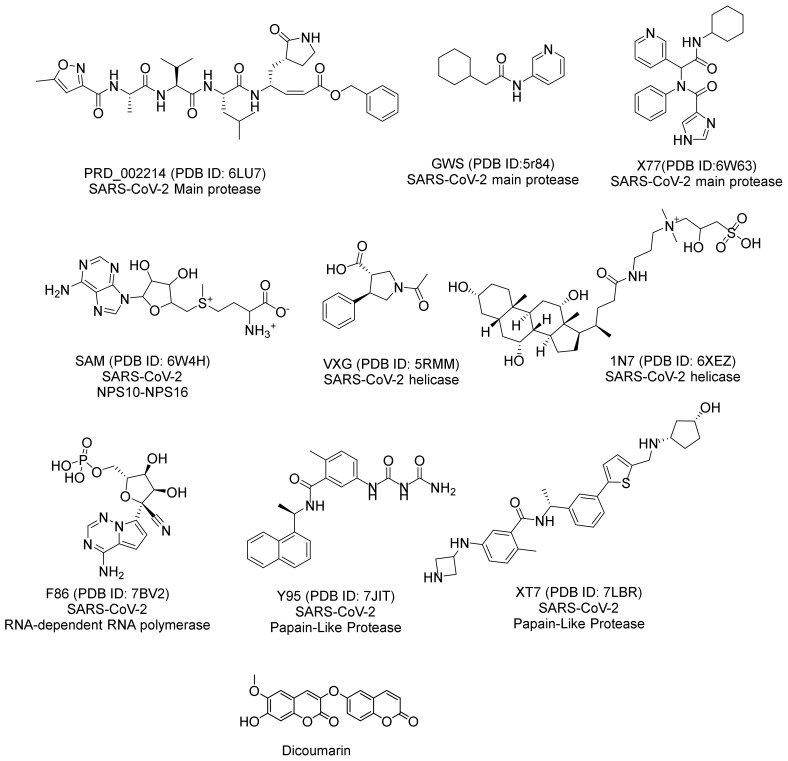
The chemical structures of the examined SARS-CoV-2 proteins ligands and jusan coumarin.

The chemical structure of jusan coumarin was examined against the chemical structures of the nine co-crystallized ligands of the nine SARS-CoV-2 proteins (Figure 4) in a structural similarity experiment. This experiment aims to explore if jusan coumarin has an inhibitory potential against COVID-19.

**Figure 5:**
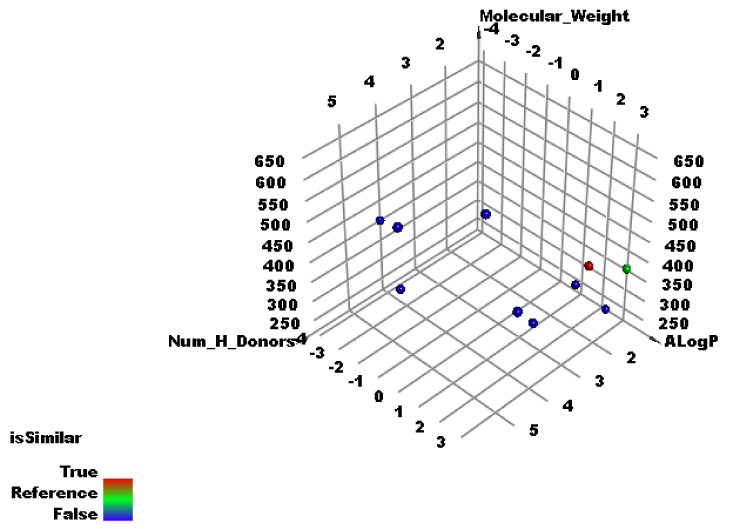
The results of similarity analysis of the examined ligands of SARS-CoV-2 proteins and jusan coumarin.

The Discovery Studio software investigated the subsequent molecular descriptors in jusan coumarin and the examined ligands; the partition coefficient, ALog p, [70]; the molecular weight, M. W, [71]; the number of atoms that act as H- bond donors (HBD) [72]; and H- bond acceptors (HBA) [73]; the rotatable bonds numbers [74]; the aromatic rings numbers [75]; and the heterocyclic rings numbers [76] together with the molecular fractional polar surface area (MFPSA) [77]. The outputs demonstrated the great degree of similarity between jusan coumarin and the co-crystallized ligands (**X77**) of SARS-CoV-2 main protease (PDB ID: 6W63) (Table 2 and Figure 5).

### 2.3. Fingerprint Study

The fingerprint is a second similarity approach that computes the structures of two various molecules or more as 2D after converting to the binary format. The fingerprints study examines the presence or deficiency of one or more of the subsequent features: the charges [78], the hybridization [79], H-bond donors as well as acceptor [80], negative as well as positive ionizables [81], halogens [82], aromatics [83], and the ALog p [84]. The study was conducted employing Discovery Studio. The results confirmed the considerable fingerprint similarity of jusan coumarin and **X77** (Table 3).

### 2.4. Pharmacophore Study

The pharmacophore recognizes the key features in a ligand to interact with a protein target, resulting in elicitation or blockage of a certain biological activity. The 3D-pharmacophore model determines the essential chemical feature of a molecule to be active against a specific protein. Additionally, it specifies the 3D geometry of that essential features [85]. The generated 3D model is an important key that can be used to predict definite bioactivity based on the presence or absence of these features [86,87].

In the presented study, the key pharmacophoric features of the co-crystallized ligands (**X77**) of SARS-CoV-2 main protease (PDB ID: 6W63) were determined using an auto-generated pharmacophore protocol in Discovery Studio 4.0. Then, the jusan coumarin was tested to fit with the generated pharmacophore model.

**Figure 6:**
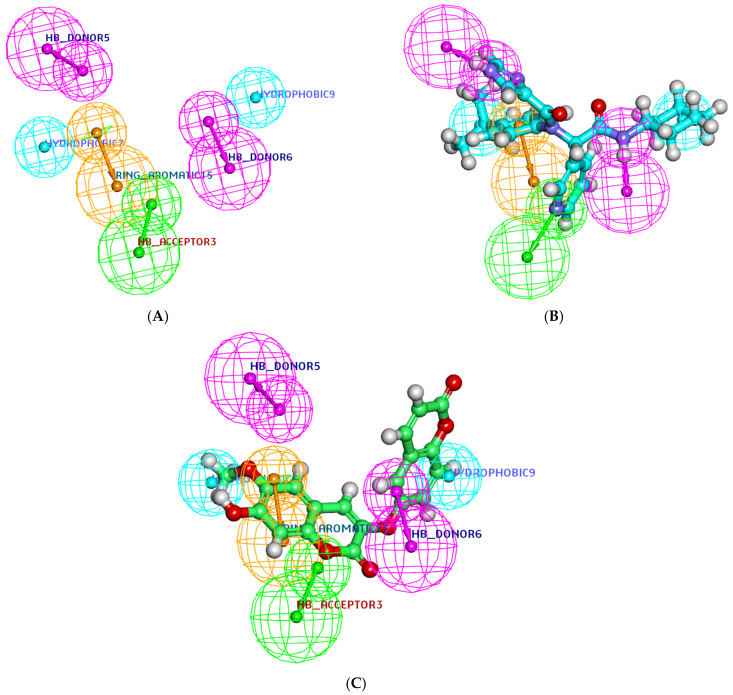
(**A**) The generated 3D-pharmacophore geometry with six features; two H-bond donors (pink), one H-bond acceptor (green), one ring aromatic (brown), and two hydrophobic centers (blue). (**B**) Mapping of the co-crystallized ligand (**X77**) on the generated pharmacophore (fit value = 2.08). (**C**) Mapping of the jusan coumarin on the generated pharmacophore (fit value = 1.98).

The generated 3D pharmacophore model consisted of three features: two H-bond donors, one H-bond acceptor, one ring aromatic, and two hydrophobic centers (Figure 6A). The generated model was used as a 3D search query to evaluate the jusan coumarin as a similar compound to **X77**. The fitting of **X77** against the generated pharmacophore model was illustrated in Figure 6B. **X77** showed a Fit value of 2.08 against the generated pharmacophore. The jusan coumarin was mapped on the generated 3D-pharmacophore model. The results indicated that the jusan coumarin has the main essential features of **X77**. The jusan coumarin showed a fit value of 1.98. This value almost equal to that of **X77**, indicating the high similarity between the tested coumarin and **X77** (Figure 6C).

### 2.5. DFT Studies

DFT is an advanced computational technique that computes the molecular orbital analysis as well as the molecular electrostatic potential maps of a molecule depending on some parameters (HOMO, LUMO, gap energy, and total energy, besides a dipole moment) [88,89].

The used functional was the PWC of local density approximate (LDA) [90]. Additionally, the quality was adapted as Coarse, which utilizes the DN bases set and SCF density converge of 1.0 × 10^−4^, as employed from Acclrys in the DMl3 module of the Materials Studio package [91,92].

**Figure 7:**
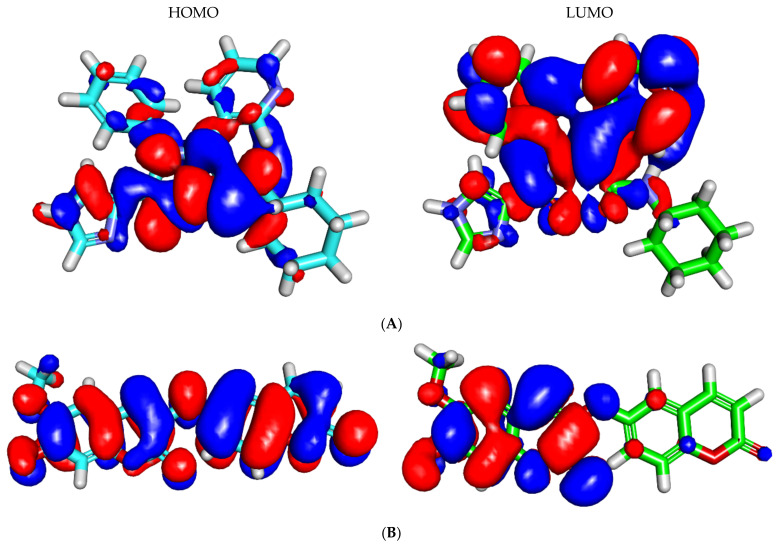
Spatial distributions of molecular orbitals for (**A**) jusan coumarin and (**B**) **X77**.

**Figure 8:**
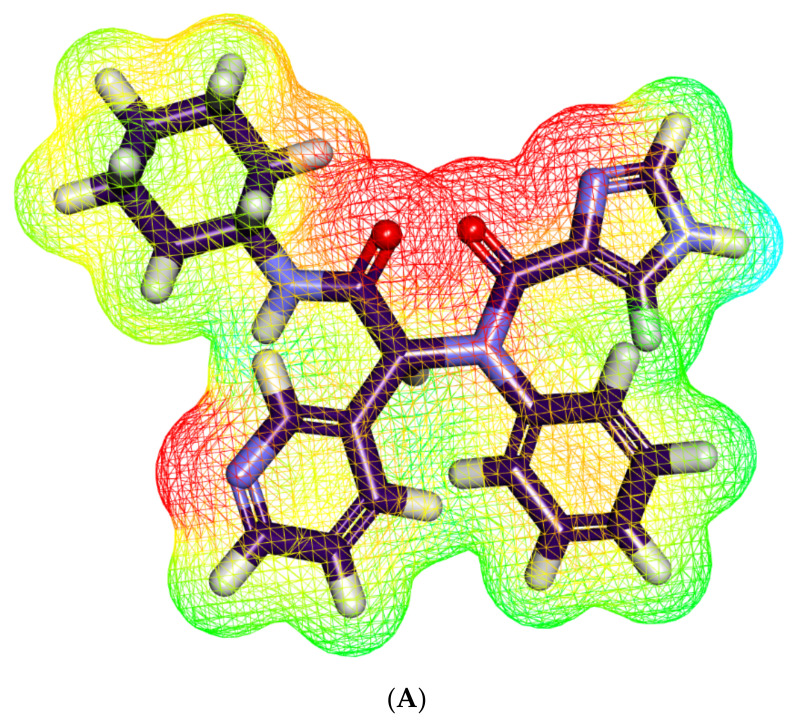

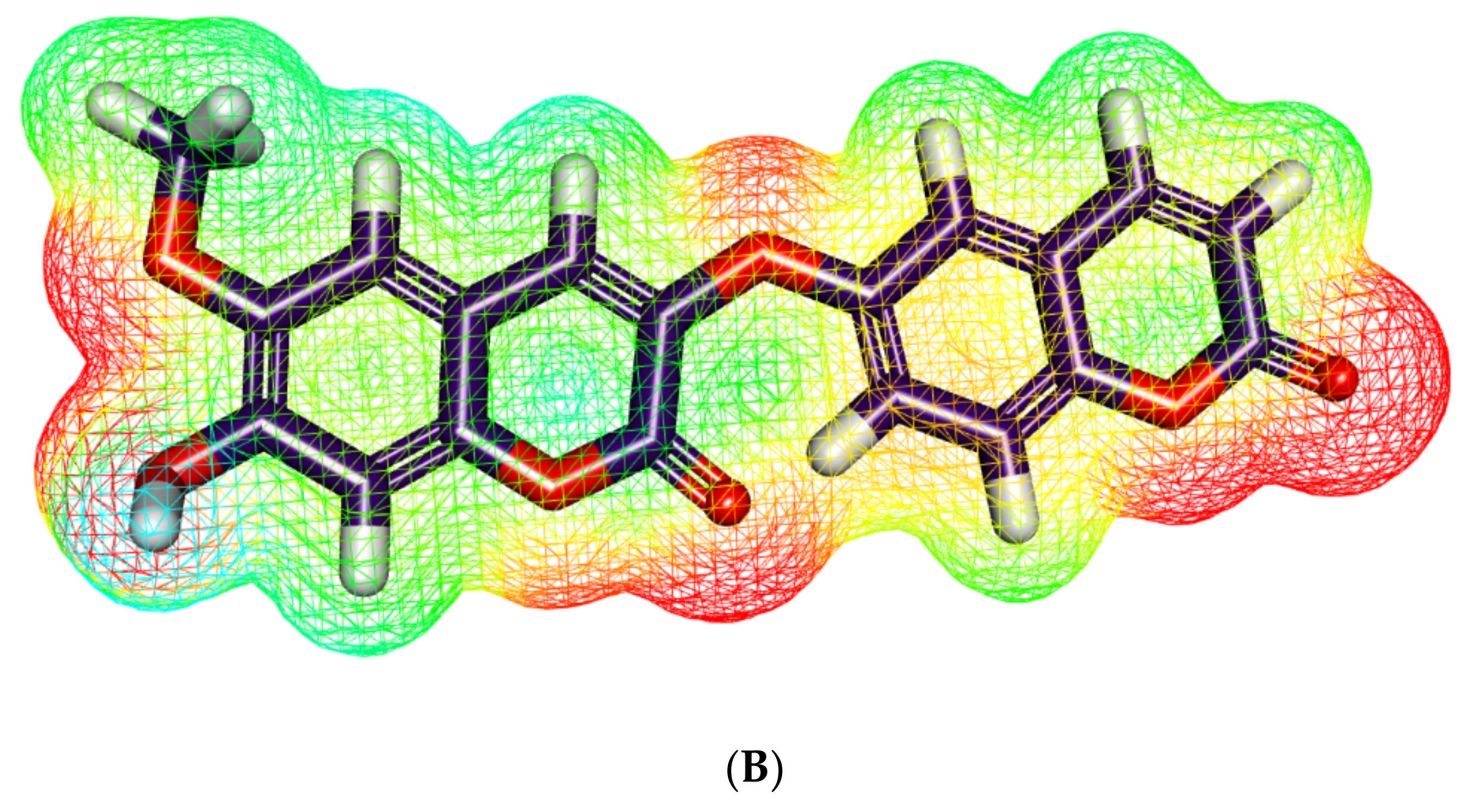
Molecular electrostatic potential map of (**A**) **X77** and (**B**) jusan coumarin. The red zones refer to the electronegative atoms that can participate as hydrogen acceptors; the blue zones refer to the electron-poor atoms that can participate as hydrogen bonds donors; and the yellow and green zones refer to the neutral atoms that can form hydrophobic interactions. **X77** has four red zones, one blue zone, and four yellow zones. Jusan coumarin has eight red zones, two blue zones, and three yellow zones.

The DFT experiment gives close sight of the degree and type of reactivity of a ligand. Consequently, the DFT parameters of jusan coumarin and **X77** were investigated using the Discovery Studio software to discover the resemblance between them on this side. The outputs were outlined in Table 4, as well as Figure 7 and Figure 8.

#### 2.5.1. Molecular Orbital Analysis

Jusan coumarin and **X77** exhibited total energy values of −1247.389 and −1304.024 hartree, respectively. From these results, jusan coumarin has total energy almost equal to that of **X77**. Jusan coumarin and **X77** showed closely equal values of dipole moment (4.116 and 3.061). Furthermore, the gap energy of jusan coumarin (0.083 hartree) was lower than that of **X77** (0.094 hartree).

#### 2.5.2. Molecular Electrostatic Potential Maps (MEP)

MEP is a computational technique that computes electronegativity, partial charges in addition to the chemical reactivity to discover the electrostatic potential of a molecule in the 3D form. [93]. MEP gives deep insight into the way that a certain drug interacts with a receptor [94]. In MEP, the electronegative atoms that can participate as an acceptor in hydrogen-bonding interactions with the receptor, colored with red. On the other hand, the electron-poor atoms that can form hydrogen bonds with the receptor as a donor colored with blue. Finally, the neutral atoms that can form hydrophobic interactions colored with green to yellow [95].

The MEP of jusan coumarin and **X77** were described in Figure 8A and Figure 8B, respectively. The mentioned figures indicate that **X77** has four red patches, which are suitable for H-bond acceptors, and a blue patch, which is suitable for H-bond donors. In addition, there are yellow patches on both aliphatic and aromatic moieties, indicating a high possibility for hydrophobic interactions. For jusan coumarin, it has eight red patches, which are suitable for bonding-bond acceptors, and two blue patches, which are suitable for H-bond donors. Additionally, there is a yellow patch on the aromatic system giving a chance for hydrophobic interaction. These outcomes indicate the high possibility of jusan coumarin interacting with the target receptor in a similar way to **X77**.

### 2.6. Docking Studies

To verify the accomplished results, the interaction of jusan coumarin was investigated through molecular docking studies against M^pro^ using the co-crystallized ligand, **X77**, as a reference. Binding free energy (∆G) between jusan coumarin and M^pro^, besides the binding modes, were the basics of activity determination.

**Figure 9:**
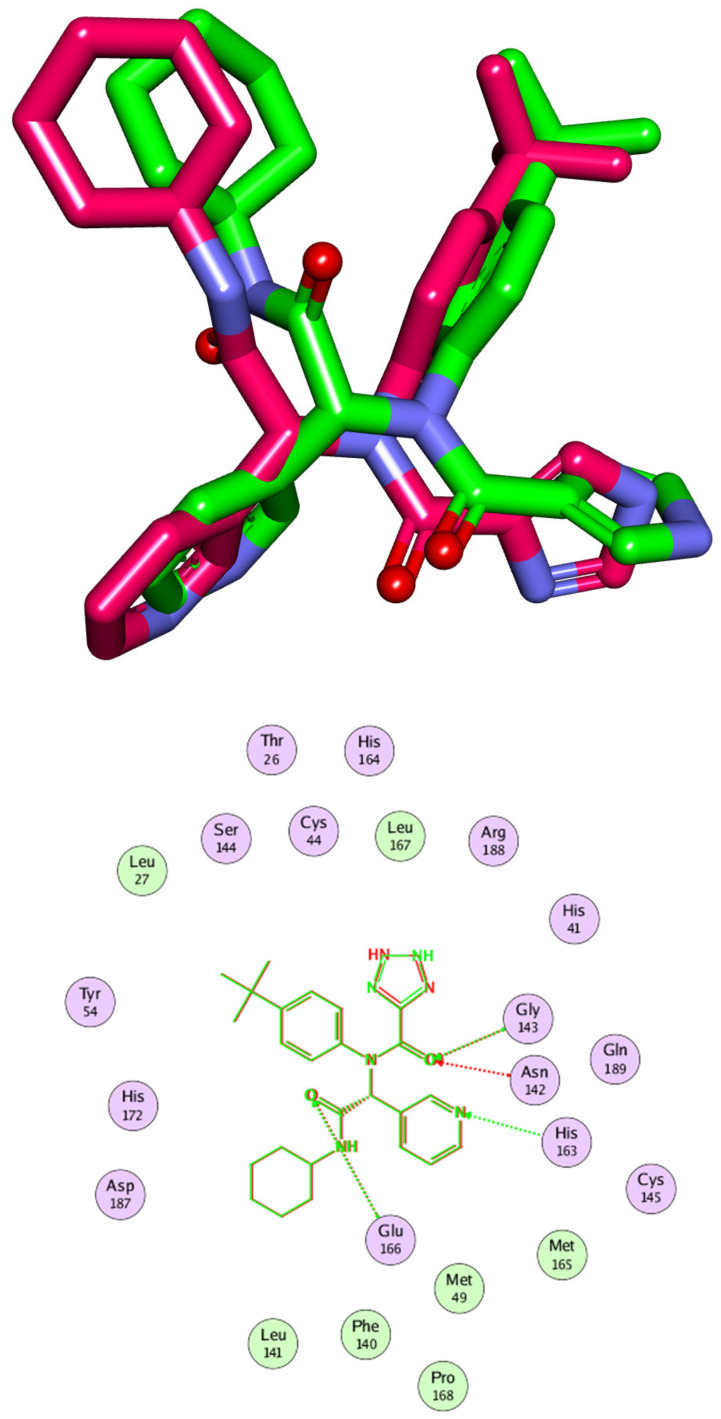
3D and 2D Superimpositions of **X77** (pink) and the docking pose (dark green) of the same molecule.

Firstly, a verification process of the utilized docking methods was carried out by repeating the docking for **X77** against M^pro^. The calculated RMSD value was 1.8 Å, indicating the validness of the employed docking process (Figure 9).

**Figure 10:**
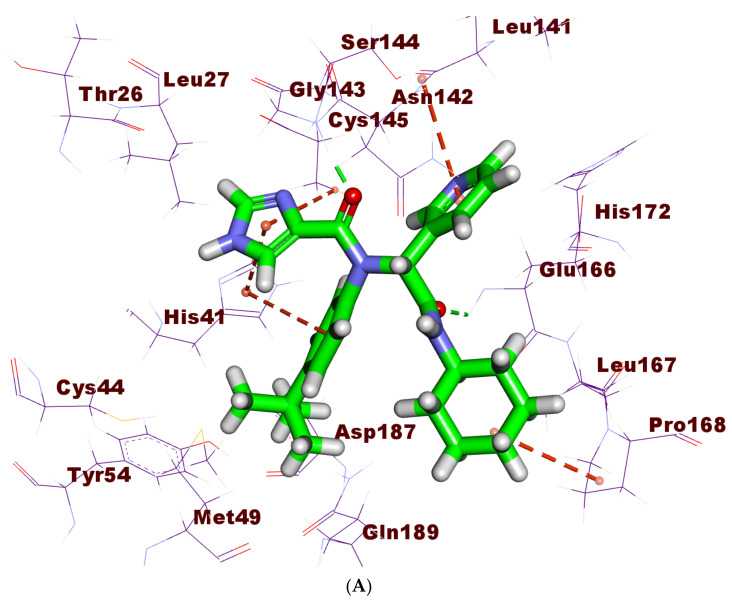

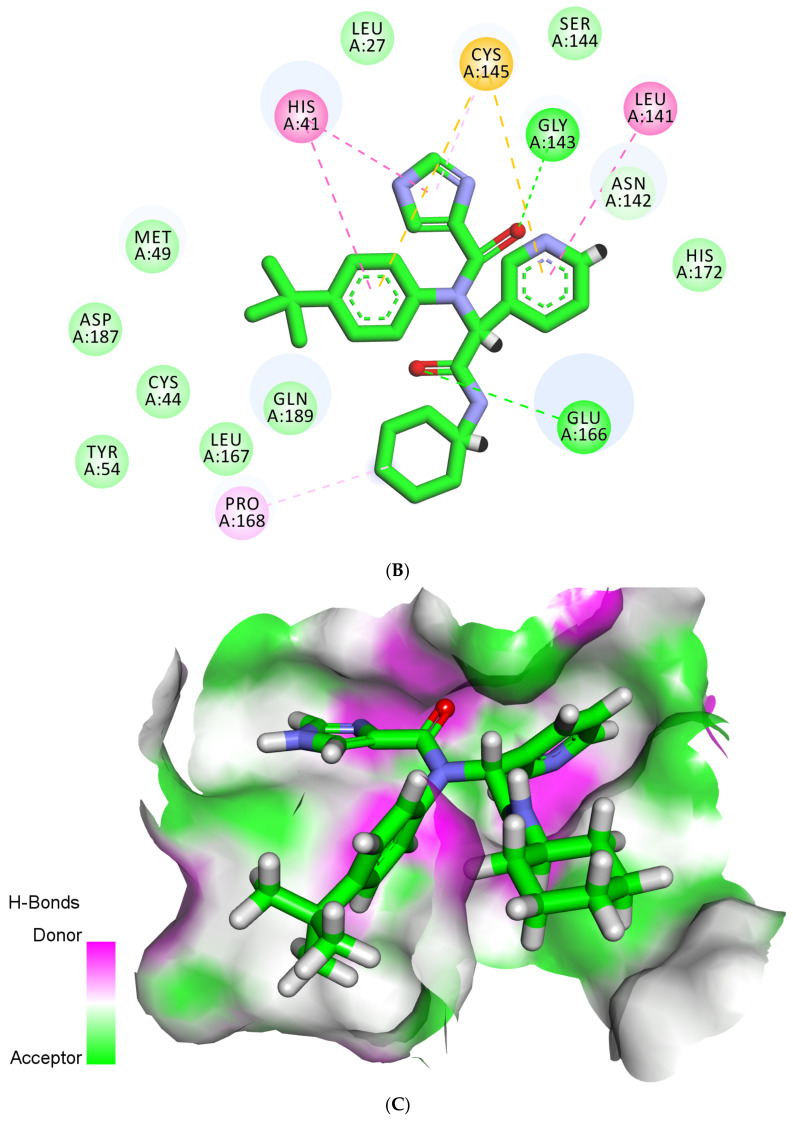
(**A**) 3D, (**B**) 2D, and (**C**) Surface mapping of **X77** docked into the active site of M^pro^ The binding mode of jusan coumarin showed a binding free energy of −18.45 kcal/mol. It occupied two pockets. The 2H-chromen-2-one moiety was found inside the first pocket of the receptor to form two hydrophobic interactions with Leu141 andCys145. The hydrophobic side of the 2H-chromen-2-one moiety showed high contact with His163, His172, and Phe140. In addition, it formed a hydrogen bond with Leu141. The 7-hydroxy-6-methoxy-2*H*-chromen-2-one moiety occupied the third pocket, forming two hydrogen bonds with Gly143 and Ser144. Furthermore, it exhibited four hydrophobic interactions with His41 and Cys145. The linker oxygen atom formed one hydrogen bond with Met165 (Figure 11).

The binding free energy of **X77** against the active site of M^pro^ was −21.61 kcal/mol (Table 5). The first pocket of M^pro^ was occupied by the pyridine nucleus, which formed two hydrophobic contacts with Cys145 and Leu141. The second pocket was occupied by the 1H-imidazole, which formed two hydrophobic contacts with the amino acids Cys145 and His41. The tert-butylbenzene moiety was found inside the third pocket, where it was in close proximity to Arg188, Met49, and Leu167. With Cys145 and His41, it formed two hydrophobic connections. The cyclohexyl moiety was found inside the fourth pocket, adjacent to Pro168. With Glu166, the amide moiety created one hydrogen bond (Figure 10).

### 2.7. In Silico ADMET Studies

**Figure 12:**
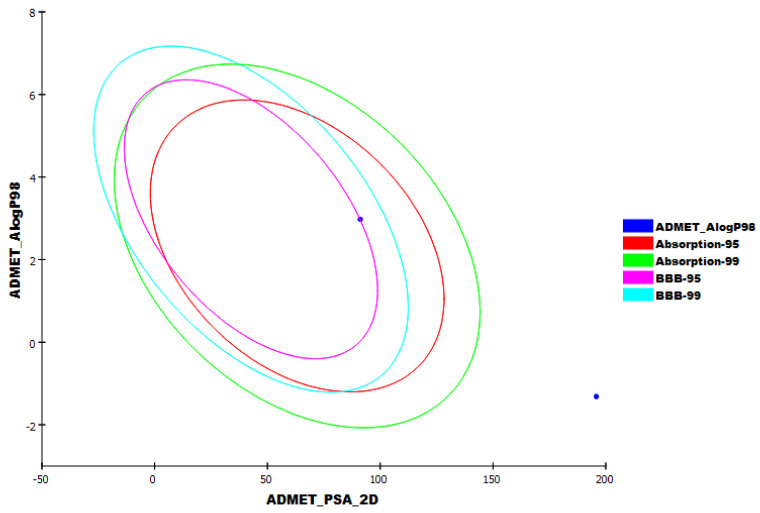
The expected ADMET study.

The in silico ADMET profiles were detected for jusan coumarin using the Discovery Studio software. Simeprevir was exploited as a reference compound. The results were demonstrated in Figure 12. Jusan coumarin was found to have a low ability to penetrate the BBB, which declares its safety against the CNS. Although the aqueous solubility was poor, its intestinal absorption ability was good. Fortunately, jusan coumarin was anticipated to be a CYP2D6 non-inhibitor and was foreboded to bind to the plasma protein by a ratio of less than 90%.

### 2.8. In Silico Toxicity Studies

The in silico toxicity potentialities of jusan coumarin were tested using the Discovery Studio software against seven toxicity models. Remdesivir was used as a reference drug. The results were summarized in Table 6.

Jusan coumarin exhibited a high level of Median carcinogenic potency (TD_50_), maximum tolerated dose (MTD), rat oral lethal dose (LD_50_), and rat chronic lowest observed adverse effect level (LOAEL). Similarly, the skin and ocular irritancy abilities of jusan coumarin were computed to be mild.

### 2.9. Molecular Dynamics Simulations Studies

**Figure 13:**
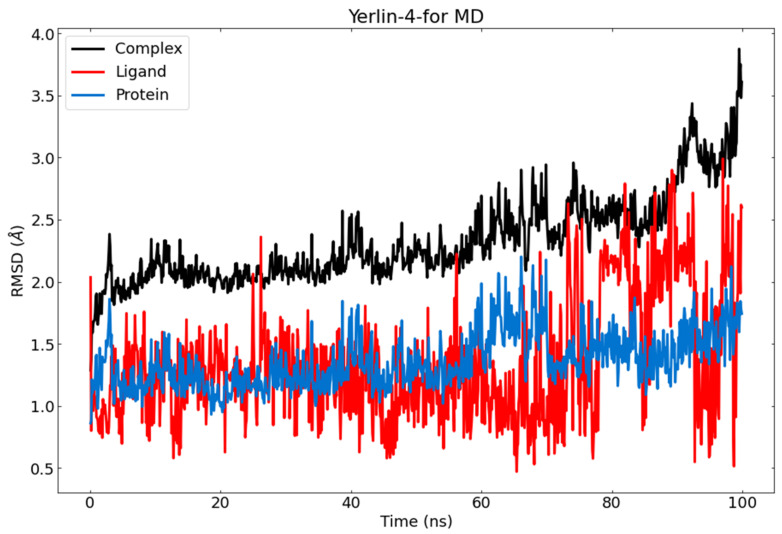
RMSD value of jusan coumarin in MD runs. Red: jusan coumarin; blue: M^pro^; black: M^pro^-jusan coumarin complex.

Molecular dynamics (MD) simulations were conducted to compare the binding stability of the M^pro^-jusan coumarin complex; after doing the molecular docking, ligand 4D show more stability and binding by HB than other ligands. MD simulation has been done to ensure the stability of it, with ligand 4D showing a good RMSD value along 100 ns MD, and the target protein shows a root-mean-square deviation (RMSD) value of 1.25 Å too, while the M^pro^-jusan coumarin complex exhibits an RMSD value of 2.25 Å (Figure 13), which is below the acceptable range around 4 Å. It has proven M^pro^-jusan coumarin complex is stable in a 100 ns MD simulation.

**Figure 14:**
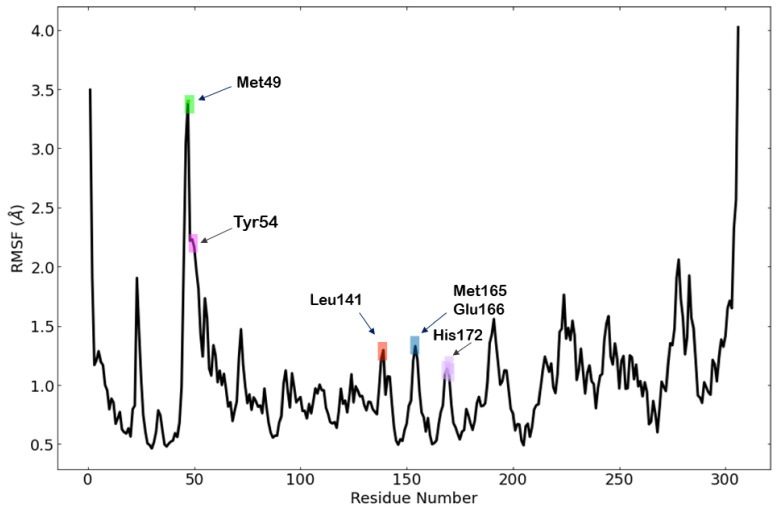
Per residue, RMSF for M^pro^ in the MD run.

Root mean square fluctuation (RMSF) is an essential mean that describes the flexibility differences among the M^pro^-jusan coumarin complex during the MD simulation for 100 ns. Figure 14 shows the root-mean-square fluctuation (RMSF) values of M^pro^ during the MD run. The Root Mean Square Fluctuations (RMSF) of M^pro^ showed that Met49 residue has high level of fluctuation. On the other hand, the residues Tyr54, Leu141, Met165, Glu166, and His172 showed decreased levels of fluctuation. Accordingly, it can be said that the residual fluctuation of M^pro^ was stable upon the binding of jusan coumarin, without major variations. Such results indicate the stability of M^pro^ during the study.

**Figure 15:**
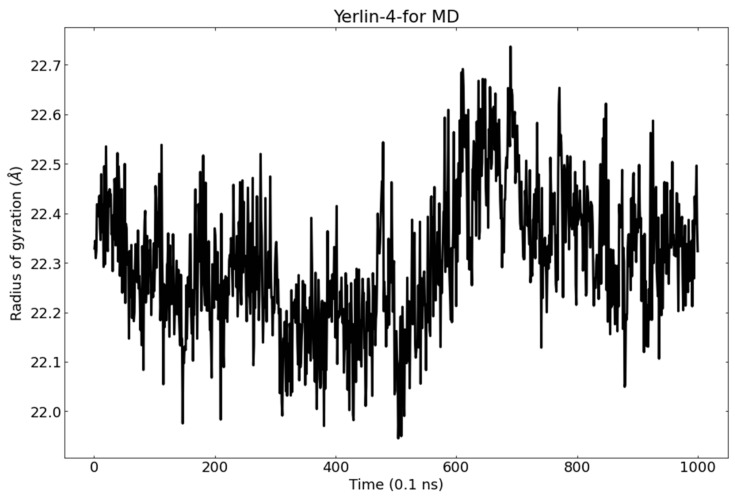
The radius of gyration of M^pro^ in the MD run.

The radius of gyration (R_g_) gives a clear idea about the protein’s volume change and hence its stability [96,97]. Consequently, the analysis of R_g_ of M^pro^ during the MD simulation has been studied and suggested the tight packing of M^pro^ in the binding state to jusan coumarin. M^pro^-jusan coumarin complex reached a stable conformation with the R_g_ fluctuating around 24.4 Å (Figure 15).

**Figure 16:**
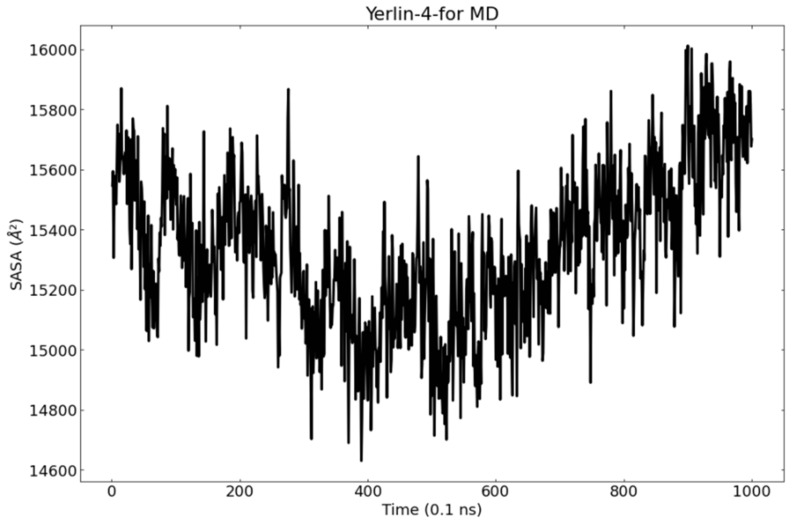
SASA of M^pro^ in the MD run.

The solvent-accessible surface area (SASA) evaluation gives an idea about the conformational changes in a protein after a ligand binding [98]. The average SASA values for M^pro^ in the binding state with jusan coumarin were evaluated during the 100 ns MD simulations. The outcomes showed no major changes indicating the stability of the examined complex (Figure 16).

The obtained MD out puts confirm the stability of M^pro^-jusan coumarin complex that exhibited low RMSD and RMSF values through 100 ns of the run. Additionally, the M^pro^ was confirmed to be persistent showing no major fluctuations through the exhibition of low R_g_ and SASA values. The achieved findings are consistent with the proved similarity as well as the high binding affinity that was exhibited in the molecular docking.

## 3. Experimental

### 3.1. General Experimental Section

NMR were performed on (Bruker Avance 600 and 300 MHz), the chemical shifts (δ) were provided in parts per million (ppm) regarding the reference, tetramethylsilane (TMS), and (^1^H) or (^13^C) signals of deuterated solvents. Spin-spin coupling constants (*J*) are displayed in hertz (Hz). The ^13^C NMR spectra signals were refined using the Distortion less Enhancement by Polarization Transfer (Dept), the Heteronuclear Single Quantum Coherence (HSQC), and the Heteronuclear Multiple Bond Correlation (HMBC). Mass spectra were achieved on an HP599A apparatus (EI and CI, ionization energy of 70 eV) employing Apolo 300 data and on a Krotas MS0TC instrument for precise calculation (reaching by electric shock (ESI), solvent mixture: CH_2_Cl_2_-MeOH + NH_4_OAc) with MASLYNX system data. The UV spectra were recorded on a Perken-Elmer Lambda 20 Spectrometr. The Melting points were recorded on a Reichirt Thermavar. Regarding column chromatography, silica gel with 0.06–0.2 mm (Acros) aswell as Sephadex LH-20 were utilized as stationary phases. Additionally, silica gel with a mesh of 32–63 was utilized for flash column chromatography.

### 3.2. Plant Material

The areal parts of *Artemisia glauca* Pall. ex Willd., (wormwood gray, family Asteraceae), were collected from the east Kazakhstan region, the Altai Mountains.

Species were identified by employees of Altai Botanical Garden (Reader city, Kazakhstan). A sample (herbarium) was stored under the code of 2007.09.06.03.12 in the International Scientific Research Holding “Phytochemistry” Fund.

### 3.3. Extraction and Isolation

Total wight of 1.04 kg of plant materials were extracted with chloroform and heated to the boiling point of chloroform in the bottom flask, for three times. The collected solvents were evaporated using a rotary evaporator a water-jet pump under a reduced pressure at 60 °C. The total extract weighed 20 g and was utilized for preparative chromatographic separations by several columns on silica gel and Sephadex LH-20.

### 3.4. Molecular Similarity

The molecular similarity of jusan coumarin against nine essential co-crystallized ligands of SARS-CoV-2 was investigated employing Discovery Studio 4.0 (See Supplementary Materials).

### 3.5. Fingerprint Study

A fingerprints study of jusan coumarin against nine essential co-crystallized ligands of SARS-CoV-2 was investigated employing using Discovery Studio 4.0 (See Supplementary Materials).

### 3.6. DFT

The DFT parameters of jusan coumarin were investigated employing Discovery Studio [99] (See Supplementary Materials).

### 3.7. Docking Studies

The docking investigation was done for jusan coumarin employing MOE2014 software and visualized using Discovery Studio 4.0 [100,101,102] (See Supplementary Materials).

### 3.8. ADMET

ADMET descriptors of jusan coumarin were investigated employing Discovery Studio 4.0. [103,104] (See Supplementary Materials).

### 3.9. Toxicity Studies

The toxicity profile of jusan coumarin was investigated employing Discovery Studio 4.0 [105,106,107] (See Supplementary Materials).

### 3.10. Molecular Dynamics Simulations

The system has been adjusted by the web-based CHARMM-GUI [108,109,110] interface utilizing the CHARMM36 force field [111]. The conducted simulations were done utilizing the NAMD 2.13 [112] package. The TIP3P explicit solvation model was applied [113] (See Supplementary Materials).

## 4. Conclusions

A new dicoumarin, jusan coumarin, (**1**) was isolated from *Artemisia glauca* aerial parts. Jusan coumarin demonstrated a high degree of similarity with **X77**, the co-crystallized ligand of M^pro^. The similarity was confirmed by four ligand-based computational, molecular similarity, fingerprints, DFT, and pharmacophore studies. The molecular docking studies of **1** against M^pro^ verified the perfect binding of **1** inside the active site of M^pro^, exhibiting a binding energy of −18.45 kcal/mol. ADMET and toxicity profiles of **1** showed its overall safety and its likeness to be used as a drug. The MD simulations studies authenticated the binding of **1** inside the M^pro^. These findings give hope to find a cure for COVID-19 upon further in vitro and in vivo studies.

## Figures and Tables

**Figure 11:**
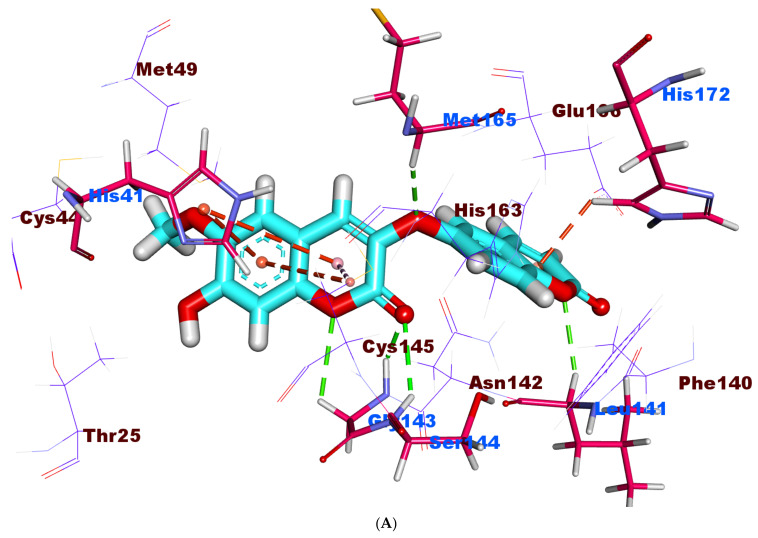

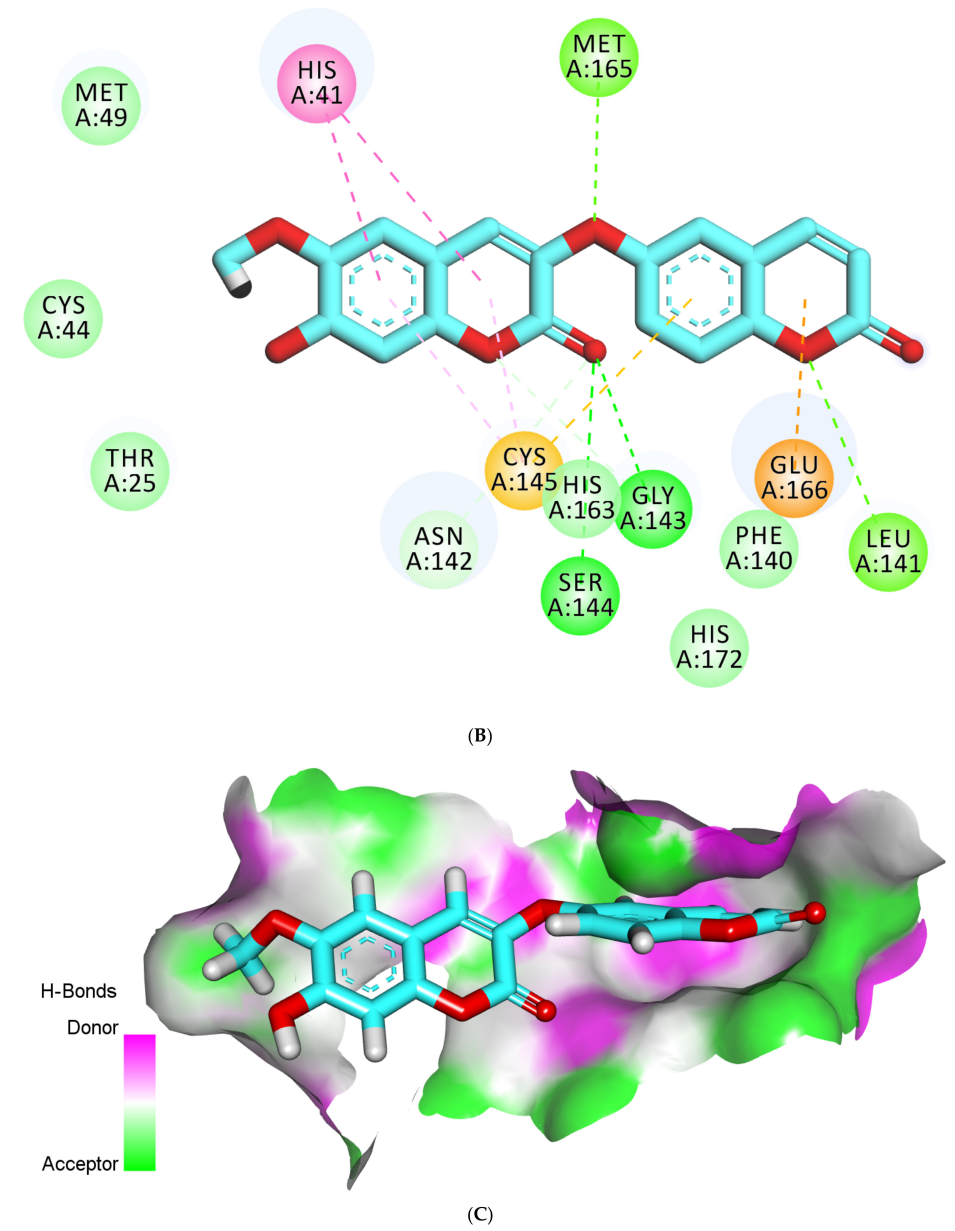
(**A**) 3D, (**B**) 2D, and (**C**) Surface mapping of jusan coumarin docked into the active site of M^pro^.

**Table 1:** ^1^H and ^13^C spectral data of jusan coumarin, **1**, (DMSO, δ).

Position	δH (*J* = Hz)	δC	HMBC
2	-	156.95	
3	-	135.68	
4	7.87 s	130.62	110.16, 135.68, 156.95, 147.39
5	7.2 s	109.09	110.16, 145.64, 130.62, 147.39
6	-	145.64	
7	-	150.32	
8	6.86 s	102.48	147.39, 150.32, 109.09, 145.64
9		147.39	
10		110.16	
12		159.95	
13	6.37 (*J* = 9.6 Hz)	113.61	114.36, 159.95
14	8.03 d (*J* = 9.6 Hz)	143.77	114.36, 103.72, 159.95, 154.98
15	7.18 d (*J* = 2.8Hz)	103.72	114.36, 159.64, 154.98
16	-	159.64	
17	7.11 dd (*J* = 2.8 and 8.4 Hz)	113.15	103.72, 114.36, 159.64
18	7.70 d (*J* = 8.4Hz)	129.62	113.61, 143.77, 154.98, 159.64
19	-	154.98	
20	-	114.36	
21	3.78 s	55.98	145.64
7-OH	10.3 s	-	

**Table 2:** Structural properties of jusan coumarin with **X77**.

Comp.	ALog p	M. Wt	HBA	HBD	Rotatable Bonds	Rings	Aromatic Rings	MFPSA	Minimum Distance
**X77**	2.622	403.477	4	2	6	4	3	0.22	0.644782
**1**	2.975	352.294	7	1	3	4	2	0.286	-

**Table 3:** Fingerprint similarity between jusan coumarin and **X77**.

Comp.	Similarity Factor	S-A	S-B	S-C
**1**	1	361	0	0
**X77**	0.576402	298	156	63

**S-A**: the bits that are present in jusan coumarin and **X77**. **S-B**: the bits that are present in jusan coumarin but not the **X77**. **S-C**: the bits that are present in **X77** but not jusan coumarin.

**Table 4:** The spatial distribution of molecular orbitals for jusan coumarin and **X77**.

Comp.	Total Energy (Ha)	Energy of Binding (Ha)	Energy of HOMO (Ha)	Energy of LUMO (Ha)	Dipole Mag	Band Gap Energy (Ha)
**1**	−1247.389	−8.247	−0.202	−0.119	4.116	0.083
**X77**	−1304.024	−10.798	−0.159	−0.065	3.061	0.094

**Table 5:** Binding free energies (∆G in kcal/mol) of jusan coumarin and the co-crystallized ligand against M^pro^.

Comp.	∆G [kcal/mol]
**1**	−18.45
**X77**	−21.61

**Table 6:** Toxicity profiles of jusan coumarin.

Comp.	TD_50_^a^	MTD ^b^	LD_50_^b^	LOAEL ^b^	Ocular Irritancy	Skin Irritancy
**1**	7.87553	0.117283	0.287784	0.0220943	Mild	Mild
remdesivir	1.01218	0.234965	0.308859	0.0037911	Mild	Mild

^a^ Unit: mg/kg/day. ^b^ Unit: g/kg.

## Data Availability

All data are contained in the published manuscript.

## Supplementary Materials

The following supporting information can be downloaded at: https://www.mdpi.com/article/10.3390/molecules27072281/s1. The NMR aswell as HR-Ms spectral data of jusan coumarin, the toxicity report of jusan coumarin, and the in silico methodology are available online [114,115,116,117,118,119,120,121,122,123]. Table S1: ^1^H and ^13^C spectral data **2** (CDCl3, δ).

## Sample Availability

Samples of the compounds are available from the authors.
