# Supplementary material for: Isolation and In Silico SARS-CoV-2 Main Protease Inhibition Potential of Jusan Coumarin, a New Dicoumarin from Artemisia glauca
Source: Molecules. 2022 Mar 31;27(7):2281. doi: 10.3390/molecules27072281 (PMC9000794; doi:10.3390/molecules27072281)
Supplement: Supplementary file 1 [file molecules-27-02281-s001.zip › molecules-1562081-supplementary.pdf]

Supporting Data

Isolation and *in silico* SARS-CoV-2 main protease inhibition potential of jusan coumarin, a new dicoumarin from *Artemisia glauca*

Suleimen Yerlan M.[1,2], Jose Rani A.[3], Suleimen Raigul N.[4*], Ishmuratova Margarita Yu.[5], Toppet Suzanne[3], Dehaen Wim[3], Aisha A. Alsfouk[6], Eslam B. Elkaeed[7], Ibrahim. H. Eissa[8], Ahmed M. Metwaly[9,10*]

[1]The International Centre for Interdisciplinary Solutions on Antibiotics and Secondary Metabolites, Republican collection of microorganisms, Nur-Sultan, Republic of Kazakhstan

[2] The laboratory of Engineering Profile of NMR Spectroscopy, Sh. Ualikhanov Kokshetau University, Kokshetau, Republic of Kazakhstan

[3]Catholic University of Leuven, Leuven, Belgium

[4] L.N. Gumilyov Eurasian National University, 010010, Nur-Sultan, Kazakstan

[5] Department of Botany, E.A. Buketov Karaganda University, Karaganda, Republic of Kazakhstan

[6]Department of Pharmaceutical Sciences, College of Pharmacy, Princess Nourah bint Abdulrahman University, P.O. Box 84428, Riyadh 11671, Saudi Arabia

[7]Department of Pharmaceutical Sciences, College of Pharmacy, AlMaarefa University, Ad Diriyah 13713, Riyadh, Saudi Arabia

[8]Pharmaceutical Medicinal Chemistry & Drug Design Department, Faculty of Pharmacy (Boys), Al-Azhar University, Cairo11884, Egypt

[9]Pharmacognosy and Medicinal Plants Department, Faculty of Pharmacy (Boys), Al-Azhar University, Cairo, Egypt

[10]Biopharmaceutical Product Research Department, Genetic Engineering and Biotechnology Research Institute, City of Scientific Research and Technological Applications, Alexandria, Egypt

Content

Method	Fingerprint studies
	Molecular Similarity
	Docking studies
	ADMET studies
	Toxicity studies
	Molecular dynamics
Spectral data	
Toxicity report	

Method

1. General experimental section

NMR spectra were carried out on a commercial instrument (Bruker Avance 300 and 600 MHz), chemical shifts (δ) are presented in parts per million (ppm) and re-calculated with respect to tetramethylsilane (TMS) (^{1}H) or carbon signals of deuterium solvents (^{13}C). Spin-spin coupling constants (J) are given in hertz (Hz). Refinement of ^{13}C NMR spectra signals was carried out using Dept, HSQC, HMBC NMR spectra. Mass spectra were recorded on an HP5989A instrument (CI and EI, ionization energy 70 eV) with Apollo 300 data, and on a Kratos MS50TC instrument for accurate calculations (reaching by electric shock (ESI), common solvent mixture: CH_2Cl_2-MeOH + NH_4OAc) with MASSLYNX system data. UV spectra were obtained on a Perkin-Elmer Lambda 20 Spectrometr instrument. Melting points were determined on Reichert Thermovar. For column chromatography, silica gel 0.06-0.2 mm (Acros) was used as the stationary phase. Silica gel 32-63 mesh was used for flash column chromatography.

2. Plant material

To study the component composition of *Artemisia glauca* Pall. ex Willd., or wormwood gray (family *Asteraceae*), the aboveground part is collected in the East Kazakhstan region (Altai Mountains).

Species affiliation is identified by employees of the Altai Botanical Garden (Reader city, eastern Kazakhstan). The herbarium sample is stored in the International Scientific Research Holding «Phytochemistry» Fund. The herbarium sample code is 2007.09.06.03.12.

3. Extraction and isolation

1.04 kg of raw material was placed in a round-bottomed flask and filled with chloroform and heated to the boiling point of solvents. This operation was repeated three times. The solvent was evaporated on a rotary evaporator under the vacuum of a water-jet pump to obtain an extract weighing 20 g, which was used for preparative chromatographic separation by column chromatography on silica gel.

4. Molecular Similarity

Molecular Similarity of the tested compound against the eight co-crystallized ligands of SARS-Cov-2 was carried out calculated using Discovery studio 4.0. At first, the CHARMM force field was applied then the compound was prepared using prepare ligand protocol. Then, the tested compound was used as a reference compound while the co-crystallized ligands were used as a test set. The protocol was adjusted to give one output. The default molecular properties were applied.

The molecular properties include the number of rotatable bonds, number of rings, number of aromatic rings, number of hydrogen bond donors (HBA), number of hydrogen bond acceptors (HBD), partition coefficient (ALog p), molecular weight (M. Wt), and molecular fractional polar surface area (MFPSA).

5. Fingerprint study

A fingerprint study of the tested compound against the eight co-crystallized ligands of SARS-Cov-2 was carried out calculated using Discovery studio 4.0. At first, the CHARMM force field was applied then the compounds were prepared using prepare ligand protocol. Then, the tested compound was used as a reference compound while the co-crystallized ligands was used as a test set. The protocol was adjusted to give the most related co-crystallized ligand to the compound. The default molecular properties were applied. The used fingerprints were based on some parameters related to the type of atoms which may be one of the following: charge, hybridization, H-bond acceptor, H-bond donor, Positive ionizable, Negative ionizable, Halogen, Aromatic, or None of the above. In addition, it includes the ALogP category of atoms.

6. DFT

The DFT parameters (total energy, binding energy, HOMO, LUMO, gap energy, dipole moment, and electrostatic potential) were calculated using Discovery studio software. the tested compounds were prepared using prepare ligand protocol. Then, the prepared compounds were subjected to DFT calculation protocol using the default option

7. Docking studies

Crystal structure of SARS-Cov-2 main protease (PDB ID: 6W63) was obtained from Protein Data Bank. The co-crystallized ligand **X77**, (N-(4-tert-butylphenyl)-N-[(1R)-2-(cyclohexylamino)-2-oxo-1-(pyridin-3-yl)ethyl]-1H-imidazole-4-carboxamide), was used as a reference ligand. The docking investigation was accomplished using MOE2014 software. At first, the crystal structure of SARS-Cov-2 main protease was prepared by removing water molecules. Only one chain was retained besides the co-crystallized ligand (GWS). Then, the selected chain was protonated and subjected to minimization of the energy process. Next, the active site of the target protein was defined. Structures of the tested compounds and the co-crystallized ligand were drawn using ChemBioDraw Ultra 14.0 and saved as MDL-SD format. Such file was opened using MOE to display the 3D structures which were protonated and subjected to energy minimization.

Formerly, validation of the docking process was performed by docking the co-crystallized ligand against the isolated pocket of the active site. The produced RMSD value indicated the validity of the process. Finally, docking of the tested compounds was done through the dock option inserted in compute window. For each docked molecule, 30 docked poses were produced using ASE for scoring function and force field for refinement. The results of the docking process were then visualized using Discovery Studio 4.0 software.

8. ADMET

ADMET descriptors (absorption, distribution, metabolism, excretion and toxicity) of the compounds were determined using Discovery studio 4.0. At first, the CHARMM force field was applied then the tested compounds were prepared and minimized according to the preparation of small molecule protocol. Then ADMET descriptors protocol was applied to carry out these studies.

9. Toxicity studies

The toxicity parameters of the tested compounds were calculated using Discovery studio 4.0. Indinavir was used as a reference drug. At first, the CHARMM force field was applied then the compounds were prepared and minimized according to the preparation of small molecule protocol. Then different parameters were calculated from the toxicity prediction (extensible) protocol.

10. Molecular dynamics simulations

The system was prepared using the web-based CHARMM-GUI[1-3] interface with the CHARMM36 force field[4]. All the simulations were done using the NAMD 2.13[5] package. The TIP3P explicit solvation model was used[6], and the periodic boundary conditions were set with a dimension of the dimensions 82.65 Å, 82.36 Å, and 82.64 Å in x, y, and z respectively. The parameters for the top docking results were generated using the CHARMM general force field[7] Afterward, the system was neutralized using 0.15 mol/L (Cl^-/Na^+) ions. The MD protocols involved minimization, equilibration, and production.a 2 *fs* time step of integration was chosen for all MD simulations, the equilibration was carried in the canonical (*NVT*) ensemble, while the isothermal–isobaric (*NPT*) ensemble was for the production. Through the 100 *ns* of MD production, the pressure was set at 1 atm using the Nose´–Hoover Langevin piston barostat[8,9] with a Langevin piston decay of 0.05*ps* and a period of 0.1*ps*. The temperature was set at 298.15 K using the Langevin thermostat[10]. A distance cutoff of 12.0 Å was applied to short-range nonbonded interactions with a pair list distance of 16 Å, and Lennard Jones interactions were

smoothly truncated at 8.0 Å. Long-range electrostatic interactions were treated using the particle-mesh Ewald (PME) method[11,12], where a grid spacing of 1.0 Å was used for all simulation cells. All covalent bonds involving hydrogen atoms were constrained using the SHAKE algorithm[13]. For consistency, we have applied the same protocol for all MD simulations.

Binding Energy Calculations

The one-average molecular mechanics generalized Born surface area (MM/GBSA)[14,15]approach implemented in the MOLAICAL code[16] was used for the relative binding energy calculations, in which the ligand (L) binds to the protein receptor (R) to form the complex (RL),

$$\Delta G_{bind} = \Delta G_{RL} - \Delta G_{R} - \Delta G_{L}$$

which can be represented by contributions of different interactions,

$$\Delta G_{bind} = \Delta H - T\Delta S = \Delta E_{MM} + \Delta G_{Sol} - T\Delta S$$

where the changes in the gas phase molecular mechanics (ΔE_{MM}), solvation Gibbs energy (ΔG_{Sol}), and conformational entropy $(-T\Delta S)$ are determined as follows: ΔE_{MM} is the sum of the changes in the electrostatic energies ΔE_{ele}, the van der Waals energies ΔE_{vdW}, and the internal energies ΔE_{int} (bonded interactions); ΔG_{Sol} is the total of both the polar solvation (calculated using the generalized Born model) and the nonpolar solvation (calculated using the solvent-accessible surface area) and $-T\Delta S$ is calculated by the normal mode analysis. The solvent dielectric constant of 78.5 and the surface tension constant of 0.03012 kJ mol^{-1} Å^{2} were used for MM/GBSA calculations.

1. Jo, S.; Kim, T.; Iyer, V.G.; Im, W. CHARMM-GUI: A web-based graphical user interface for CHARMM. *Journal of computational chemistry* **2008**, *29*, 1859-1865, doi:https://doi.org/10.1002/jcc.20945.
2. Brooks, B.R.; Brooks III, C.L.; Mackerell Jr., A.D.; Nilsson, L.; Petrella, R.J.; Roux, B.; Won, Y.; Archontis, G.; Bartels, C.; Boresch, S., et al. CHARMM: The biomolecular simulation program. *Journal of computational chemistry* **2009**, *30*, 1545-1614, doi:https://doi.org/10.1002/jcc.21287.
3. Lee, J.; Cheng, X.; Swails, J.M.; Yeom, M.S.; Eastman, P.K.; Lemkul, J.A.; Wei, S.; Buckner, J.; Jeong, J.C.; Qi, Y., et al. CHARMM-GUI Input Generator for NAMD, GROMACS, AMBER, OpenMM, and CHARMM/OpenMM Simulations Using the CHARMM36 Additive Force Field. *Journal of chemical theory and computation* **2016**, *12*, 405-413, doi:10.1021/acs.jctc.5b00935.
4. Best, R.B.; Zhu, X.; Shim, J.; Lopes, P.E.; Mittal, J.; Feig, M.; Mackerell, A.D., Jr. Optimization of the additive CHARMM all-atom protein force field targeting improved sampling of the backbone phi, psi and side-chain chi(1) and chi(2) dihedral angles. *Journal of chemical theory and computation* **2012**, *8*, 3257-3273, doi:10.1021/ct300400x.

5. Phillips, J.C.; Braun, R.; Wang, W.; Gumbart, J.; Tajkhorshid, E.; Villa, E.; Chipot, C.; Skeel, R.D.; Kale, L.; Schulten, K. Scalable molecular dynamics with NAMD. *Journal of computational chemistry* **2005**, *26*, 1781-1802, doi:10.1002/jcc.20289.
6. Jorgensen, W.L.; Chandrasekhar, J.; Madura, J.D.; Impey, R.W.; Klein, M.L. Comparison of simple potential functions for simulating liquid water. *The Journal of Chemical Physics* **1983**, *79*, 926-935, doi:10.1063/1.445869.
7. Yu, W.; He, X.; Vanommeslaeghe, K.; MacKerell, A.D., Jr. Extension of the CHARMM General Force Field to sulfonyl-containing compounds and its utility in biomolecular simulations. *Journal of computational chemistry* **2012**, *33*, 2451-2468, doi:10.1002/jcc.23067.
8. Nosé, S.; Klein, M.L. Constant pressure molecular dynamics for molecular systems. *Molecular Physics* **1983**, *50*, 1055-1076, doi:10.1080/00268978300102851.
9. Nosé, S. A molecular dynamics method for simulations in the canonical ensemble. *Molecular Physics* **1984**, *52*, 255-268, doi:10.1080/00268978400101201.
10. Grest, G.S.; Kremer, K. Molecular dynamics simulation for polymers in the presence of a heat bath. *Physical review. A, General physics* **1986**, *33*, 3628-3631, doi:10.1103/physreva.33.3628.
11. Darden, T.; York, D.; Pedersen, L. Particle mesh Ewald: AnN·log(N) method for Ewald sums in large systems. *The Journal of Chemical Physics* **1993**, *98*, 10089-10092, doi:10.1063/1.464397.
12. Essmann, U.; Perera, L.; Berkowitz, M.L.; Darden, T.; Lee, H.; Pedersen, L.G. A smooth particle mesh Ewald method. *The Journal of Chemical Physics* **1995**, *103*, 8577-8593, doi:10.1063/1.470117.
13. Ryckaert, J.-P.; Ciccotti, G.; Berendsen, H.J.C. Numerical integration of the cartesian equations of motion of a system with constraints: molecular dynamics of n-alkanes. *Journal of Computational Physics* **1977**, *23*, 327-341, doi:https://doi.org/10.1016/0021-9991(77)90098-5.
14. Genheden, S.; Ryde, U. Comparison of end-point continuum-solvation methods for the calculation of protein-ligand binding free energies. *Proteins* **2012**, *80*, 1326-1342, doi:10.1002/prot.24029.
15. Wang, E.; Sun, H.; Wang, J.; Wang, Z.; Liu, H.; Zhang, J.Z.H.; Hou, T. End-Point Binding Free Energy Calculation with MM/PBSA and MM/GBSA: Strategies and Applications in Drug Design. *Chemical reviews* **2019**, *119*, 9478-9508, doi:10.1021/acs.chemrev.9b00055.
16. Bai, Q.; Tan, S.; Xu, T.; Liu, H.; Huang, J.; Yao, X. MolAICal: a soft tool for 3D drug design of protein targets by artificial intelligence and classical algorithm. *Briefings in bioinformatics* **2021**, *22*, bbaa161.

Table S.1 - ^{1}H and ^{13}C spectral data **2** ($CDCl_3$, δ)

Position	δH (J=Hz)	δ C	Position	δH (J=Hz)	δ C
2	-	180	9	-	162.21
3	7.63 d (9.52)	143.72	10	-	155.93
4	6.24 (9.91)	112.53	11	4.58 (6.79)	65.49
5	7.36 d (8.61)	139.43	12	5.48 t (6.6, 2.2)	118.71
6	6.85 dd (8.72, 2.3)	112.98	13	-	139.42
7	-	161.42	14	1.82 s	25.89
8	6.82 d (2.3)	101.99	15	1.77 s	18.46

Spectral data

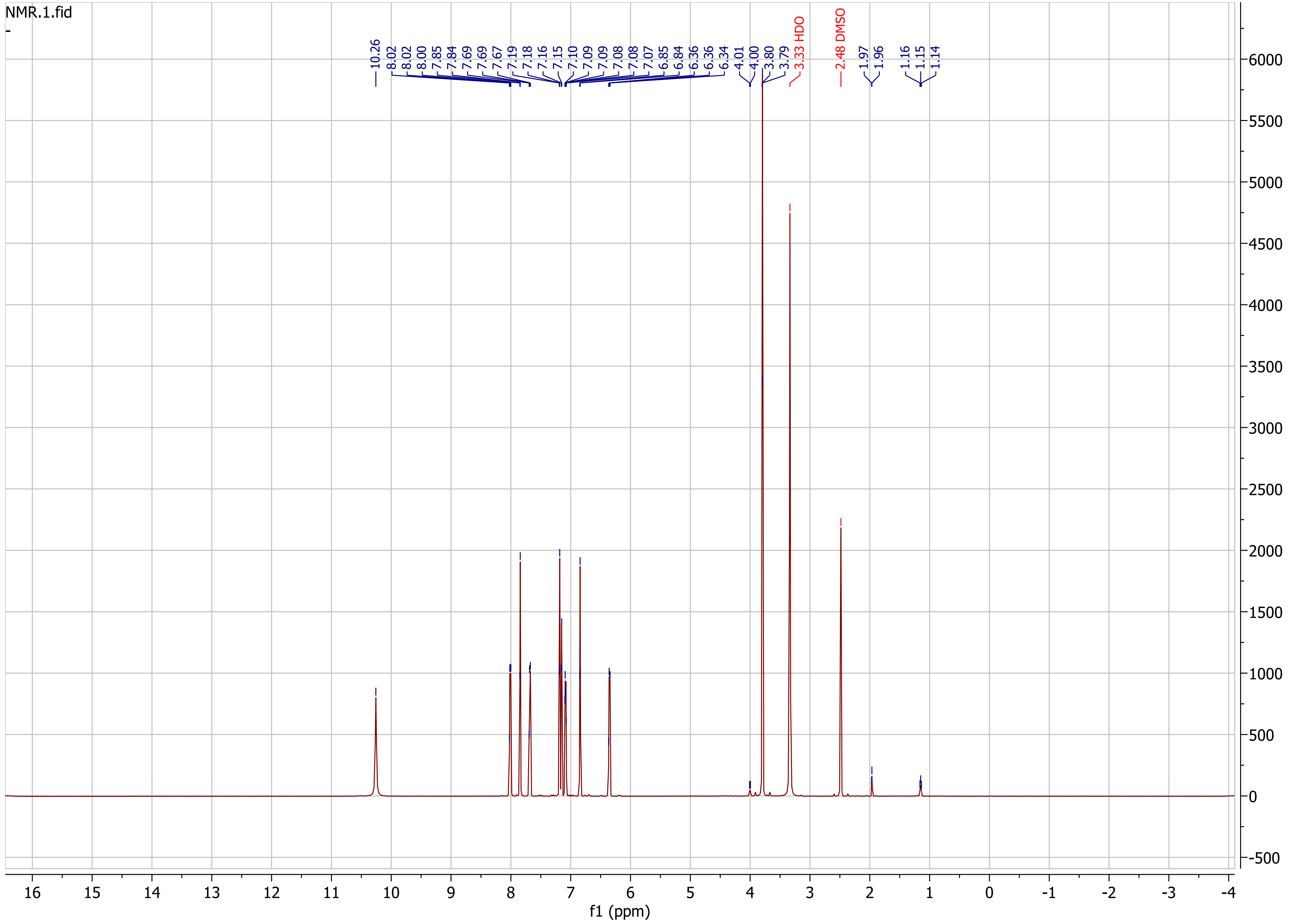

NMR.1.fid
-
10.26
8.02
8.02
8.00
7.85
7.84
7.69
7.69
7.67
7.19
7.18
7.16
7.15
7.10
7.09
7.09
7.08
7.08
7.07
6.85
6.84
6.36
6.36
6.34
4.01
4.00
3.80
3.79
3.33 HDO
2.48 DMSO
1.97
1.96
1.16
1.15
1.14
f1 (ppm)

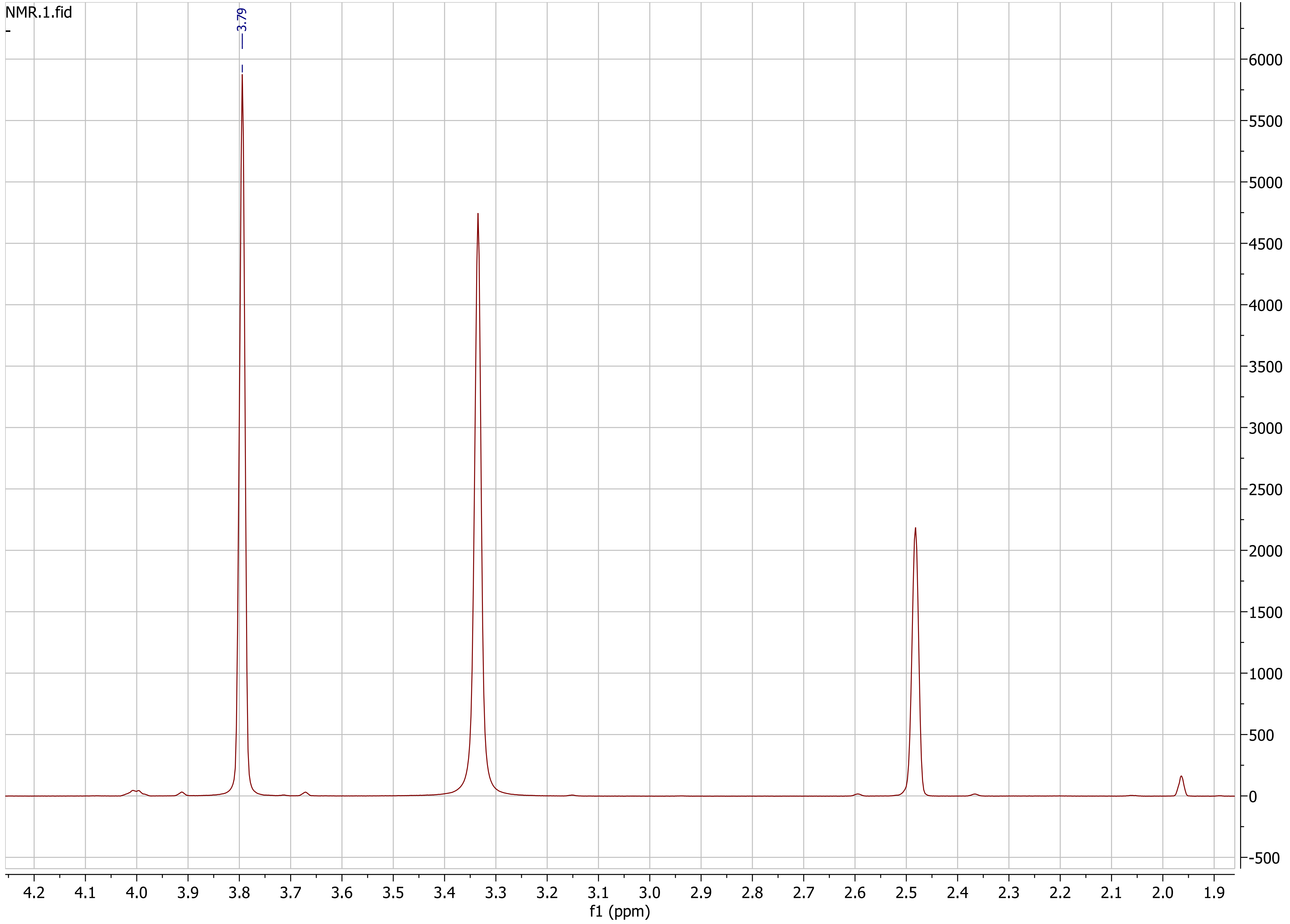
NMR.1.fid
-
3.79
6000
5500
5000
4500
4000
3500
3000
2500
2000
1500
1000
500
0
-500
4.2
4.1
4.0
3.9
3.8
3.7
3.6
3.5
3.4
3.3
3.2
3.1
3.0
2.9
2.8
2.7
2.6
2.5
2.4
2.3
2.2
2.1
2.0
1.9
f1 (ppm)

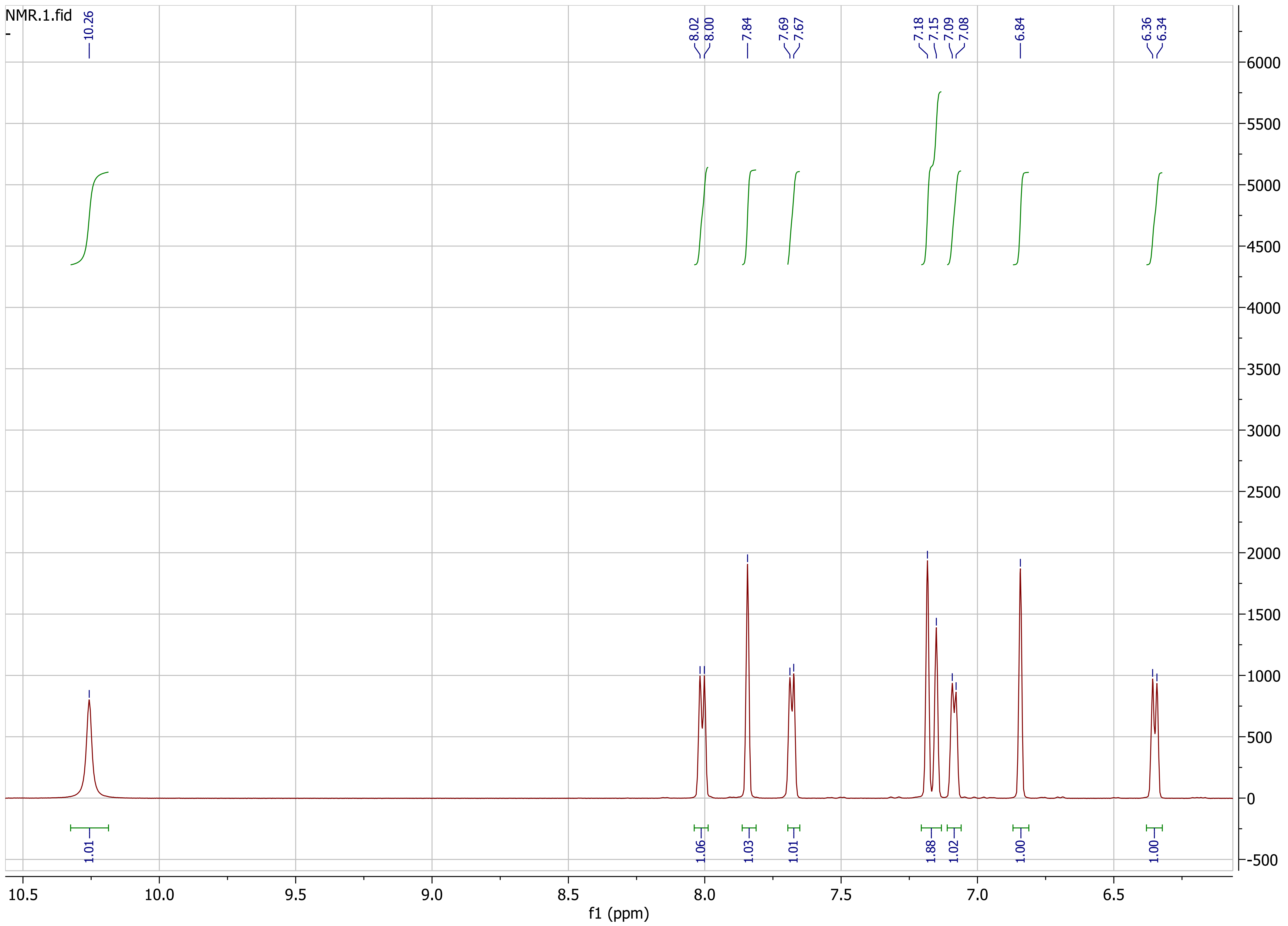

NMR.1.fid
10.26
8.02
8.00
7.84
7.69
7.67
7.18
7.15
7.09
7.08
6.84
6.36
6.34
1.01
1.06
1.03
1.01
1.88
1.02
1.00
1.00
f1 (ppm)

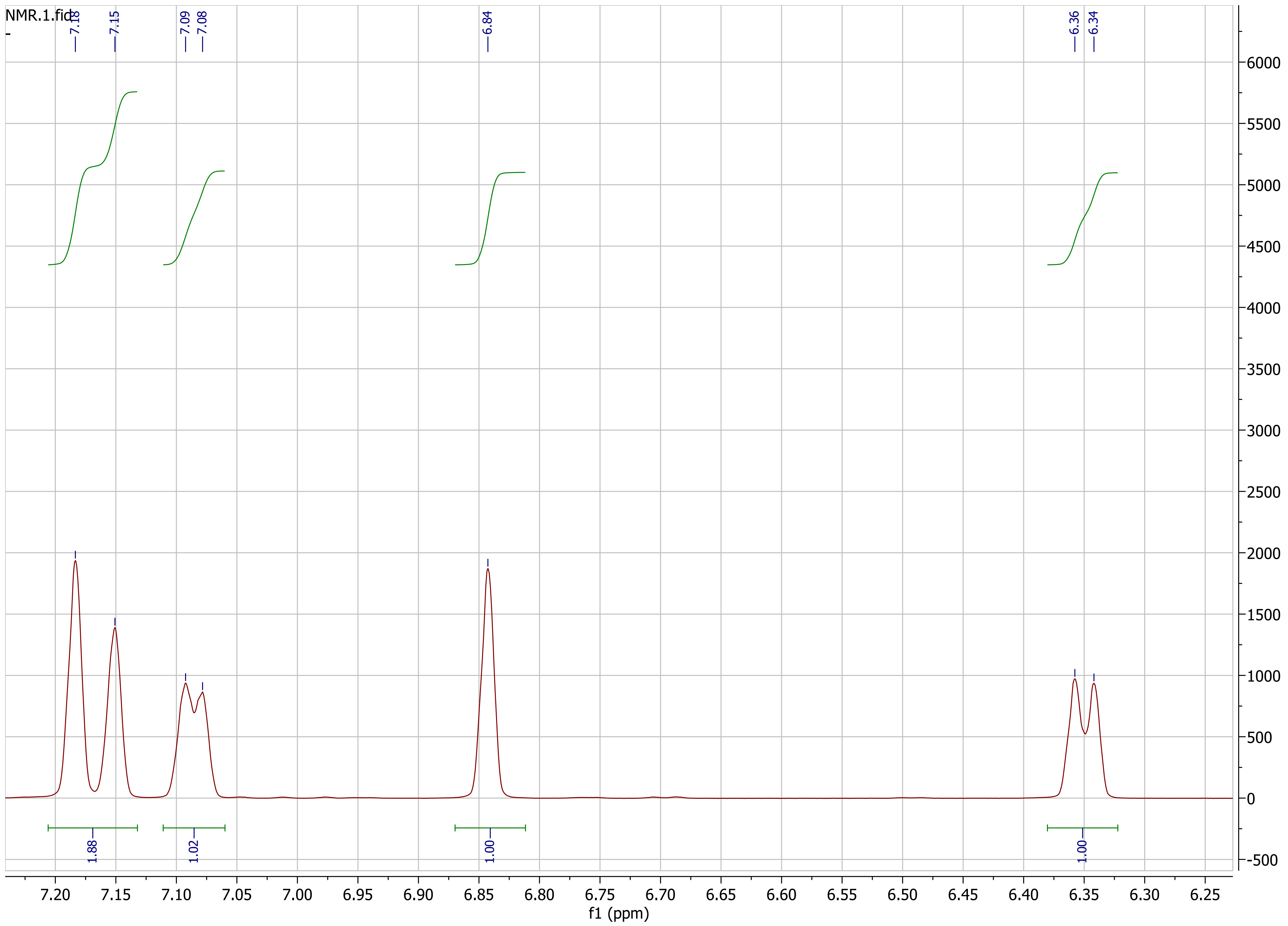
NMR.1.fid
7.18
7.15
7.09
7.08
6.84
6.36
6.34
1.88
1.02
1.00
1.00
f1 (ppm)

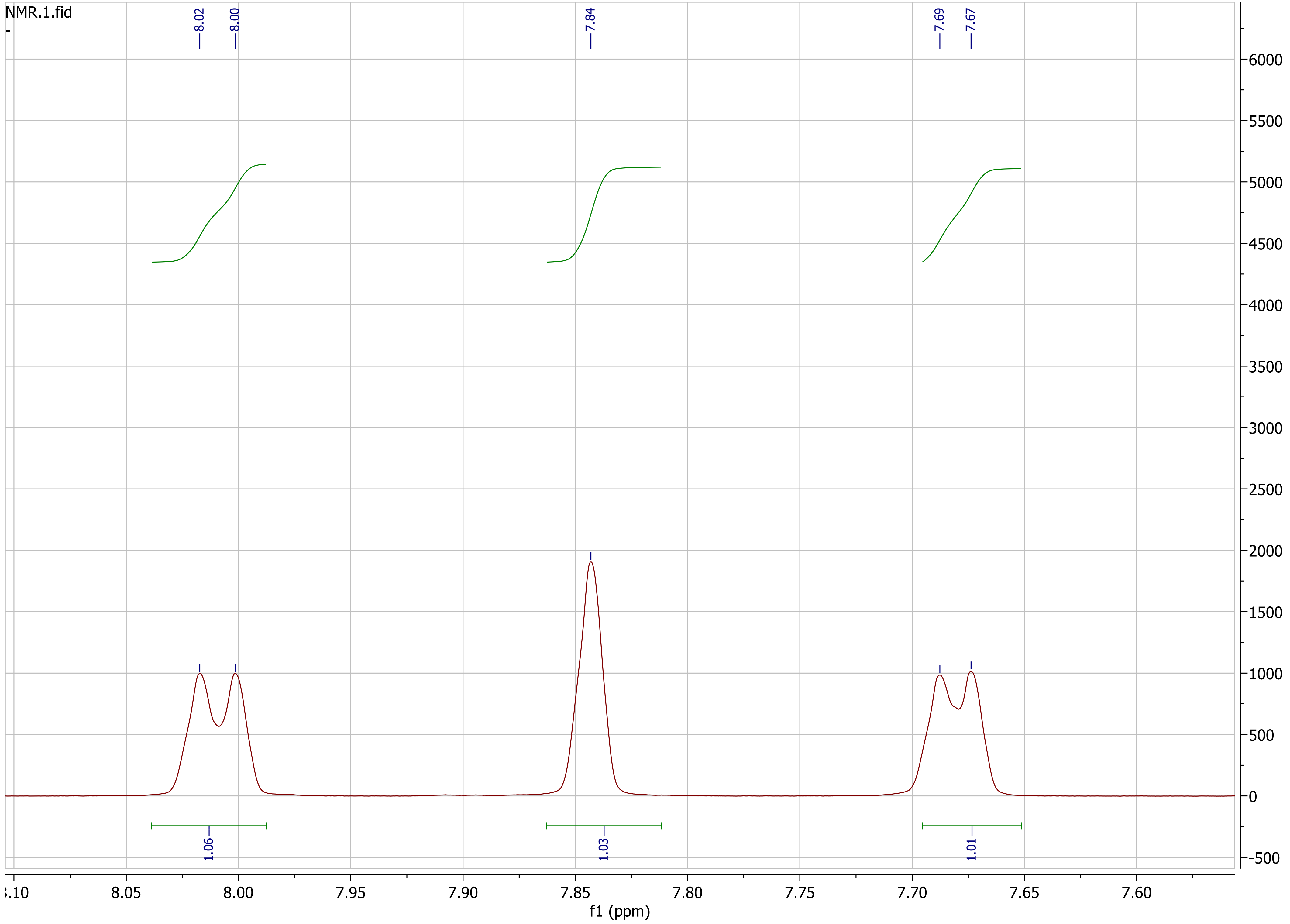

NMR.1.fid
8.02
8.00
7.84
7.69
7.67
1.06
1.03
1.01
f1 (ppm)

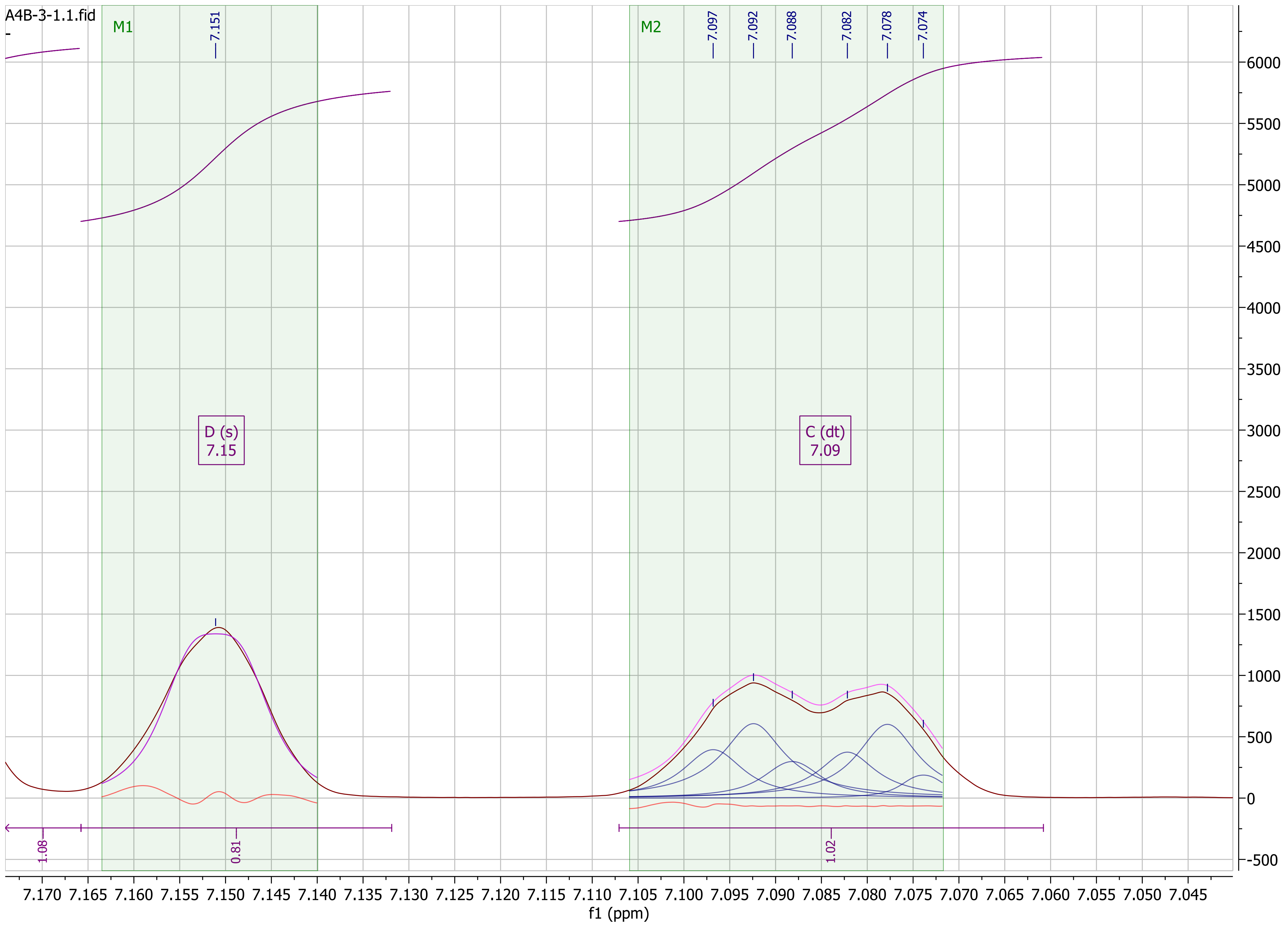

A4B-3-1.1.fid
M1
M2
7.151
7.097
7.092
7.088
7.082
7.078
7.074
D (s)
7.15
C (dt)
7.09
1.08
0.81
1.02
6000
5500
5000
4500
4000
3500
3000
2500
2000
1500
1000
500
0
-500
7.170 7.165 7.160 7.155 7.150 7.145 7.140 7.135 7.130 7.125 7.120 7.115 7.110 7.105 7.100 7.095 7.090 7.085 7.080 7.075 7.070 7.065 7.060 7.055 7.050 7.045
f1 (ppm)

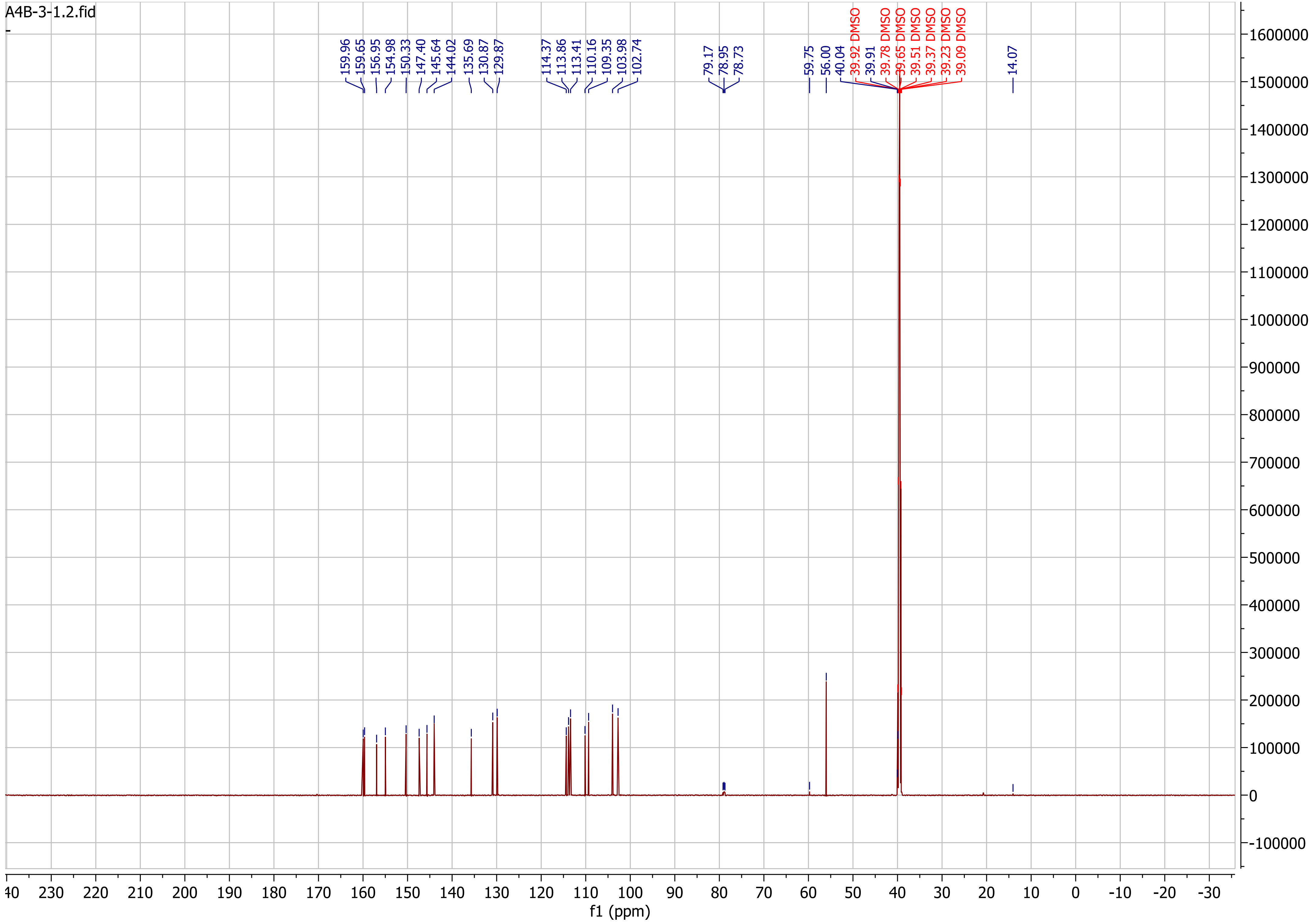

A4B-3-1.2.fid
159.96
159.65
156.95
154.98
150.33
147.40
145.64
144.02
135.69
130.87
129.87
114.37
113.86
113.41
110.16
109.35
103.98
102.74
79.17
78.95
78.73
59.75
56.00
40.04
39.92 DMSO
39.91
39.78 DMSO
39.65 DMSO
39.51 DMSO
39.37 DMSO
39.23 DMSO
39.09 DMSO
14.07
f1 (ppm)

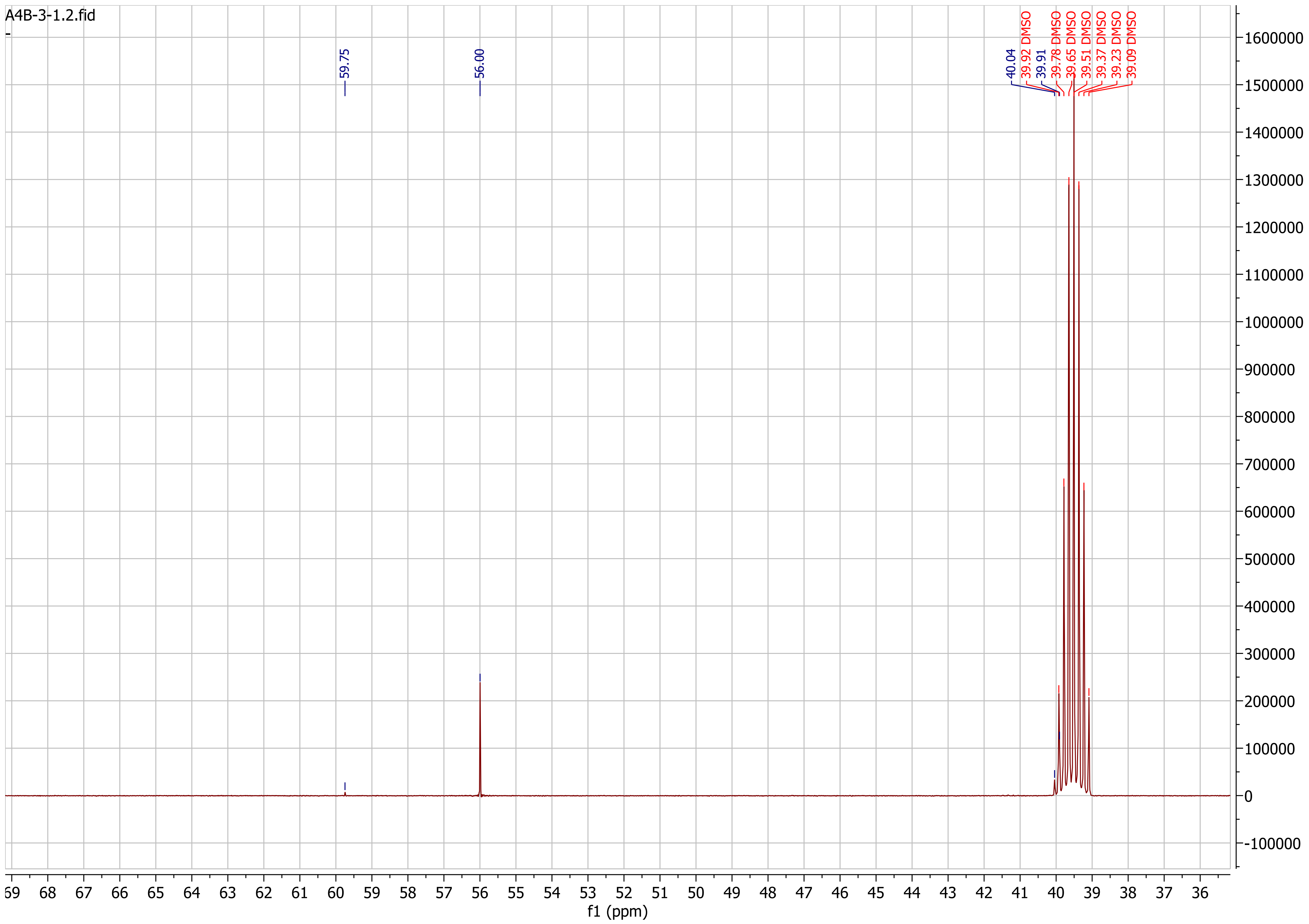
A4B-3-1.2.fid
59.75
56.00
40.04
39.92 DMSO
39.91
39.78 DMSO
39.65 DMSO
39.51 DMSO
39.37 DMSO
39.23 DMSO
39.09 DMSO
f1 (ppm)

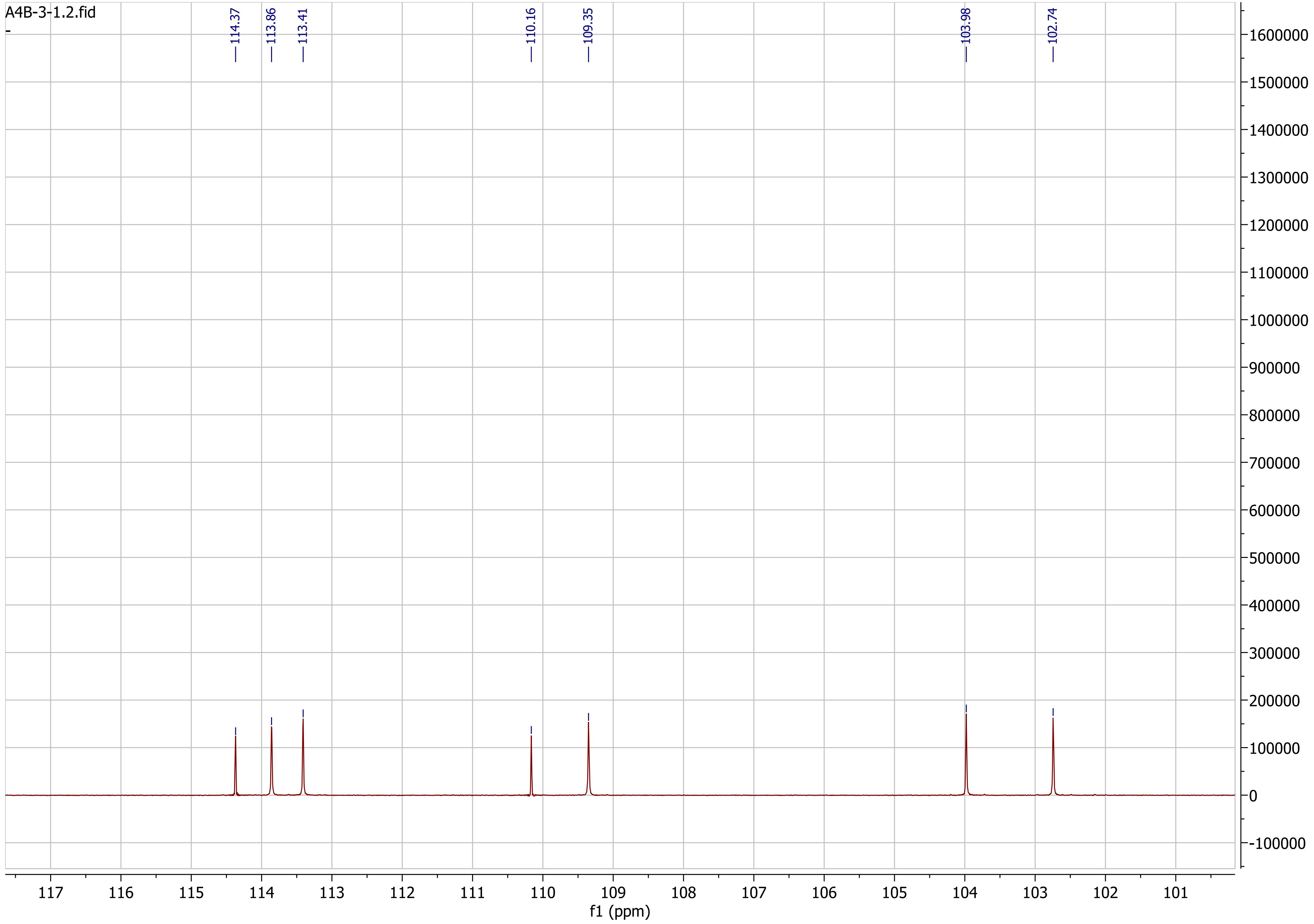
A4B-3-1.2.fid
-
114.37
113.86
113.41
110.16
109.35
103.98
102.74
f1 (ppm)

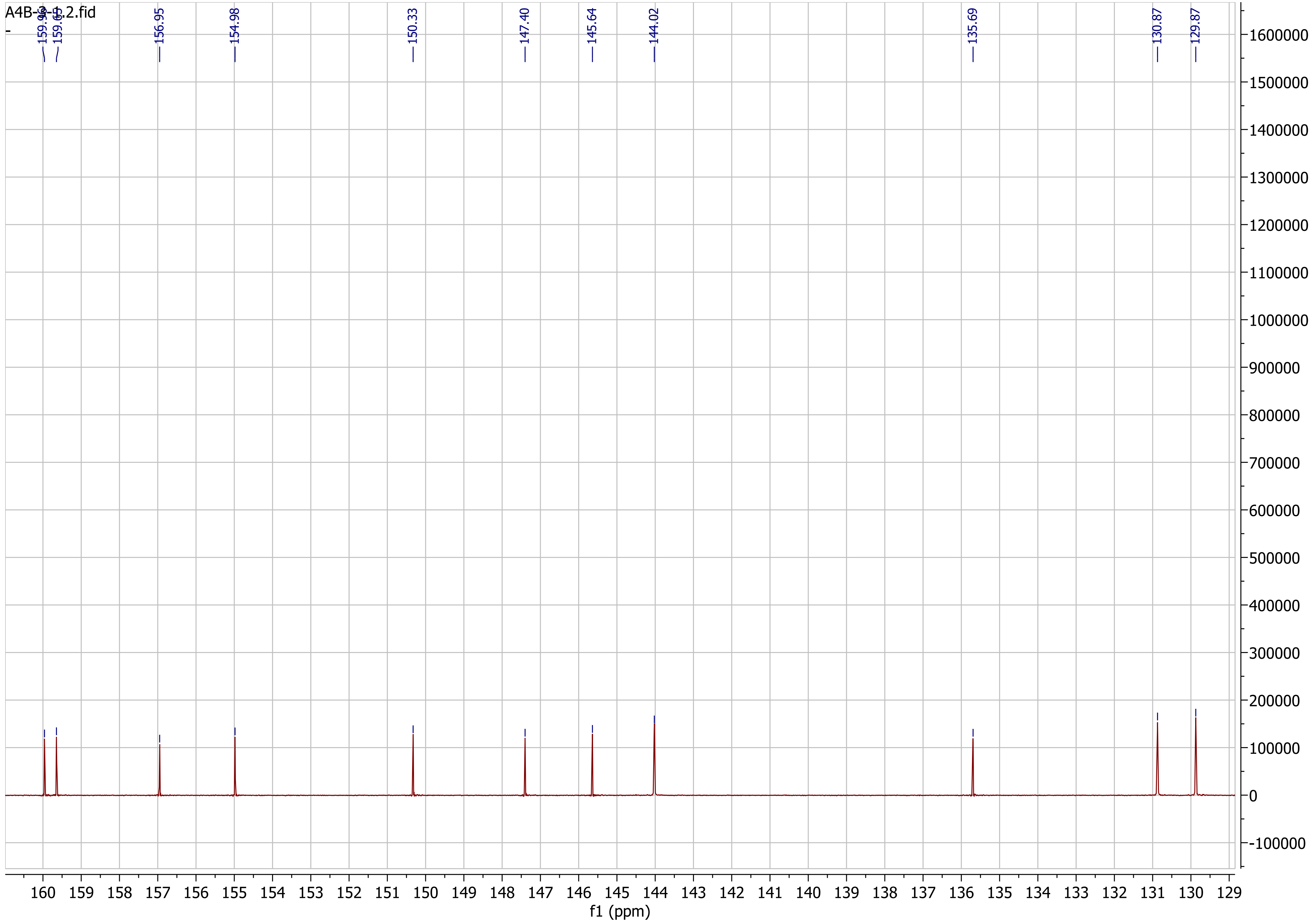
A4B-3-1.2.fid
159.96
159.65
156.95
154.98
150.33
147.40
145.64
144.02
135.69
130.87
129.87
1600000
1500000
1400000
1300000
1200000
1100000
1000000
900000
800000
700000
600000
500000
400000
300000
200000
100000
0
-100000
160 159 158 157 156 155 154 153 152 151 150 149 148 147 146 145 144 143 142 141 140 139 138 137 136 135 134 133 132 131 130 129
f1 (ppm)

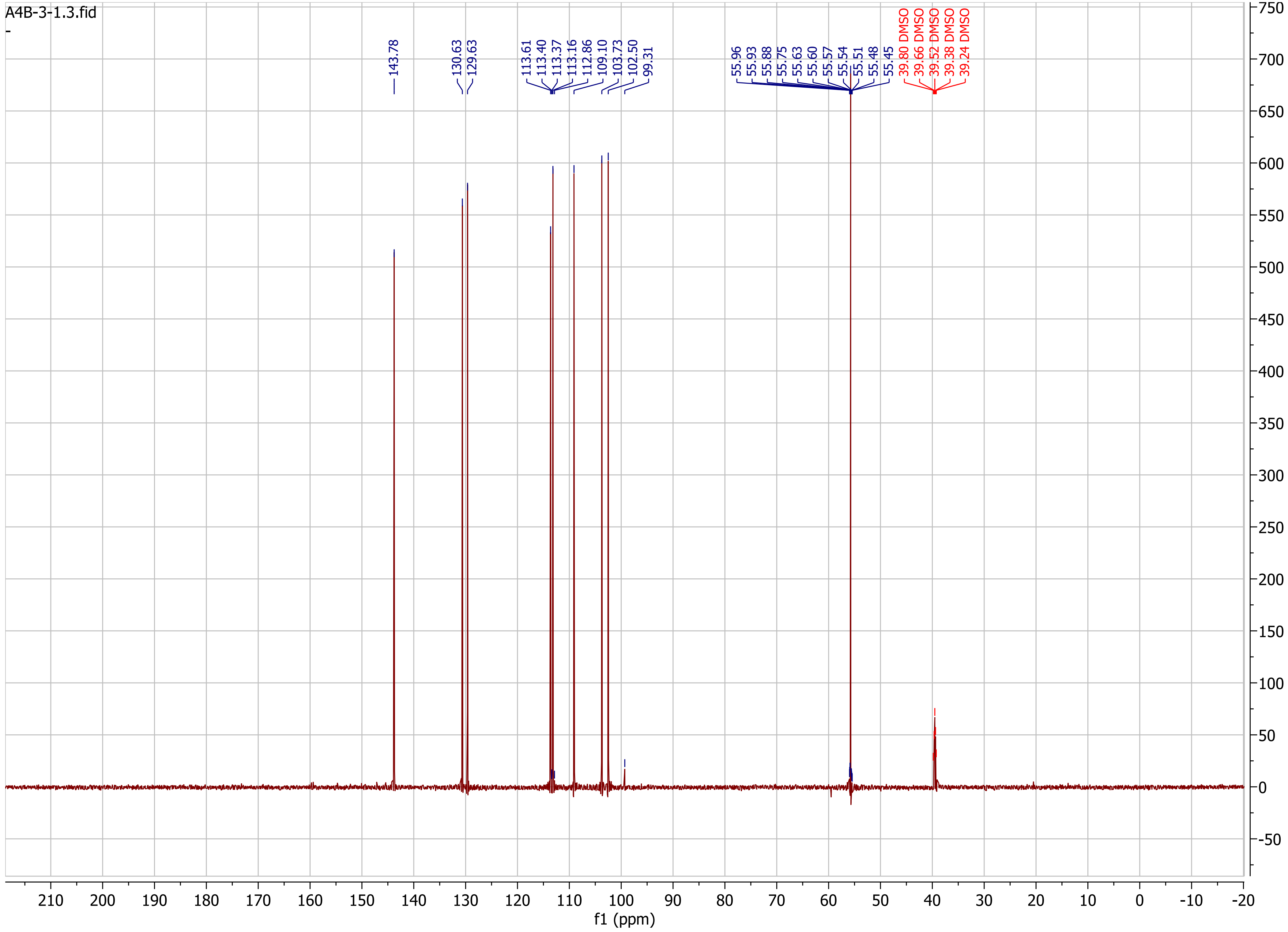

A4B-3-1.3.fid
143.78
130.63
129.63
113.61
113.40
113.37
113.16
112.86
109.10
103.73
102.50
99.31
55.96
55.93
55.88
55.75
55.63
55.60
55.57
55.54
55.51
55.48
55.45
39.80 DMSO
39.66 DMSO
39.52 DMSO
39.38 DMSO
39.24 DMSO
f1 (ppm)

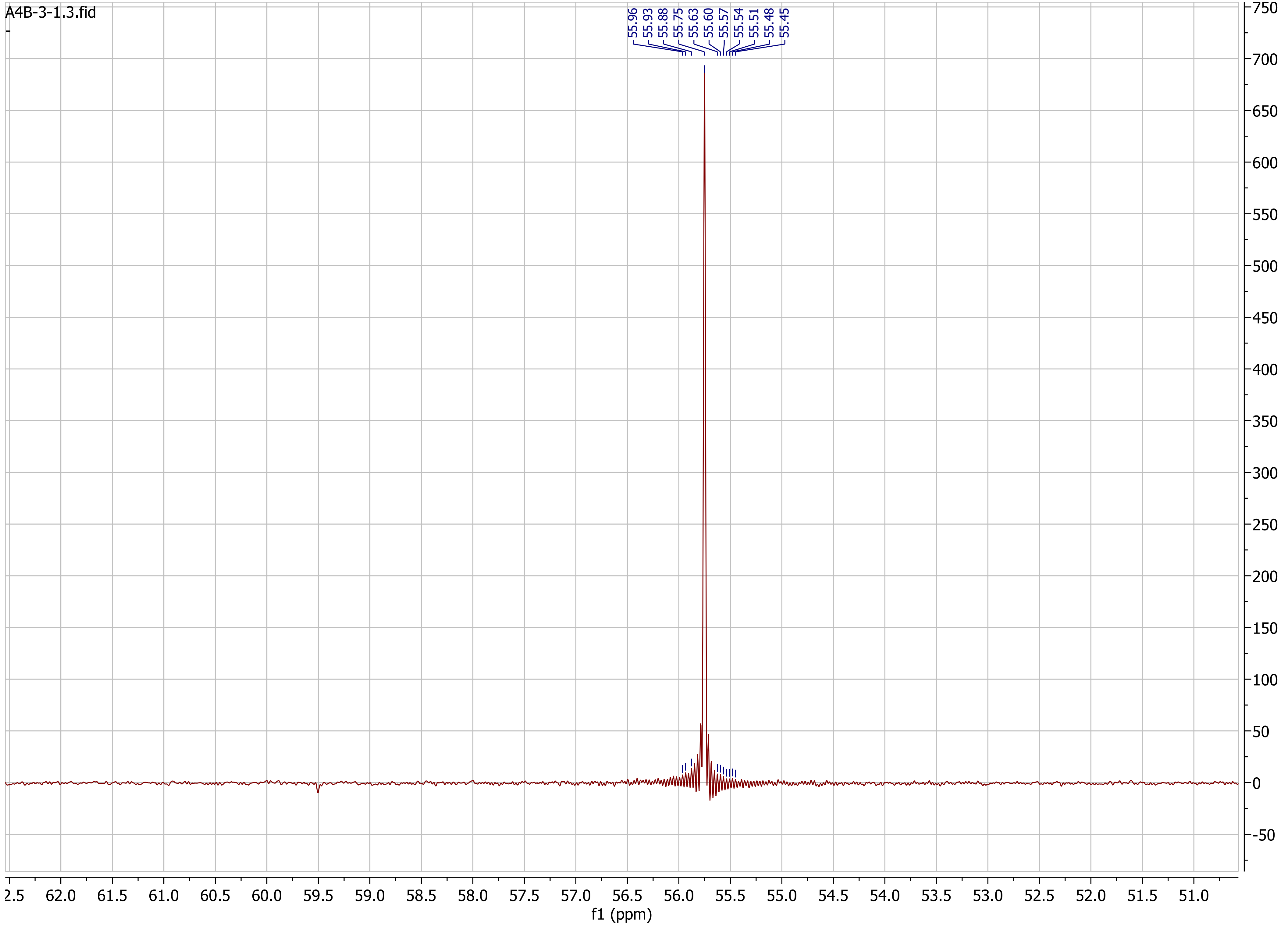

A4B-3-1.3.fid
55.96
55.93
55.88
55.75
55.63
55.60
55.57
55.54
55.51
55.48
55.45
750
700
650
600
550
500
450
400
350
300
250
200
150
100
50
0
-50
2.5
62.0
61.5
61.0
60.5
60.0
59.5
59.0
58.5
58.0
57.5
57.0
56.5
56.0
55.5
55.0
54.5
54.0
53.5
53.0
52.5
52.0
51.5
51.0
f1 (ppm)

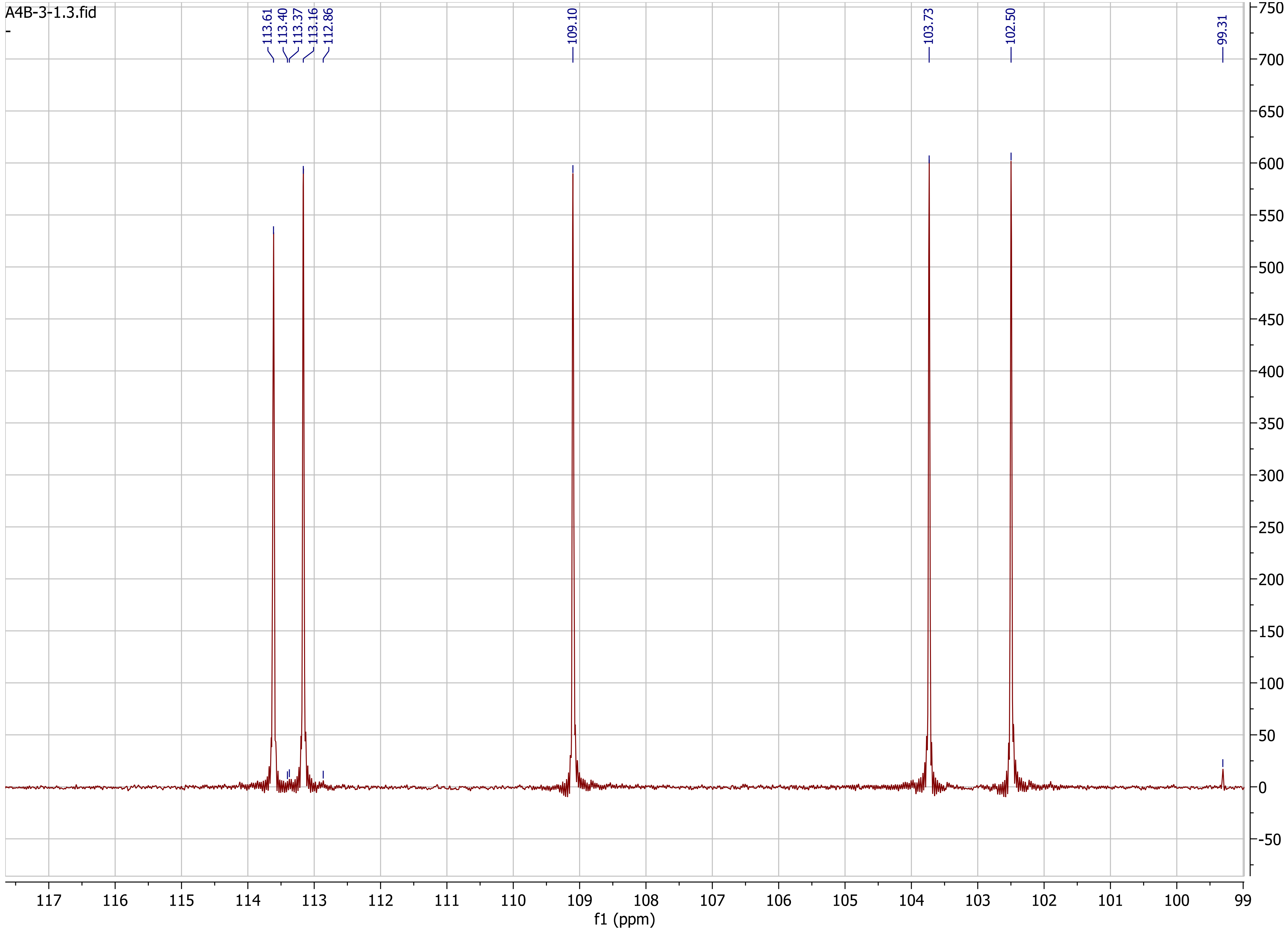

A4B-3-1.3.fid
-
113.61
113.40
113.37
113.16
112.86
109.10
103.73
102.50
99.31
f1 (ppm)

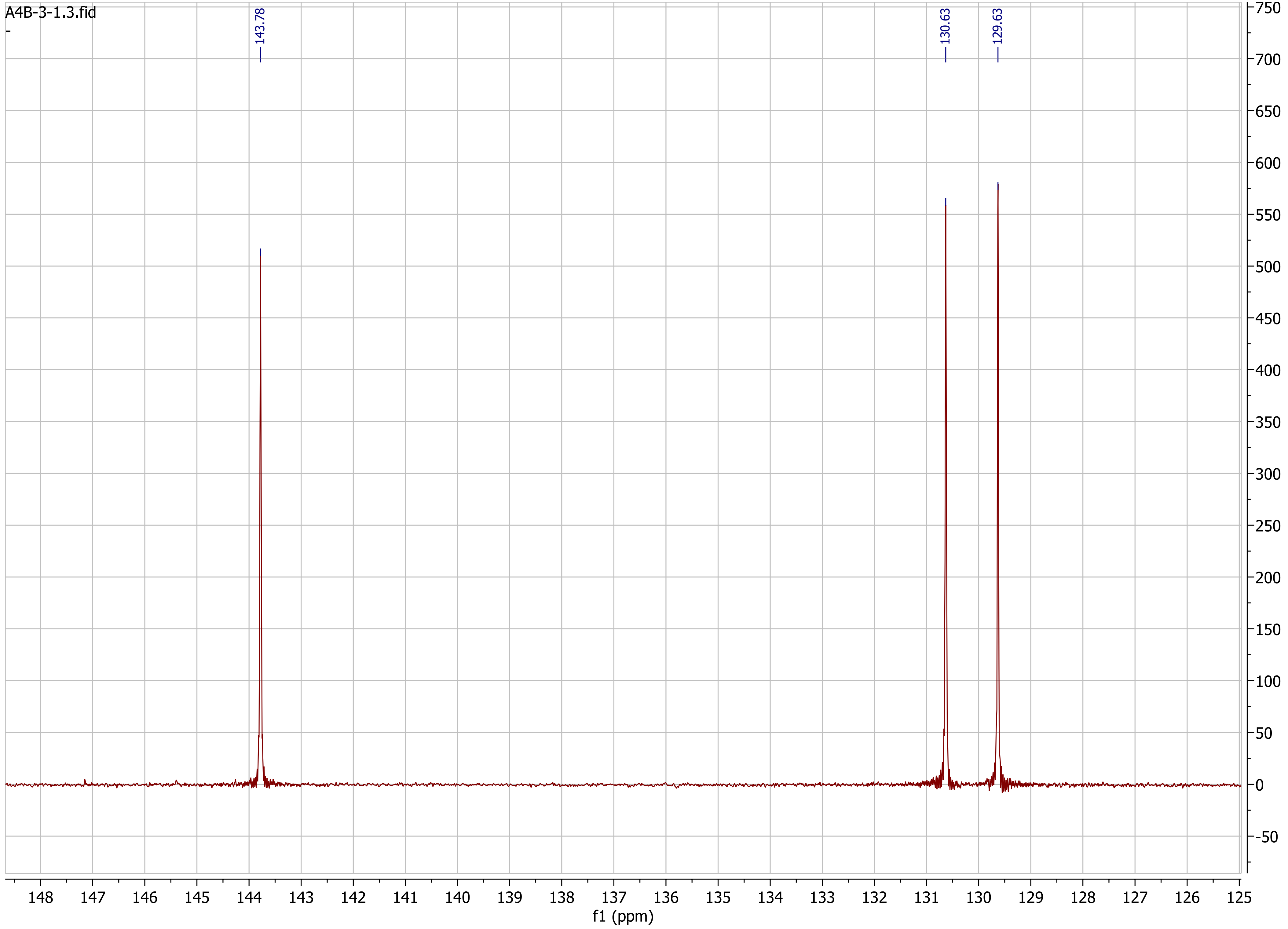
A4B-3-1.3.fid
143.78
130.63
129.63
f1 (ppm)

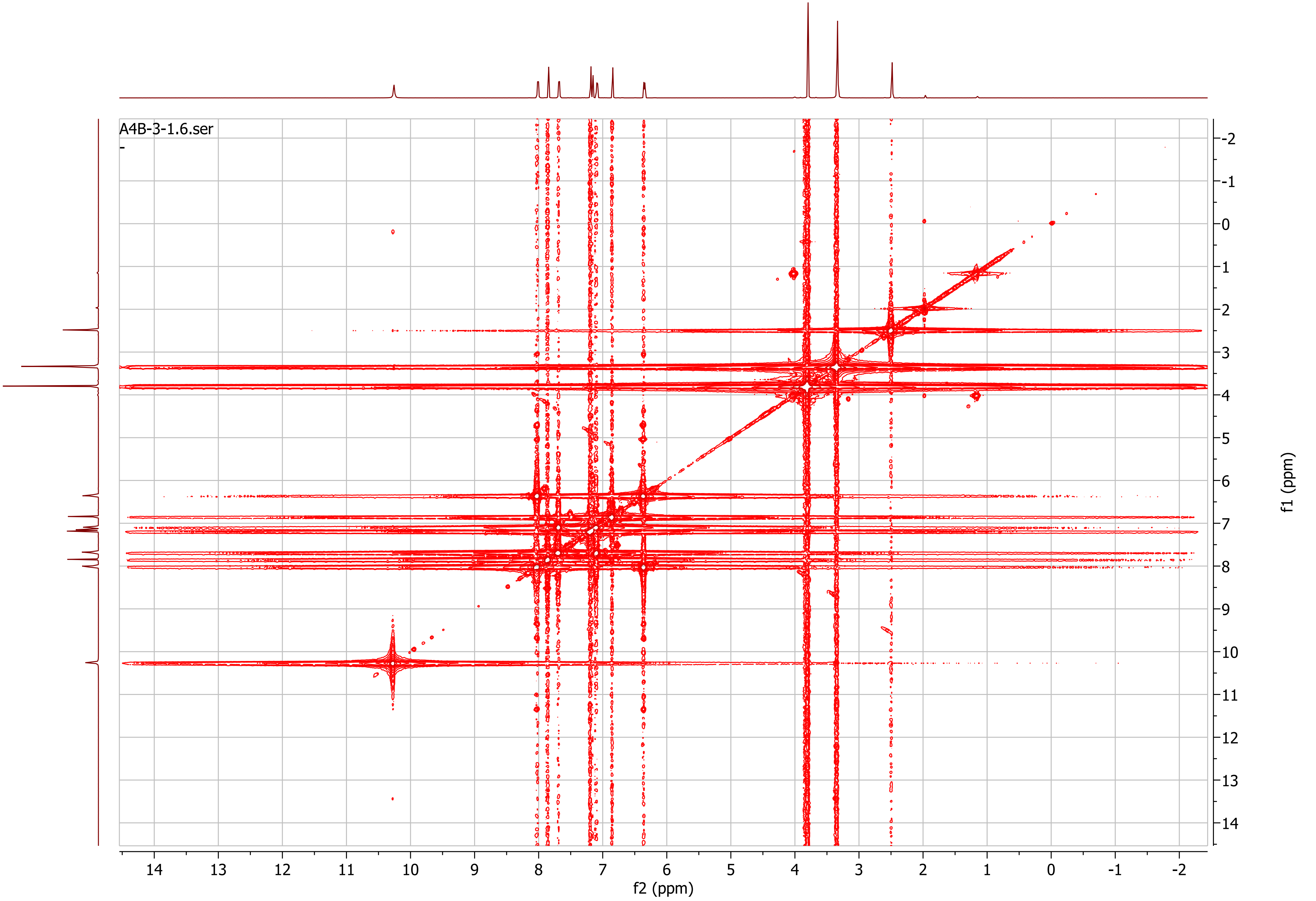

A4B-3-1.6.ser
f2 (ppm)
f1 (ppm)

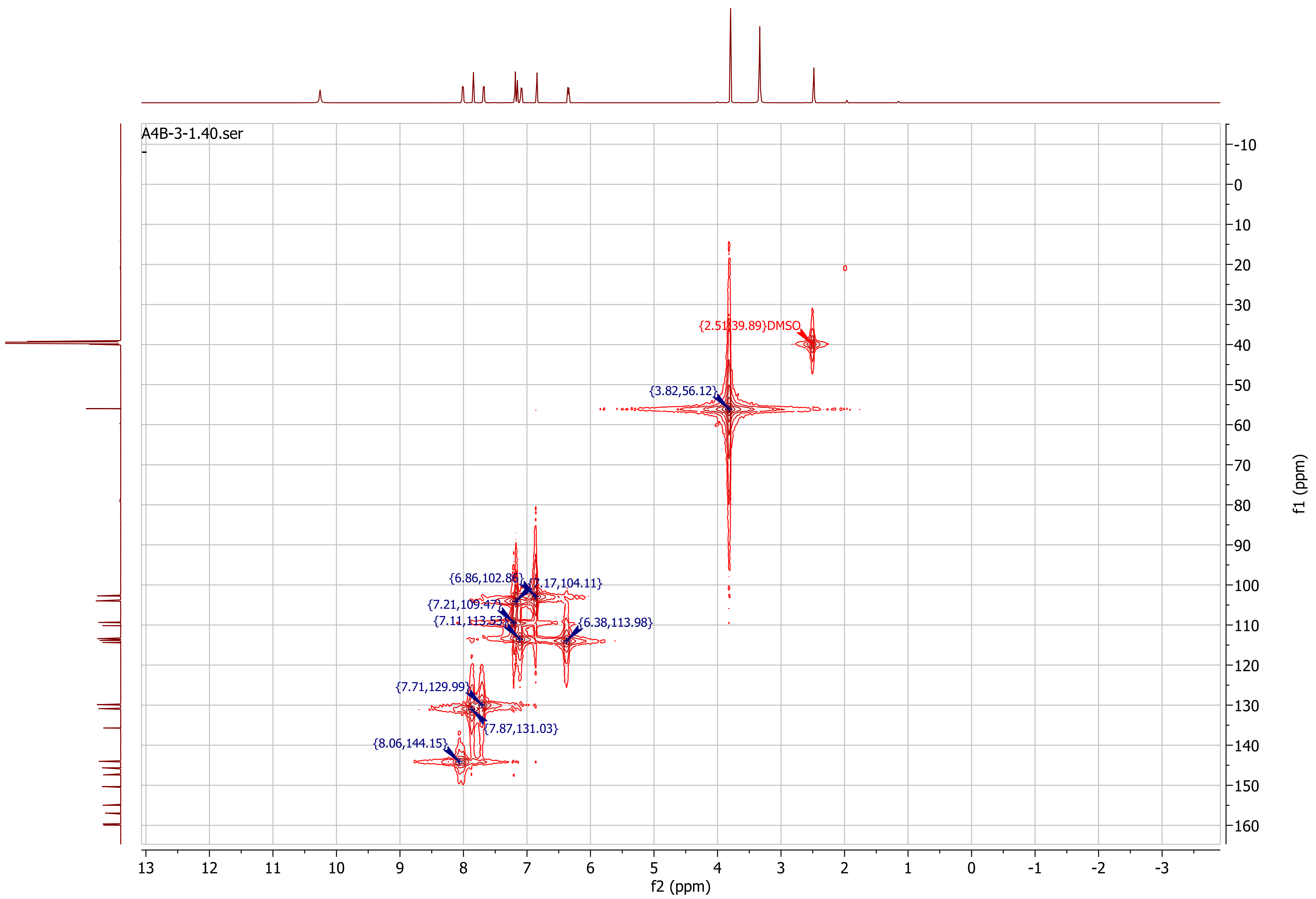

A4B-3-1.40.ser
{2.51,39.89}DMSO
{3.82,56.12}
{6.86,102.86}
{7.17,104.11}
{7.21,109.47}
{7.11,113.53}
{6.38,113.98}
{7.71,129.99}
{7.87,131.03}
{8.06,144.15}
f2 (ppm)
f1 (ppm)

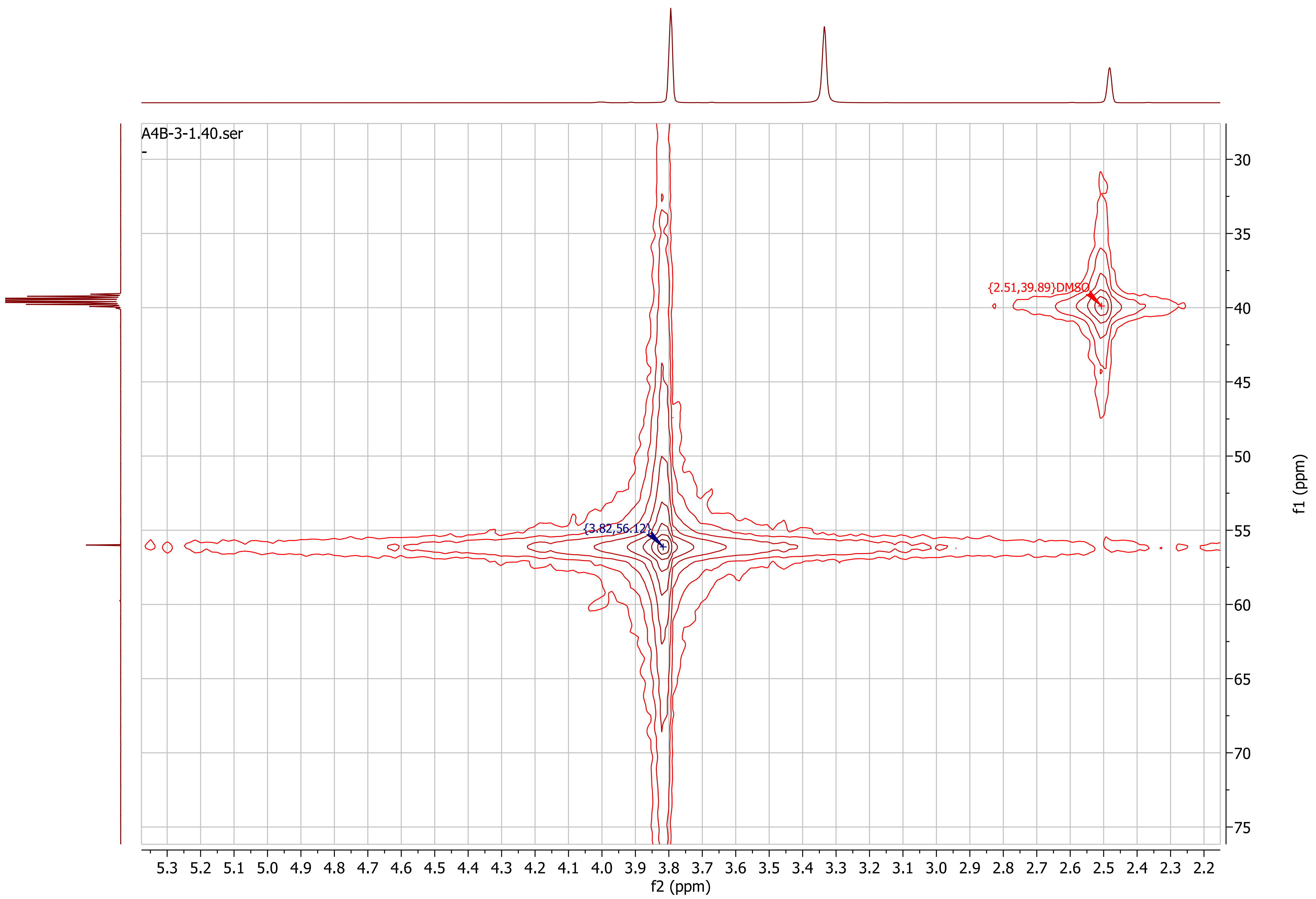

A4B-3-1.40.ser
{3.82,56.12}
{2.51,39.89}DMSO
f2 (ppm)
f1 (ppm)

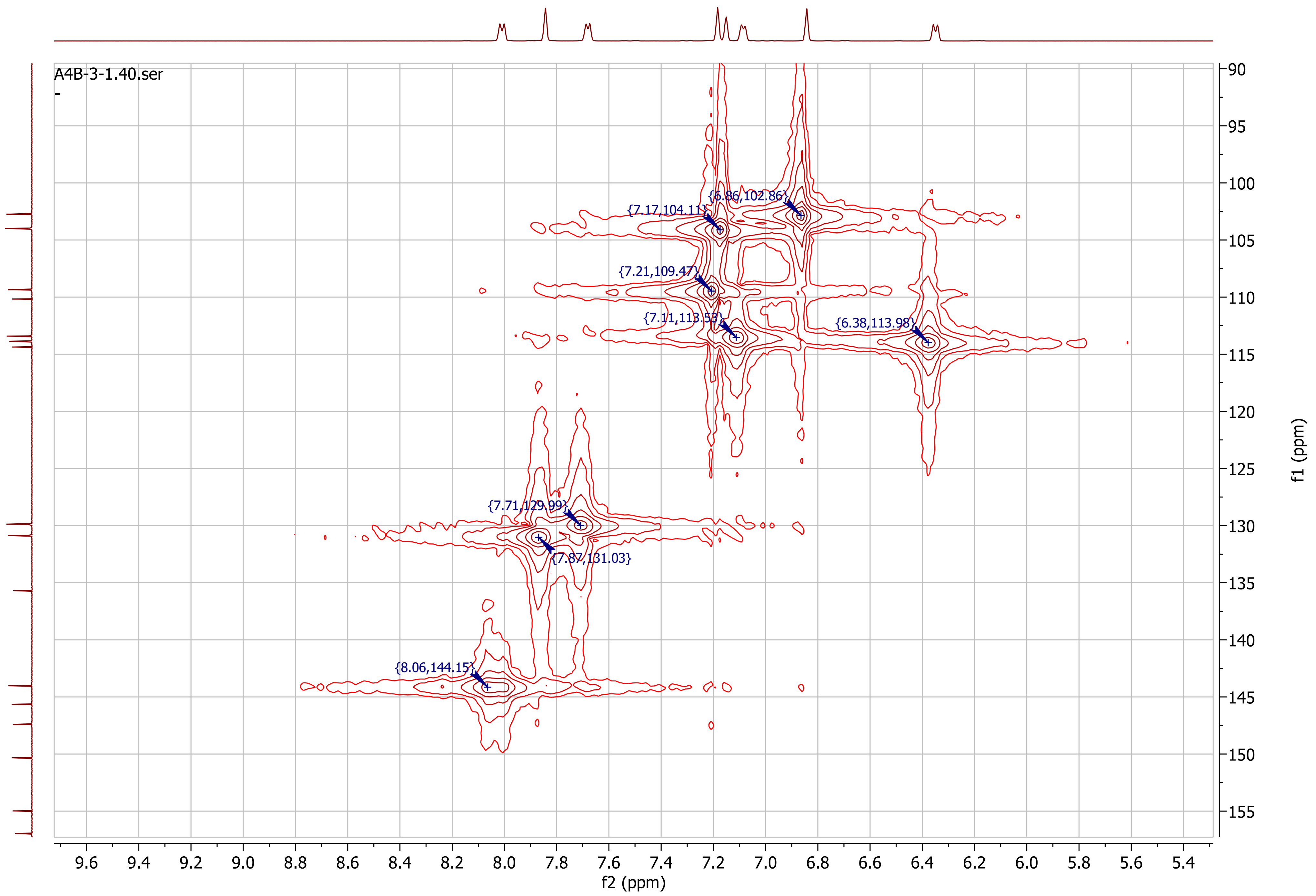

A4B-3-1.40.ser
-
{6.86,102.86}
{7.17,104.11}
{7.21,109.47}
{7.11,113.53}
{6.38,113.98}
{7.71,129.99}
{7.87,131.03}
{8.06,144.15}
f2 (ppm)
f1 (ppm)

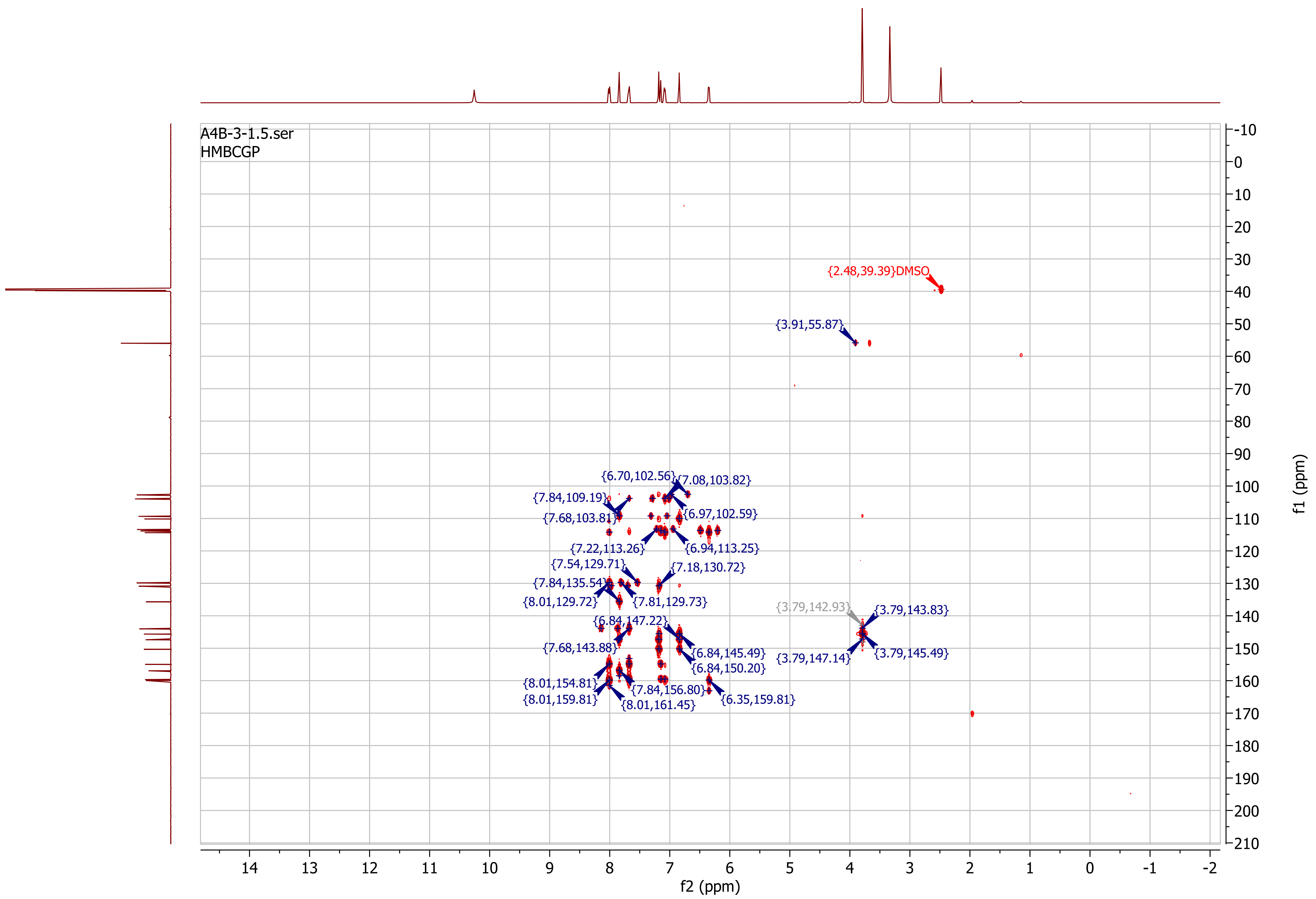
A4B-3-1.5.ser
HMBCGP
{2.48,39.39}DMSO
{3.91,55.87}
{6.70,102.56}
{7.08,103.82}
{7.84,109.19}
{6.97,102.59}
{7.68,103.81}
{7.22,113.26}
{6.94,113.25}
{7.54,129.71}
{7.18,130.72}
{7.84,135.54}
{8.01,129.72}
{7.81,129.73}
{3.79,142.93}
{3.79,143.83}
{6.84,147.22}
{7.68,143.88}
{6.84,145.49}
{3.79,147.14}
{3.79,145.49}
{6.84,150.20}
{8.01,154.81}
{7.84,156.80}
{8.01,159.81}
{8.01,161.45}
{6.35,159.81}
f2 (ppm)
f1 (ppm)

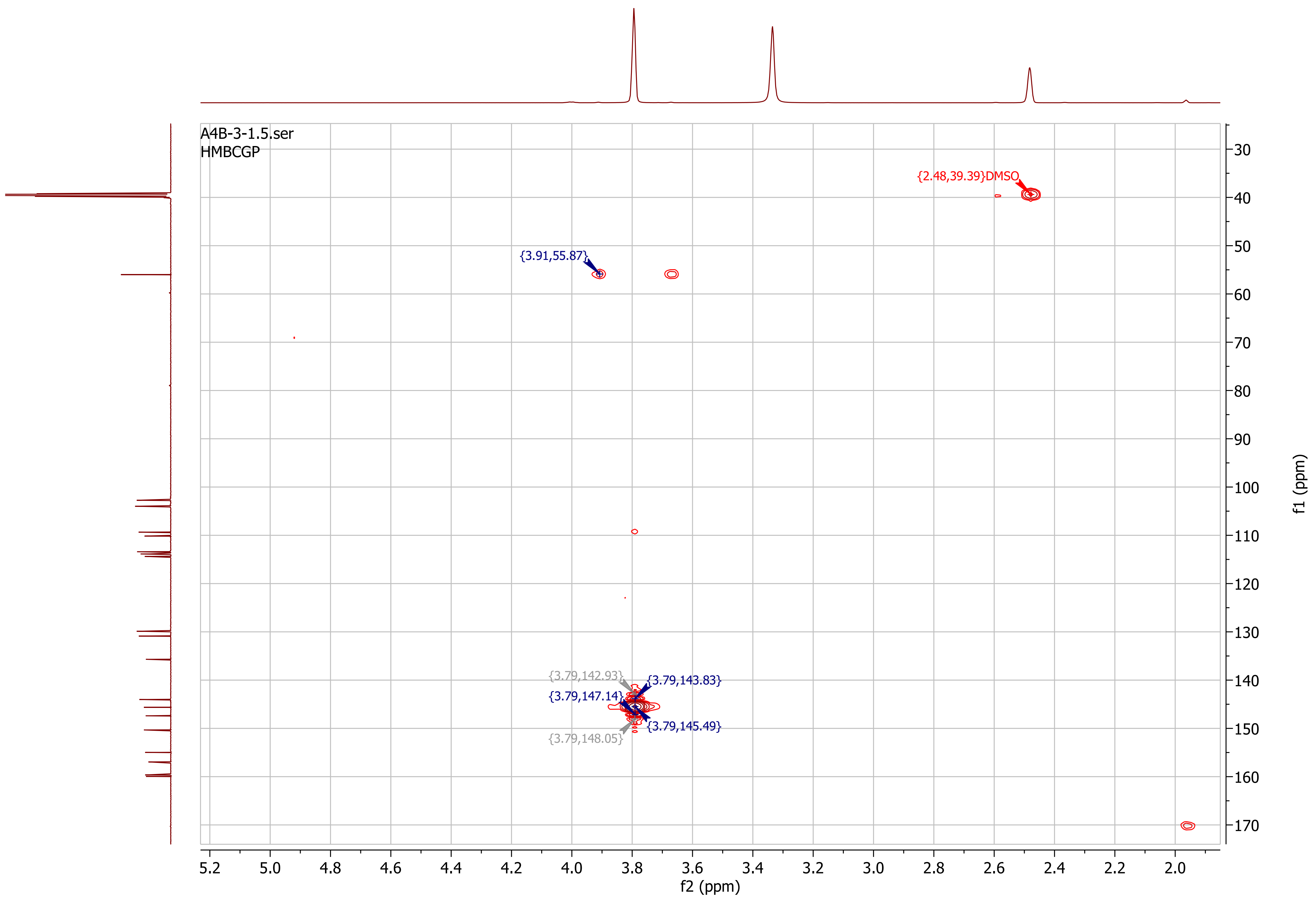

A4B-3-1.5.ser
HMBCGP
{2.48,39.39}DMSO
{3.91,55.87}
{3.79,142.93}
{3.79,143.83}
{3.79,147.14}
{3.79,145.49}
{3.79,148.05}
f2 (ppm)
f1 (ppm)

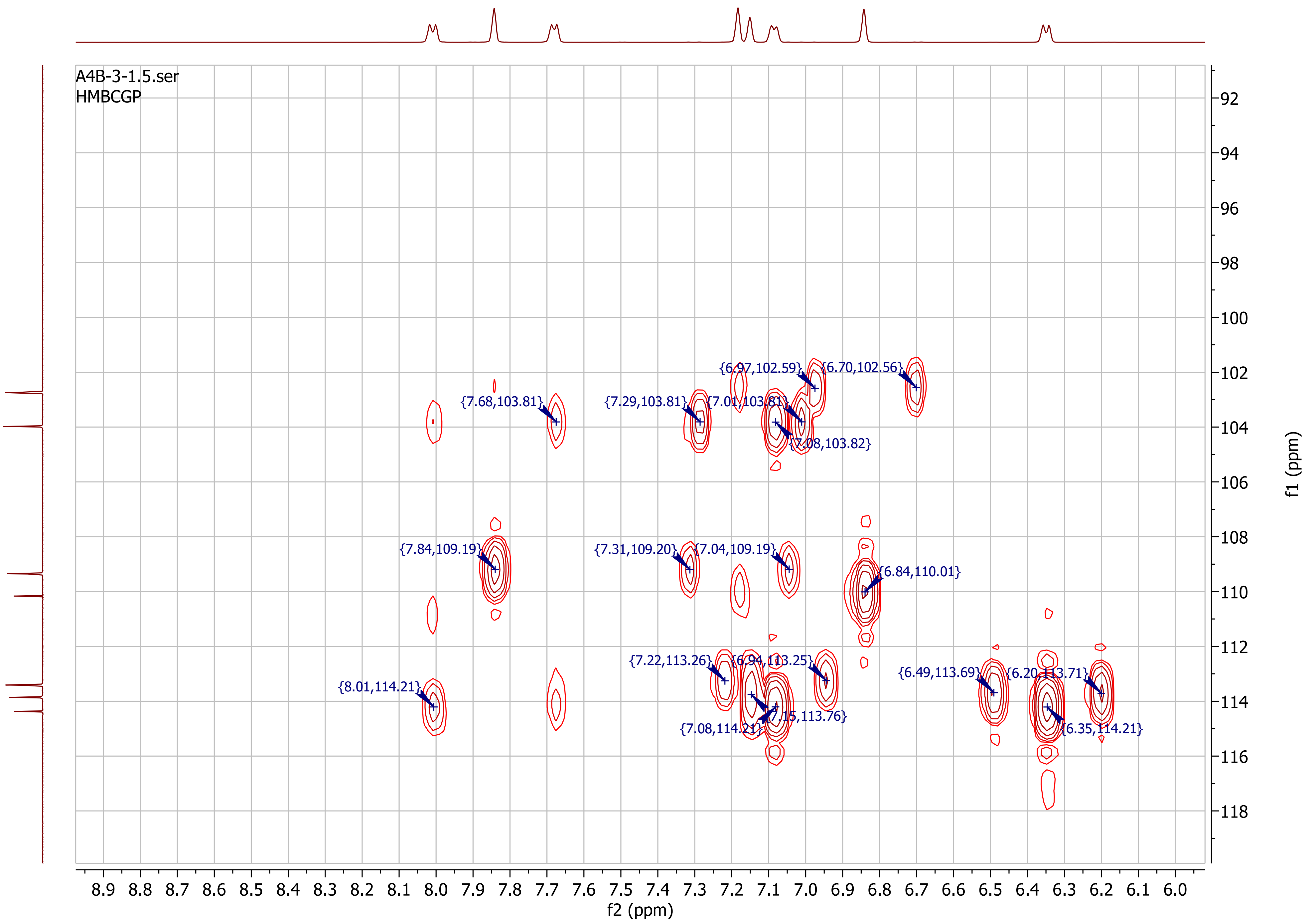
A4B-3-1.5.ser
HMBCGP
{6.97,102.59}
{6.70,102.56}
{7.68,103.81}
{7.29,103.81}
{7.01,103.81}
{7.08,103.82}
{7.84,109.19}
{7.31,109.20}
{7.04,109.19}
{6.84,110.01}
{7.22,113.26}
{6.94,113.25}
{8.01,114.21}
{6.49,113.69}
{6.20,113.71}
{7.15,113.76}
{7.08,114.21}
{6.35,114.21}
f2 (ppm)
f1 (ppm)

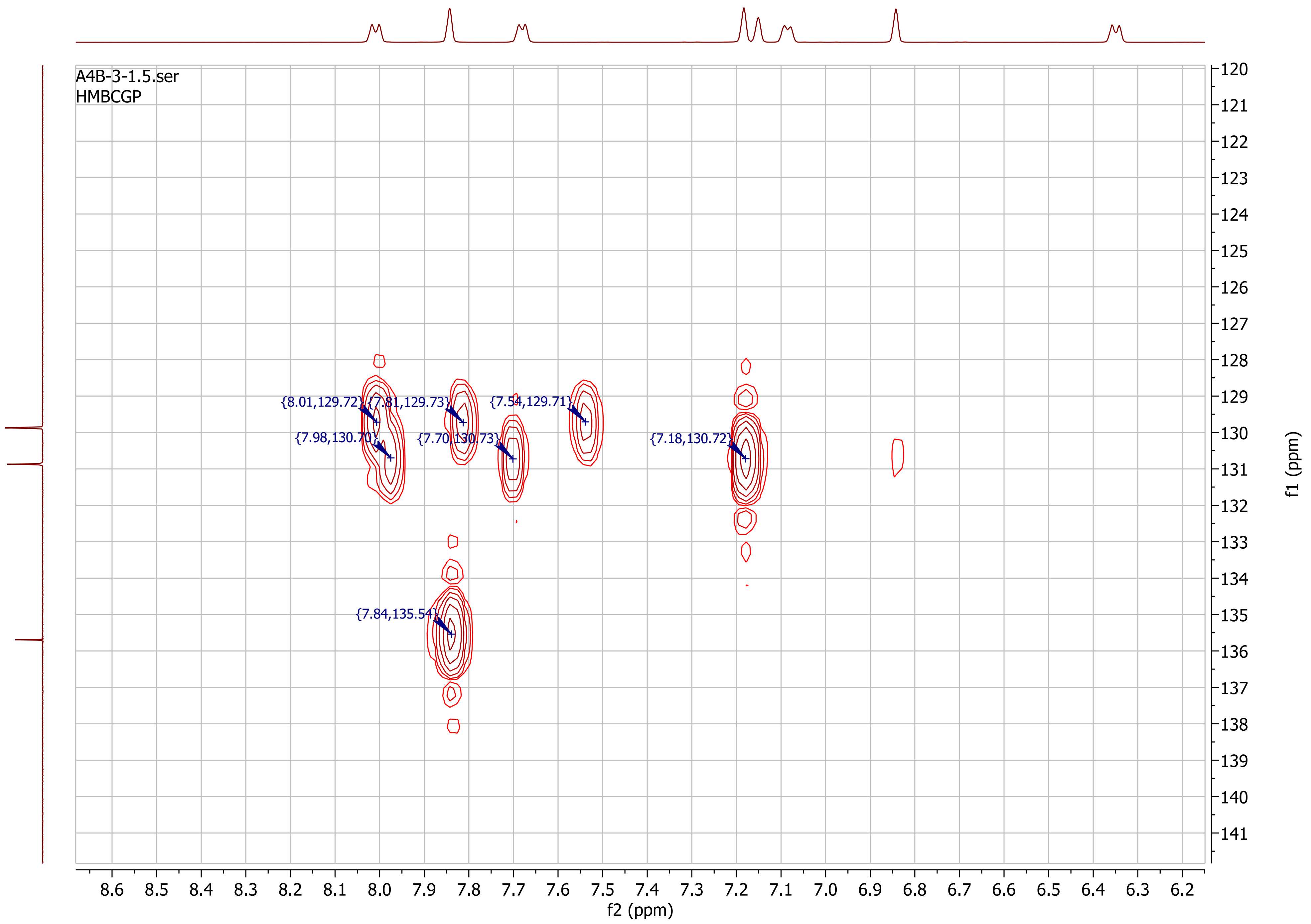

A4B-3-1.5.ser
HMBCGP
{8.01,129.72}
{7.81,129.73}
{7.54,129.71}
{7.98,130.70}
{7.70,130.73}
{7.18,130.72}
{7.84,135.54}
f2 (ppm)
f1 (ppm)

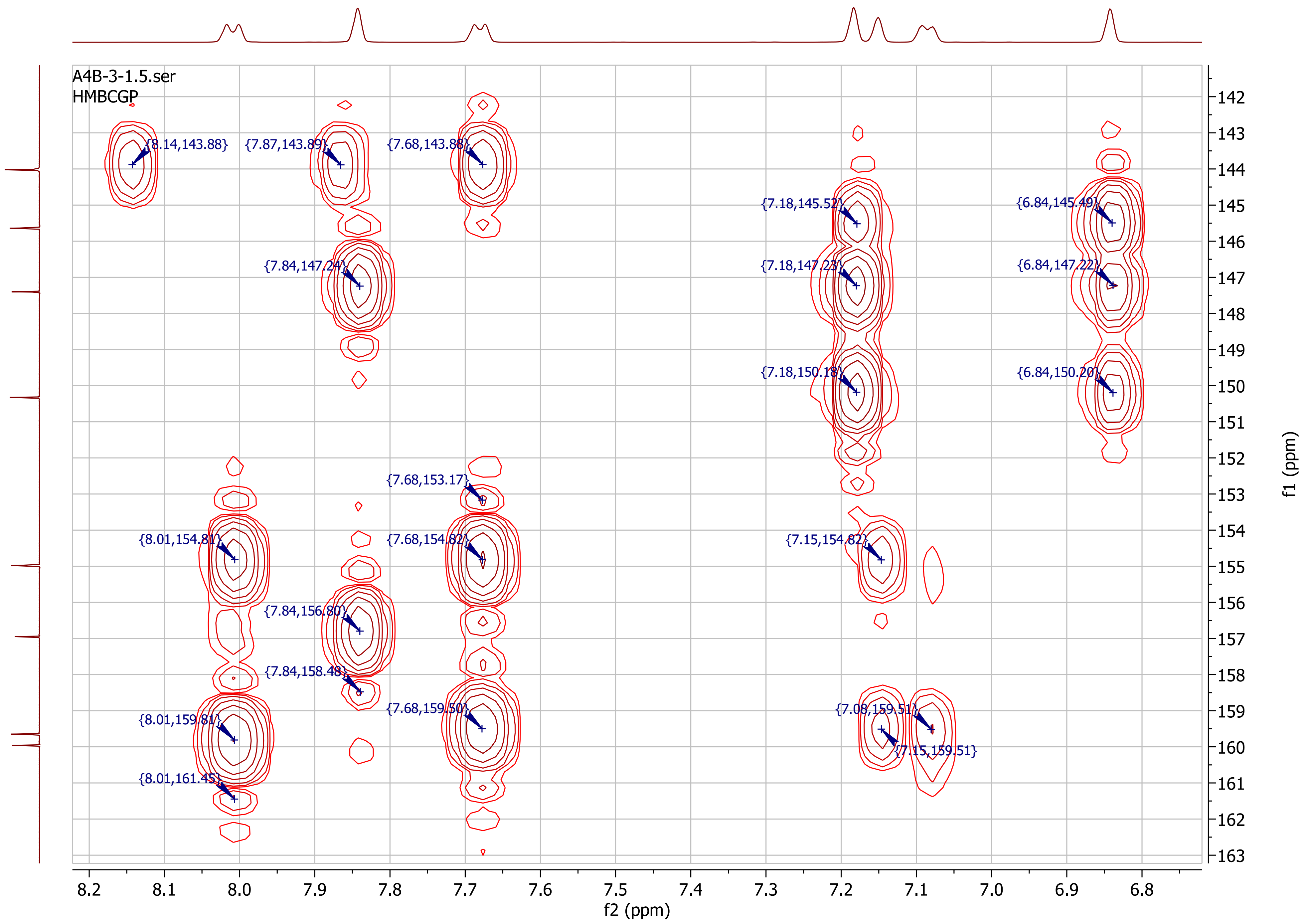

A4B-3-1.5.ser
HMBCGP
{8.14,143.88}
{7.87,143.89}
{7.68,143.88}
{7.18,145.52}
{6.84,145.49}
{7.84,147.24}
{7.18,147.23}
{6.84,147.22}
{7.18,150.18}
{6.84,150.20}
{7.68,153.17}
{8.01,154.81}
{7.68,154.82}
{7.15,154.82}
{7.84,156.80}
{7.84,158.48}
{8.01,159.81}
{7.68,159.50}
{7.08,159.51}
{7.15,159.51}
{8.01,161.45}
f2 (ppm)
f1 (ppm)

Toxicity Report

new dicoumarin.cdx

TOPKAT_Developmental_Toxicity_Potential

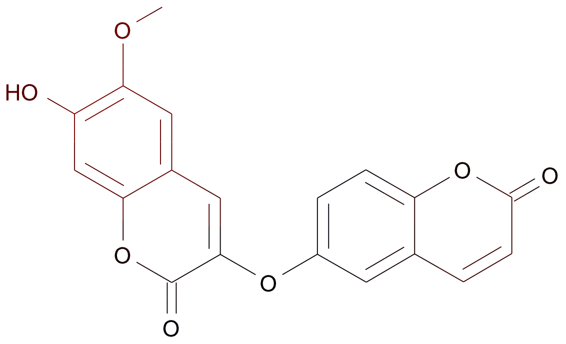

$C_{19}H_{12}O_7$

Molecular Weight: 352.29437

ALogP: 2.975

Rotatable Bonds: 3

Acceptors: 7

Donors: 1

Model Prediction

Prediction: Toxic

Probability: 0.718

Enrichment: 1.37

Bayesian Score: 4.4

Mahalanobis Distance: 11.2

Mahalanobis Distance p-value: 0.000474

Prediction: Positive if the Bayesian score is above the estimated best cutoff value from minimizing the false positive and false negative rate.
Probability: The esimated probability that the sample is in the positive category. This assumes that the Bayesian score follows a normal distribution and is different from the prediction using a cutoff.
Enrichment: An estimate of enrichment, that is, the increased likelihood (versus random) of this sample being in the category.
Bayesian Score: The standard Laplacian-modified Bayesian score.
Mahalanobis Distance: The Mahalanobis distance (MD) is the distance to the center of the training data. The larger the MD, the less trustworthy the prediction.
Mahalanobis Distance p-value: The p-value gives the fraction of training data with an MD greater than or equal to the one for the given sample, assuming normally distributed data. The smaller the p-value, the less trustworthy the prediciton. For highly non-normal X properties (e.g., fingerprints), the MD p-value is wildly inaccurate.

Structural Similar Compounds

Name	Prazosin .HCl (Free base form)	Bunazosin .HCl (Free base form)	D&C Yellow 8
Structure	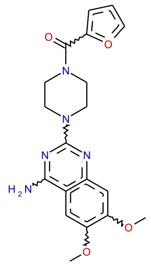	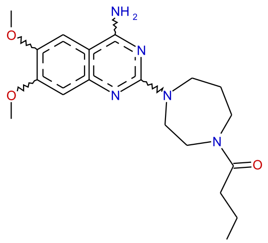	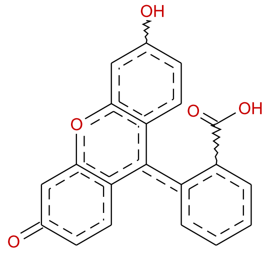
Actual Endpoint	Toxic	Toxic	Non-Toxic
Predicted Endpoint	Toxic	Toxic	Non-Toxic
Distance	0.554	0.603	0.610
Reference	Oyo Yakuri 17:57-62; 1979	Kiso to Rinsho 17:914-924; 1983	Food Chem Toxicol 24:819-823; 1986

Model Applicability

Unknown features are fingerprint features in the query molecule, but not found or appearing too infrequently in the training set.

1. All properties and OPS components are within expected ranges.

Feature Contribution

Top features for positive contribution				
Fingerprint	Bit/Smiles	Feature Structure	Score	Toxic in training set
SCFP_6	2116304939	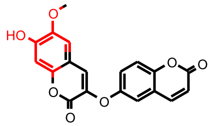 [*]O[c]1:[cH]:[*]:[c]([*]):[cH]:[c]:1O	0.504	5 out of 5

SCFP_6	-1977229858	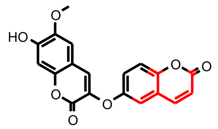<br>[*]=C1[*][c](:[*]):[c<br>](:[cH]:[*])C=C1	0.478	4 out of 4
SCFP_6	-1971137145	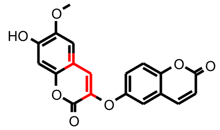<br>[*]C(=C[c](:[*]):[*])<br>[*]	0.431	7 out of 8

Top Features for negative contribution

Fingerprint	Bit/Smiles	Feature Structure	Score	Toxic in training set
SCFP_6	-609499983	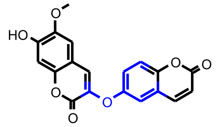<br>[*]C(=[*])O[c]1:[cH]:<br>[cH]:[*]:[c]([*]):[c<br>H]:1	-0.422	0 out of 1
SCFP_6	1132907712	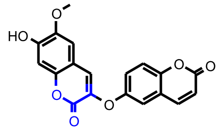<br>[*]OC(=O)C(=[*])[*]	-0.184	9 out of 21
SCFP_6	12	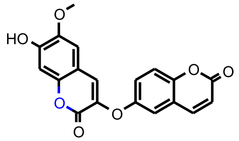<br>[*]O[*]	0	97 out of 178

remdesivir

TOPKAT_Developmental_Toxicity_Potential

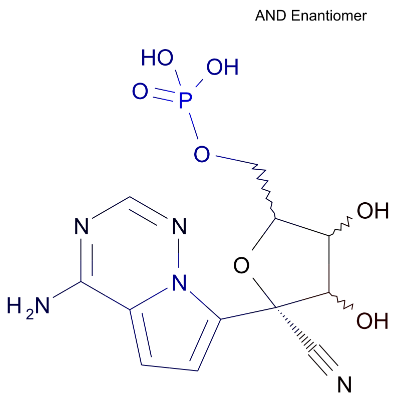

$C_{12}H_{14}N_5O_7P$
Molecular Weight: 371.24262
ALogP: -1.502
Rotatable Bonds: 4
Acceptors: 11
Donors: 5

Model Prediction

Prediction: Non-Toxic
Probability: 0.373
Enrichment: 0.709
Bayesian Score: -5.42
Mahalanobis Distance: 9.05
Mahalanobis Distance p-value: 0.163

Prediction: Positive if the Bayesian score is above the estimated best cutoff value from minimizing the false positive and false negative rate.
Probability: The esimated probability that the sample is in the positive category. This assumes that the Bayesian score follows a normal distribution and is different from the prediction using a cutoff.
Enrichment: An estimate of enrichment, that is, the increased likelihood (versus random) of this sample being in the category.
Bayesian Score: The standard Laplacian-modified Bayesian score.
Mahalanobis Distance: The Mahalanobis distance (MD) is the distance to the center of the training data. The larger the MD, the less trustworthy the prediction.
Mahalanobis Distance p-value: The p-value gives the fraction of training data with an MD greater than or equal to the one for the given sample, assuming normally distributed data. The smaller the p-value, the less trustworthy the prediciton. For highly non-normal X properties (e.g., fingerprints), the MD p-value is wildly inaccurate.

Structural Similar Compounds

Name	Sinigrin (Free Acid Form)	Azthreonam	Vidarabine
Structure	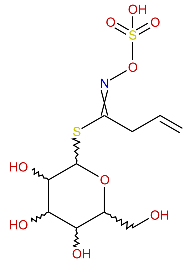	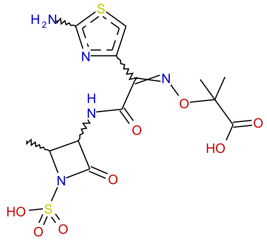	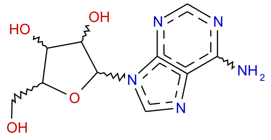
Actual Endpoint	Toxic	Non-Toxic	Non-Toxic
Predicted Endpoint	Toxic	Non-Toxic	Non-Toxic
Distance	0.632	0.707	0.714
Reference	Food Cosmet Toxicol 18(2):159-72; 1980	Chemotherapy 33:203-218; 1985	Teratology 15(3):231-41; 1977

Model Applicability

Unknown features are fingerprint features in the query molecule, but not found or appearing too infrequently in the training set.

1. All properties and OPS components are within expected ranges.

Feature Contribution

Top features for positive contribution				
**Fingerprint**	**Bit/Smiles**	**Feature Structure**	**Score**	**Toxic in training set**
SCFP_6	-1486266146	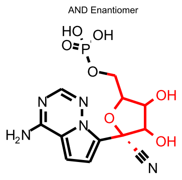 [*]CC1OC([*])([*])C(O)C1O	0.431	7 out of 8

SCFP_6	-1715619483	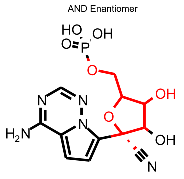 [*]OCC1OC([*])([*])C([*])C1O	0.298	6 out of 8
SCFP_6	-1181430618	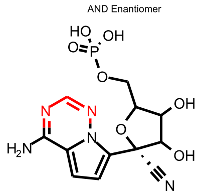 [*]:n:[cH]:n:[*]	0.298	6 out of 8
**Top Features for negative contribution**				
**Fingerprint**	**Bit/Smiles**	**Feature Structure**	**Score**	**Toxic in training set**
SCFP_6	2108966103	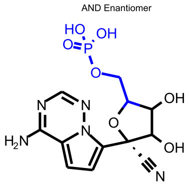 [*]C([*])COP(=O)(O)O	-0.945	0 out of 3
SCFP_6	-1375522316	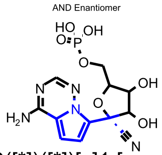 [*]C([*])([*])[c]1:[cH]:[cH]:[c](:[*]):n:1:[*]	-0.945	0 out of 3
SCFP_6	269938867	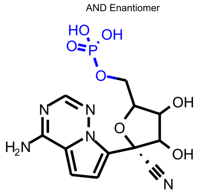 [*]OP(=O)(O)O	-0.729	1 out of 6

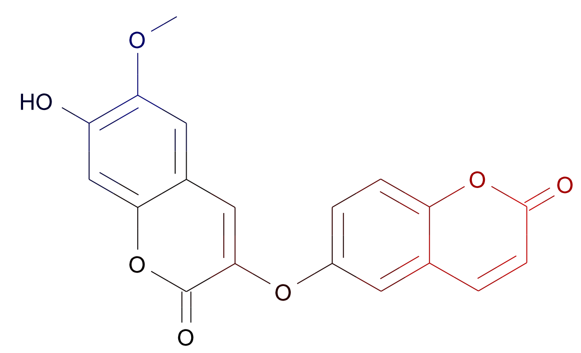

$C_{19}H_{12}O_7$

Molecular Weight: 352.29437

ALogP: 2.975

Rotatable Bonds: 3

Acceptors: 7

Donors: 1

Structural Similar Compounds

Name	Podofilox	Griseofulvin	Terazosin
Structure	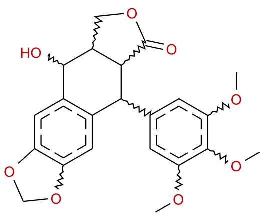	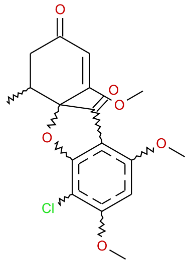	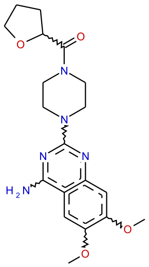
Actual Endpoint	Non-Carcinogen	Carcinogen	Non-Carcinogen
Predicted Endpoint	Non-Carcinogen	Carcinogen	Non-Carcinogen
Distance	0.621	0.640	0.650
Reference	US FDA (Centre for Drug Eval.& Res./Off. Testing & Res.) Sept. 1997	US FDA (Centre for Drug Eval.& Res./Off. Testing & Res.) Sept. 1997	US FDA (Centre for Drug Eval.& Res./Off. Testing & Res.) Sept. 1997

Model Prediction

Prediction: Carcinogen

Probability: 0.312

Enrichment: 0.974

Bayesian Score: 2

Mahalanobis Distance: 11.9

Mahalanobis Distance p-value: 0.0124

Prediction: Positive if the Bayesian score is above the estimated best cutoff value from minimizing the false positive and false negative rate.
Probability: The esimated probability that the sample is in the positive category. This assumes that the Bayesian score follows a normal distribution and is different from the prediction using a cutoff.
Enrichment: An estimate of enrichment, that is, the increased likelihood (versus random) of this sample being in the category.
Bayesian Score: The standard Laplacian-modified Bayesian score.
Mahalanobis Distance: The Mahalanobis distance (MD) is the distance to the center of the training data. The larger the MD, the less trustworthy the prediction.
Mahalanobis Distance p-value: The p-value gives the fraction of training data with an MD greater than or equal to the one for the given sample, assuming normally distributed data. The smaller the p-value, the less trustworthy the prediciton. For highly non-normal X properties (e.g., fingerprints), the MD p-value is wildly inaccurate.

Model Applicability

Unknown features are fingerprint features in the query molecule, but not found or appearing too infrequently in the training set.

1. All properties and OPS components are within expected ranges.
2. Unknown ECFP_2 feature: -741588105: [*]O\C(=C\[*])\C(=[*])[*]

Feature Contribution

Top features for positive contribution				
**Fingerprint**	**Bit/Smiles**	**Feature Structure**	**Score**	**Carcinogen in training set**
ECFP_6	1745066357	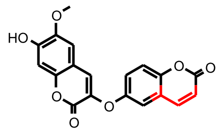 [*]\C=C/[c](:[*]):[*]	0.529	7 out of 12

ECFP_6	464808839	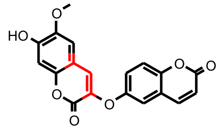 [*]C(=C[c](:[*]):[*]) [*]	0.524	8 out of 14
ECFP_6	1003935500	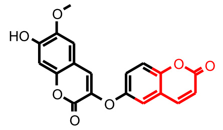 [*]:[cH]:[c]1C=CC(=O) O[c]:1:[*]	0.424	1 out of 1

Top Features for negative contribution

Fingerprint	Bit/Smiles	Feature Structure	Score	Carcinogen in training set
ECFP_6	1307307440	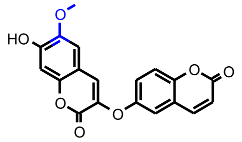 [*]:[c](:[*])OC	-0.558	4 out of 25
ECFP_6	1334400011	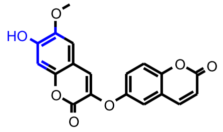 [*][c](:[*]):[c](O):[cH]:[*]	-0.496	3 out of 18
ECFP_6	864909220	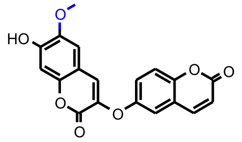 [*]OC	-0.466	7 out of 38

remdesivir

TOPKAT_Mouse_Female_FDA_None_vs_Carcinogen

AND Enantiomer

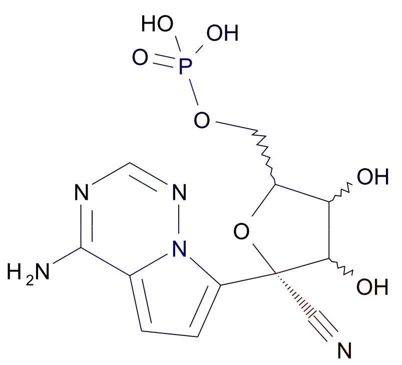

$C_{12}H_{14}N_5O_7P$
Molecular Weight: 371.24262
ALogP: -1.502
Rotatable Bonds: 4
Acceptors: 11
Donors: 5

Structural Similar Compounds

Name	Famotidine	Tetracycline	Oxytetracycline
Structure	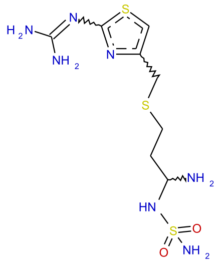	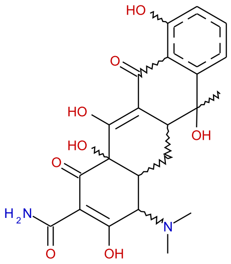	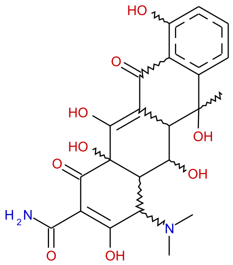
Actual Endpoint	Non-Carcinogen	Non-Carcinogen	Non-Carcinogen
Predicted Endpoint	Non-Carcinogen	Non-Carcinogen	Non-Carcinogen
Distance	0.846	0.848	0.870
Reference	US FDA (Centre for Drug Eval.& Res./Off. Testing & Res.) Sept. 1997	US FDA (Centre for Drug Eval.& Res./Off. Testing & Res.) Sept. 1997	US FDA (Centre for Drug Eval.& Res./Off. Testing & Res.) Sept. 1997

Model Prediction

Prediction: Non-Carcinogen
Probability: 0.206
Enrichment: 0.642
Bayesian Score: -7.17
Mahalanobis Distance: 12.9
Mahalanobis Distance p-value: 0.00074

Prediction: Positive if the Bayesian score is above the estimated best cutoff value from minimizing the false positive and false negative rate.
Probability: The esimated probability that the sample is in the positive category. This assumes that the Bayesian score follows a normal distribution and is different from the prediction using a cutoff.
Enrichment: An estimate of enrichment, that is, the increased likelihood (versus random) of this sample being in the category.
Bayesian Score: The standard Laplacian-modified Bayesian score.
Mahalanobis Distance: The Mahalanobis distance (MD) is the distance to the center of the training data. The larger the MD, the less trustworthy the prediction.
Mahalanobis Distance p-value: The p-value gives the fraction of training data with an MD greater than or equal to the one for the given sample, assuming normally distributed data. The smaller the p-value, the less trustworthy the prediciton. For highly non-normal X properties (e.g., fingerprints), the MD p-value is wildly inaccurate.

Model Applicability

Unknown features are fingerprint features in the query molecule, but not found or appearing too infrequently in the training set.

1. All properties and OPS components are within expected ranges.
2. Unknown ECFP_2 feature: 1126642748: [*]OP(=O)(O)O
3. Unknown ECFP_2 feature: -1250439909: [*]COP(=[*])([*])[*]
4. Unknown ECFP_2 feature: 1258791451: [*]C1[*][*]O[C@]1(C#[*])[c](:[*]):[*]
5. Unknown ECFP_2 feature: -1507082173: [*][c]1:[*]:[*]:[c](:[*]):n:1:n:[*]
6. Unknown ECFP_2 feature: -66263742: [*]C([*])([*])[c]1:[cH]:[*]:[*]:n:1:[*]

Feature Contribution

Top features for positive contribution

Fingerprint	Bit/Smiles	Feature Structure	Score	Carcinogen in training set

ECFP_6	-1114776580	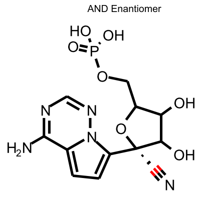 [*]C#[*]	0.755	11 out of 15
ECFP_6	-521596699	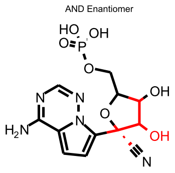 [*]C1[*][*]C([*])([*])C1O	0.451	3 out of 5
ECFP_6	-264833661	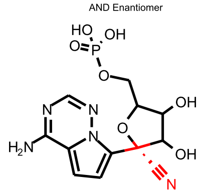 [*]C([*])([*])C#N	0.424	1 out of 1
**Top Features for negative contribution**				
**Fingerprint**	**Bit/Smiles**	**Feature Structure**	**Score**	**Carcinogen in training set**
ECFP_6	1334415134	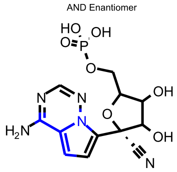 [*][c](:[*]):[c]1:[cH]:[*]:[*]:n:1:[*]	-0.935	0 out of 5
ECFP_6	2100964382	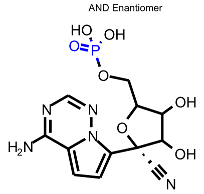 [*]P(=O)([*])[*]	-0.935	0 out of 5

ECFP_6	-826638028	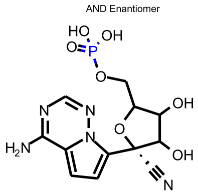 [*]P(=[*])([*])[*]	-0.935	0 out of 5

new dicoumarin.cdx

TOPKAT_Mouse_Female_FDA_Single_vs_Multiple

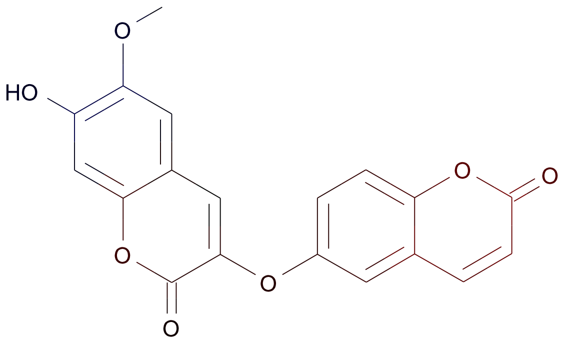

$C_{19}H_{12}O_7$
Molecular Weight: 352.29437
ALogP: 2.975
Rotatable Bonds: 3
Acceptors: 7
Donors: 1

Model Prediction

Prediction: Multiple-Carcinogen
Probability: 0.486
Enrichment: 1.19
Bayesian Score: 1.9
Mahalanobis Distance: 10
Mahalanobis Distance p-value: 0.0151

Prediction: Positive if the Bayesian score is above the estimated best cutoff value from minimizing the false positive and false negative rate.
Probability: The esimated probability that the sample is in the positive category. This assumes that the Bayesian score follows a normal distribution and is different from the prediction using a cutoff.
Enrichment: An estimate of enrichment, that is, the increased likelihood (versus random) of this sample being in the category.
Bayesian Score: The standard Laplacian-modified Bayesian score.
Mahalanobis Distance: The Mahalanobis distance (MD) is the distance to the center of the training data. The larger the MD, the less trustworthy the prediction.
Mahalanobis Distance p-value: The p-value gives the fraction of training data with an MD greater than or equal to the one for the given sample, assuming normally distributed data. The smaller the p-value, the less trustworthy the prediciton. For highly non-normal X properties (e.g., fingerprints), the MD p-value is wildly inaccurate.

Structural Similar Compounds

Name	Griseofulvin	Moricizine	Flunisolide
Structure	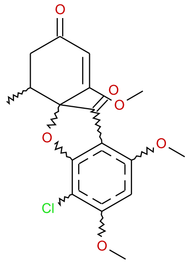	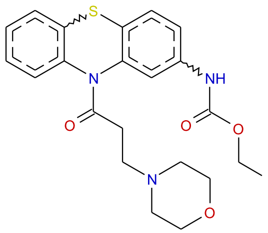	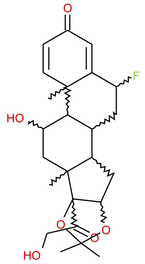
Actual Endpoint	Single-Carcinogen	Single-Carcinogen	Multiple-Carcinogen
Predicted Endpoint	Single-Carcinogen	Single-Carcinogen	Multiple-Carcinogen
Distance	0.647	0.708	0.780
Reference	US FDA (Centre for Drug Eval.& Res./Off. Testing & Res.) Sept. 1997	US FDA (Centre for Drug Eval.& Res./Off. Testing & Res.) Sept. 1997	US FDA (Centre for Drug Eval.& Res./Off. Testing & Res.) Sept. 1997

Model Applicability

Unknown features are fingerprint features in the query molecule, but not found or appearing too infrequently in the training set.

1. OPS PC16 out of range. Value: 2.7241. Training min, max, SD, explained variance: -2.4118, 2.6996, 0.9803, 0.0176.
2. Unknown ECFP_2 feature: -741588105: [*]O\C(=C\[*])\C(=[*])[*]

Feature Contribution

Top features for positive contribution

Fingerprint	Bit/Smiles	Feature Structure	Score	Multiple-Carcinogen in training set
ECFP_4	1573945311	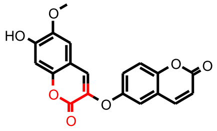 [*]OC(=O)C(=[*])[*]	0.351	1 out of 1

ECFP_4	1305253718	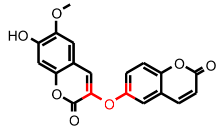 [*]C(=[*])O[c](:[*]):[*]	0.351	1 out of 1
ECFP_4	-1885846789	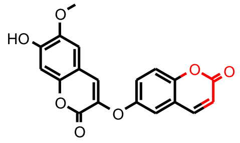 [*]OC(=O)C=[*]	0.351	1 out of 1

Top Features for negative contribution

Fingerprint	Bit/Smiles	Feature Structure	Score	Multiple-Carcinogen in training set
ECFP_4	1334400011	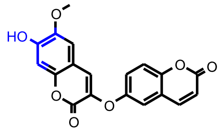 [*][c](:[*]):[c](O):[cH]:[*]	-0.8	0 out of 3
ECFP_4	1408898974	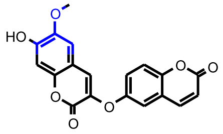 [*]O[c](:[cH]:[*]):[c]([*]):[*]	-0.545	1 out of 6
ECFP_4	464808839	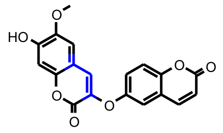 [*]C(=C[c](:[*]):[*])[*]	-0.352	2 out of 8

new dicoumarin.cdx

TOPKAT_Mouse_Male_FDA_None_vs_Carcinogen

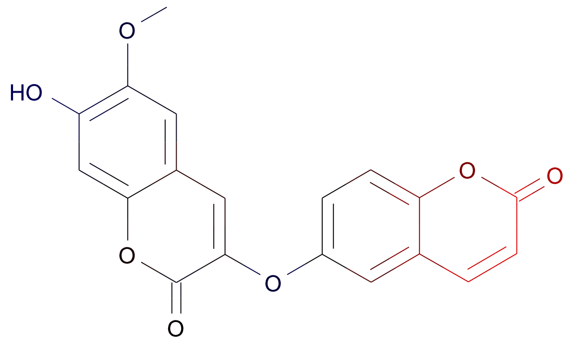

$C_{19}H_{12}O_7$
Molecular Weight: 352.29437
ALogP: 2.975
Rotatable Bonds: 3
Acceptors: 7
Donors: 1

Model Prediction

Prediction: Carcinogen
Probability: 0.346
Enrichment: 1.17
Bayesian Score: 1.37
Mahalanobis Distance: 14.6
Mahalanobis Distance p-value: 4.87e-007

Prediction: Positive if the Bayesian score is above the estimated best cutoff value from minimizing the false positive and false negative rate.
Probability: The esimated probability that the sample is in the positive category. This assumes that the Bayesian score follows a normal distribution and is different from the prediction using a cutoff.
Enrichment: An estimate of enrichment, that is, the increased likelihood (versus random) of this sample being in the category.
Bayesian Score: The standard Laplacian-modified Bayesian score.
Mahalanobis Distance: The Mahalanobis distance (MD) is the distance to the center of the training data. The larger the MD, the less trustworthy the prediction.
Mahalanobis Distance p-value: The p-value gives the fraction of training data with an MD greater than or equal to the one for the given sample, assuming normally distributed data. The smaller the p-value, the less trustworthy the prediciton. For highly non-normal X properties (e.g., fingerprints), the MD p-value is wildly inaccurate.

Structural Similar Compounds

Name	Podofilox	Griseofulvin	Terazosin
Structure	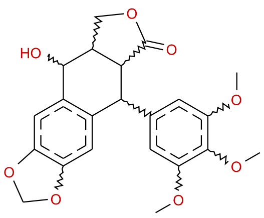	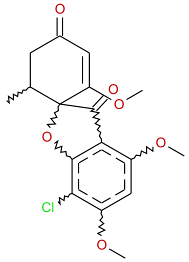	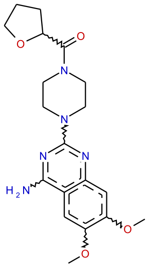
Actual Endpoint	Non-Carcinogen	Carcinogen	Non-Carcinogen
Predicted Endpoint	Non-Carcinogen	Carcinogen	Non-Carcinogen
Distance	0.617	0.628	0.647
Reference	US FDA (Centre for Drug Eval.& Res./Off. Testing & Res.) Sept. 1997	US FDA (Centre for Drug Eval.& Res./Off. Testing & Res.) Sept. 1997	US FDA (Centre for Drug Eval.& Res./Off. Testing & Res.) Sept. 1997

Model Applicability

Unknown features are fingerprint features in the query molecule, but not found or appearing too infrequently in the training set.

1. All properties and OPS components are within expected ranges.

Feature Contribution

Top features for positive contribution

Fingerprint	Bit/Smiles	Feature Structure	Score	Carcinogen in training set
FCFP_6	-146015125	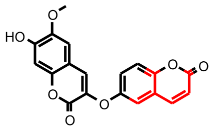 [*]=C1[*][c](:[*]):[c](:[cH]:[*])C=C1	0.676	2 out of 2

FCFP_6	451847724	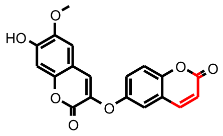 [*]\C=C/C(=[*])[*]	0.479	21 out of 48
FCFP_6	-948664610	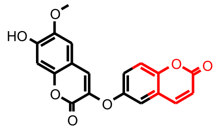 [*]:[cH]:[c]1OC(=O)C= C[c]:1:[*]	0.46	1 out of 1

Top Features for negative contribution

Fingerprint	Bit/Smiles	Feature Structure	Score	Carcinogen in training set
FCFP_6	356782498	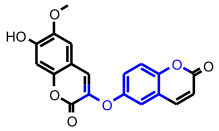 [*]O[c]1:[cH]:[cH]:[c](OC(=[*])[*]):[cH]: [c]:1[*]	-0.582	0 out of 3
FCFP_6	523826990	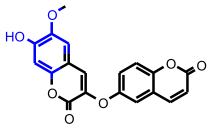 [*]O[c]1:[cH]:[*]:[c] ([*]):[cH]:[c]:1O	-0.423	0 out of 2
FCFP_6	7	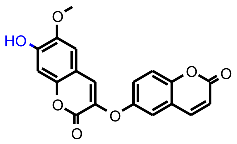 [*]O	-0.308	15 out of 79

remdesivir

TOPKAT_Mouse_Male_FDA_None_vs_Carcinogen

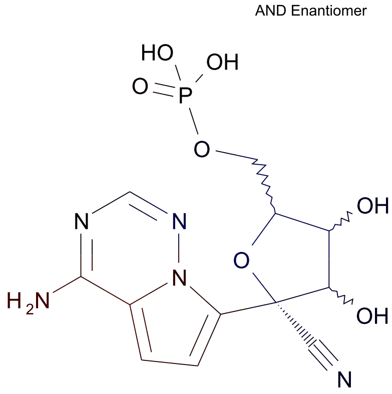

$C_{12}H_{14}N_5O_7P$
Molecular Weight: 371.24262
ALogP: -1.502
Rotatable Bonds: 4
Acceptors: 11
Donors: 5

Model Prediction

Prediction: Non-Carcinogen
Probability: 0.239
Enrichment: 0.812
Bayesian Score: -2.82
Mahalanobis Distance: 19.2
Mahalanobis Distance p-value: 7.81e-017

Prediction: Positive if the Bayesian score is above the estimated best cutoff value from minimizing the false positive and false negative rate.
Probability: The esimated probability that the sample is in the positive category. This assumes that the Bayesian score follows a normal distribution and is different from the prediction using a cutoff.
Enrichment: An estimate of enrichment, that is, the increased likelihood (versus random) of this sample being in the category.
Bayesian Score: The standard Laplacian-modified Bayesian score.
Mahalanobis Distance: The Mahalanobis distance (MD) is the distance to the center of the training data. The larger the MD, the less trustworthy the prediction.
Mahalanobis Distance p-value: The p-value gives the fraction of training data with an MD greater than or equal to the one for the given sample, assuming normally distributed data. The smaller the p-value, the less trustworthy the prediciton. For highly non-normal X properties (e.g., fingerprints), the MD p-value is wildly inaccurate.

Structural Similar Compounds

Name	Famotidine	Tetracycline	Ribavirin
Structure	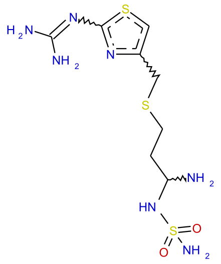	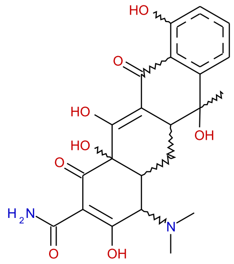	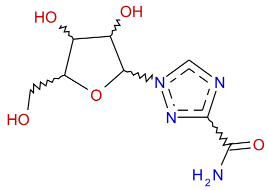
Actual Endpoint	Non-Carcinogen	Non-Carcinogen	Non-Carcinogen
Predicted Endpoint	Non-Carcinogen	Non-Carcinogen	Non-Carcinogen
Distance	0.813	0.843	0.860
Reference	US FDA (Centre for Drug Eval.& Res./Off. Testing & Res.) Sept. 1997	US FDA (Centre for Drug Eval.& Res./Off. Testing & Res.) Sept. 1997	US FDA (Centre for Drug Eval.& Res./Off. Testing & Res.) Sept. 1997

Model Applicability

Unknown features are fingerprint features in the query molecule, but not found or appearing too infrequently in the training set.

1. All properties and OPS components are within expected ranges.
2. Unknown FCFP_2 feature: 472180098: [*]OP(=O)(O)O
3. Unknown FCFP_2 feature: -332197802: [*][c]1:[*]:[*]:[c](:[*]):n:1:n:[*]

Feature Contribution

Top features for positive contribution

Fingerprint	Bit/Smiles	Feature Structure	Score	Carcinogen in training set
FCFP_6	-450797925	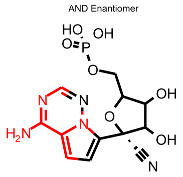 N[c]1:n:[cH]:[*]:n2:[*]:[*]:[cH]:[c]:1:2	0.676	2 out of 2

FCFP_6	-1151884458	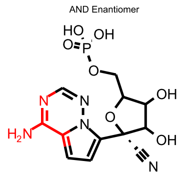[*]:n:[c](N):[c](:[*]<br>):[*]	0.348	6 out of 15
FCFP_6	-1280036918	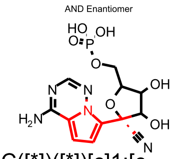[*]C([*])([*])[c]1:[c<br>H]:[cH]:[c](:[*]):n:<br>1:[*]	0.333	7 out of 18

Top Features for negative contribution

Fingerprint	Bit/Smiles	Feature Structure	Score	Carcinogen in training set
FCFP_6	-124685461	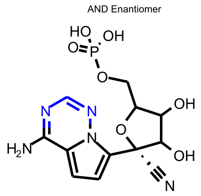[*]:n:[cH]:n:[*]	-0.731	1 out of 12
FCFP_6	-1277879912	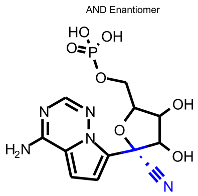[*]C([*])([*])C#N	-0.582	0 out of 3
FCFP_6	422052003	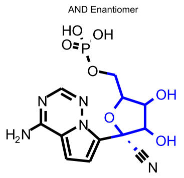[*]CC1OC([*])([*])C(O<br>)C1O	-0.582	0 out of 3

new dicoumarin.cdx

TOPKAT_Mouse_Male_FDA_Single_vs_Multiple

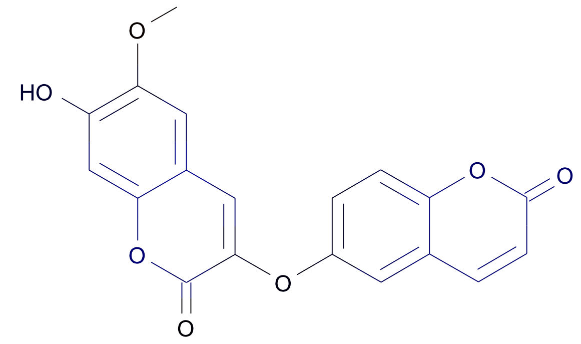

$C_{19}H_{12}O_7$
Molecular Weight: 352.29437
ALogP: 2.975
Rotatable Bonds: 3
Acceptors: 7
Donors: 1

Model Prediction

Prediction: Single-Carcinogen
Probability: 0.146
Enrichment: 0.485
Bayesian Score: -8.75
Mahalanobis Distance: 12
Mahalanobis Distance p-value: 0.000614

Prediction: Positive if the Bayesian score is above the estimated best cutoff value from minimizing the false positive and false negative rate.
Probability: The esimated probability that the sample is in the positive category. This assumes that the Bayesian score follows a normal distribution and is different from the prediction using a cutoff.
Enrichment: An estimate of enrichment, that is, the increased likelihood (versus random) of this sample being in the category.
Bayesian Score: The standard Laplacian-modified Bayesian score.
Mahalanobis Distance: The Mahalanobis distance (MD) is the distance to the center of the training data. The larger the MD, the less trustworthy the prediction.
Mahalanobis Distance p-value: The p-value gives the fraction of training data with an MD greater than or equal to the one for the given sample, assuming normally distributed data. The smaller the p-value, the less trustworthy the prediciton. For highly non-normal X properties (e.g., fingerprints), the MD p-value is wildly inaccurate.

Structural Similar Compounds

Name	Griseofulvin	Flunisolide	Lansoprazole
Structure	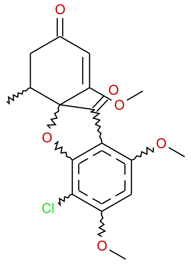	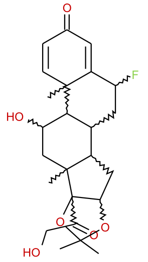	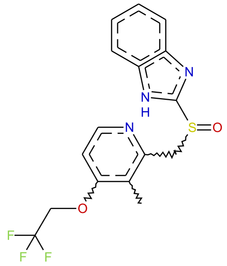
Actual Endpoint	Single-Carcinogen	Single-Carcinogen	Single-Carcinogen
Predicted Endpoint	Single-Carcinogen	Single-Carcinogen	Single-Carcinogen
Distance	0.690	0.820	0.842
Reference	US FDA (Centre for Drug Eval.& Res./Off. Testing & Res.) Sept. 1997	US FDA (Centre for Drug Eval.& Res./Off. Testing & Res.) Sept. 1997	US FDA (Centre for Drug Eval.& Res./Off. Testing & Res.) Sept. 1997

Model Applicability

Unknown features are fingerprint features in the query molecule, but not found or appearing too infrequently in the training set.

1. All properties and OPS components are within expected ranges.

Feature Contribution

Top features for positive contribution

Fingerprint	Bit/Smiles	Feature Structure	Score	Multiple-Carcinogen in training set
FCFP_12	451847724	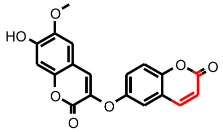 [*]\C=C/C(=[*])[*]	0.3	10 out of 21

FCFP_12	565998553	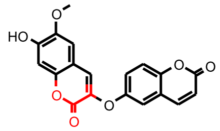 [*]OC(=O)C(=[*])[*]	0.194	6 out of 14
FCFP_12	1676877079	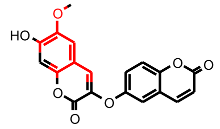 [*]O[c]1:[cH]:[c](C=[*]):[c]([*]):[*]:[c]:1[*]	0.168	3 out of 7
**Top Features for negative contribution**				
**Fingerprint**	**Bit/Smiles**	**Feature Structure**	**Score**	**Multiple-Carcinogen in training set**
FCFP_12	-1099193755	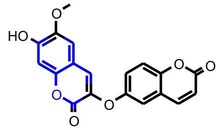 [*][c]1:[*]:[cH]:[c]2C=[*]C(=[*])O[c]:2:[cH]:1	-1.11	0 out of 6
FCFP_12	7	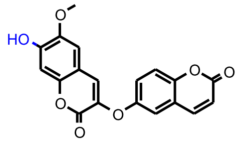 [*]O	-0.71	2 out of 15
FCFP_12	-146015125	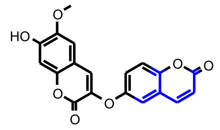 [*]=C1[*][c](:[*]):[c](:[cH]:[*])C=C1	-0.519	0 out of 2

new dicoumarin.cdx **TOPKAT_Ocular_Irritancy_Mild_vs_Moderate_Severe**

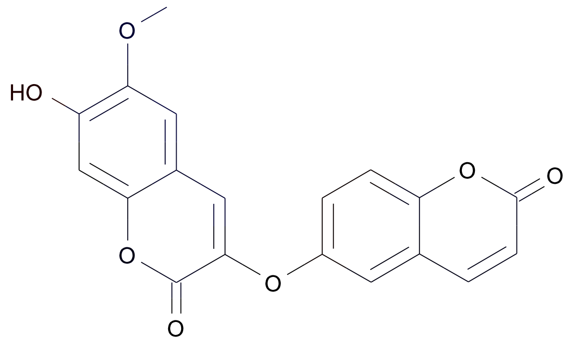

$C_{19}H_{12}O_7$
Molecular Weight: 352.29437
ALogP: 2.975
Rotatable Bonds: 3
Acceptors: 7
Donors: 1

Structural Similar Compounds

Name	COLCHICINE	2;5-DICHLORO-4(3'-METHYL-5' PYRAZOLON-1'-YL)BENZENE SULFONIC ACID	1;8;9-ANTHRACENETRIOL; TRIACETATE
Structure	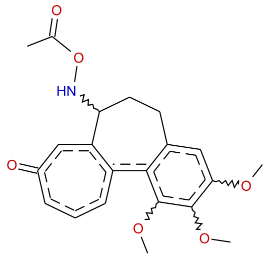	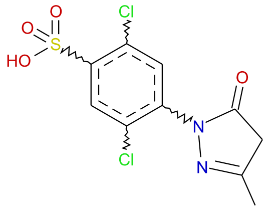	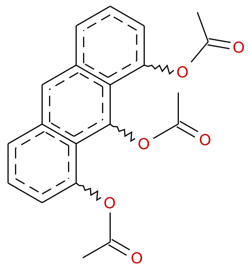
Actual Endpoint	Moderate_Severe	Mild	Moderate_Severe
Predicted Endpoint	Moderate_Severe	Mild	Moderate_Severe
Distance	0.621	0.709	0.729
Reference	AJOPAA 31;837;48	28ZPAK-;186;72	BJOPAL 53;819;69

Model Prediction

Prediction: Mild
Probability: 0.778
Enrichment: 1.13
Bayesian Score: -1.75
Mahalanobis Distance: 9.34
Mahalanobis Distance p-value: 0.332

Prediction: Positive if the Bayesian score is above the estimated best cutoff value from minimizing the false positive and false negative rate.
Probability: The esimated probability that the sample is in the positive category. This assumes that the Bayesian score follows a normal distribution and is different from the prediction using a cutoff.
Enrichment: An estimate of enrichment, that is, the increased likelihood (versus random) of this sample being in the category.
Bayesian Score: The standard Laplacian-modified Bayesian score.
Mahalanobis Distance: The Mahalanobis distance (MD) is the distance to the center of the training data. The larger the MD, the less trustworthy the prediction.
Mahalanobis Distance p-value: The p-value gives the fraction of training data with an MD greater than or equal to the one for the given sample, assuming normally distributed data. The smaller the p-value, the less trustworthy the prediciton. For highly non-normal X properties (e.g., fingerprints), the MD p-value is wildly inaccurate.

Model Applicability

Unknown features are fingerprint features in the query molecule, but not found or appearing too infrequently in the training set.

1. All properties and OPS components are within expected ranges.

Feature Contribution

Top features for positive contribution

Fingerprint	Bit/Smiles	Feature Structure	Score	Moderate_Severe in training set
FCFP_10	7	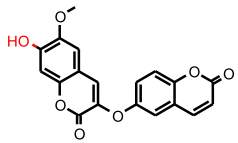 [*]O	0.219	117 out of 142

FCFP_10	346218766	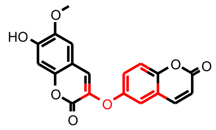<br>[*]C(=[*])O[c]1:[cH]:<br>[cH]:[*]:[c]([*]):[c<br>H]:1	0.197	30 out of 37
FCFP_10	523826990	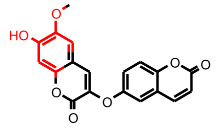<br>[*]O[c]1:[cH]:[*]:[c]<br>([*]):[cH]:[c]:1O	0.186	1 out of 1

Top Features for negative contribution

**Fingerprint**	**Bit/Smiles**	**Feature Structure**	**Score**	**Moderate_Severe in training set**
FCFP_10	-1977641857	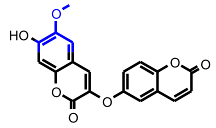<br>[*][c](:[*]):[c](OC):<br>[cH]:[*]	-0.78	4 out of 15
FCFP_10	-146015125	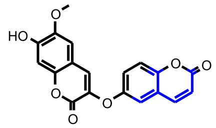<br>[*]=C1[*][c](:[*]):[c<br>](:[cH]:[*])C=C1	-0.507	0 out of 1
FCFP_10	-1099193755	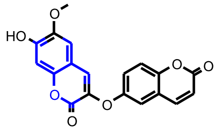<br>[*][c]1:[*]:[cH]:[c]2<br>C=[*]C(=[*])O[c]:2:[<br>cH]:1	-0.361	2 out of 5

remdesivir

TOPKAT_Ocular_Irritancy_Mild_vs_Moderate_Severe

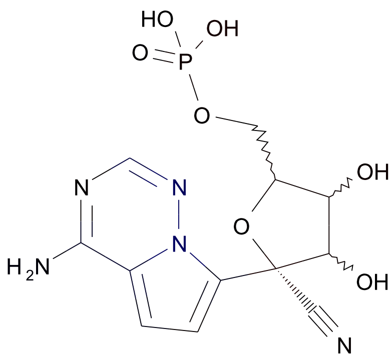

$C_{12}H_{14}N_5O_7P$

Molecular Weight: 371.24262

ALogP: -1.502

Rotatable Bonds: 4

Acceptors: 11

Donors: 5

Structural Similar Compounds

Name	1;3;6-NAPHTHALENE TRISULFONIC ACID;7-AMINO-	Methanol; (s-triazine-2;4;6-triyltrinitrilo)hexa-	2;2'-Biphenyldisulfonic acid; 4;4'-diamino-
Structure	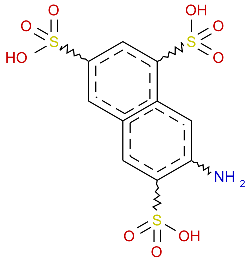	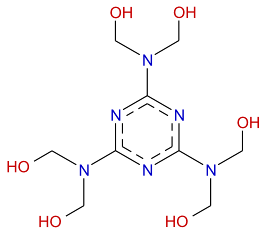	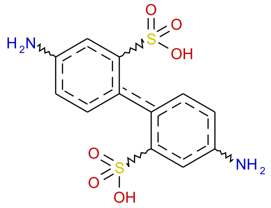
Actual Endpoint	Mild	Moderate_Severe	Mild
Predicted Endpoint	Mild	Moderate_Severe	Mild
Distance	0.776	0.802	0.878
Reference	28ZPAK-;190;72	Prehled Prumyslove Toxikologie; Organicke Latky; Marhold; J. -;876;86	Prehled Prumyslove Toxikologie; Organicke Latky; Marhold; J. pp 1061;86

Model Prediction

Prediction: Mild

Probability: 0.789

Enrichment: 1.15

Bayesian Score: -1.39

Mahalanobis Distance: 13.8

Mahalanobis Distance p-value: 1.42e-009

Prediction: Positive if the Bayesian score is above the estimated best cutoff value from minimizing the false positive and false negative rate.
Probability: The esimated probability that the sample is in the positive category. This assumes that the Bayesian score follows a normal distribution and is different from the prediction using a cutoff.
Enrichment: An estimate of enrichment, that is, the increased likelihood (versus random) of this sample being in the category.
Bayesian Score: The standard Laplacian-modified Bayesian score.
Mahalanobis Distance: The Mahalanobis distance (MD) is the distance to the center of the training data. The larger the MD, the less trustworthy the prediction.
Mahalanobis Distance p-value: The p-value gives the fraction of training data with an MD greater than or equal to the one for the given sample, assuming normally distributed data. The smaller the p-value, the less trustworthy the prediciton. For highly non-normal X properties (e.g., fingerprints), the MD p-value is wildly inaccurate.

Model Applicability

Unknown features are fingerprint features in the query molecule, but not found or appearing too infrequently in the training set.

1. OPS PC17 out of range. Value: 4.6782. Training min, max, SD, explained variance: -4.348, 3.9505, 1.094, 0.0146.
2. Unknown FCFP_2 feature: 472180098: [*]OP(=O)(O)O
3. Unknown FCFP_2 feature: -836603894: [*]C1[*][*]O[C@]1(C#[*])[c](:[*]):[*]
4. Unknown FCFP_2 feature: -124685461: [*]:n:[cH]:n:[*]
5. Unknown FCFP_2 feature: -1151884458: [*]:n:[c](N):[c](:[*]):[*]

Feature Contribution

Top features for positive contribution

Fingerprint	Bit/Smiles	Feature Structure	Score	Moderate_Severe in training set

FCFP_10	1070061035	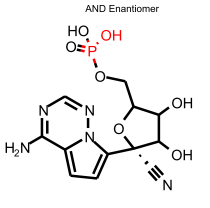 [*]P(=[*])([*])O	0.239	284 out of 338
FCFP_10	-1539132615	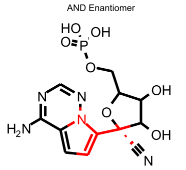 [*]C([*])([*])[c]1:[cH]:[*]:[*]:n:1:[*]	0.224	11 out of 13
FCFP_10	-1043250487	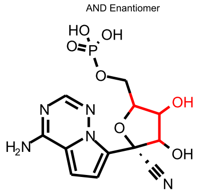 [*]C1[*][*]C([*])C1O	0.22	62 out of 75
**Top Features for negative contribution**				
**Fingerprint**	**Bit/Smiles**	**Feature Structure**	**Score**	**Moderate_Severe in training set**
FCFP_10	4427049	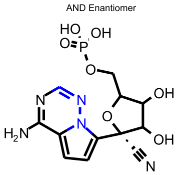 [*]:[cH]:n:n(:[*]):[*]	-1.29	0 out of 4
FCFP_10	-332197802	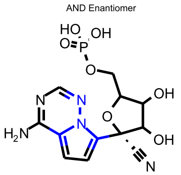 [*][c]1:[*]:[*]:[c](:[*]):n:1:n:[*]	-0.507	0 out of 1

FCFP_10	713358128	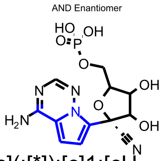 [*][c](:[*]):[c]1:[cH]:[cH]:[c]([*]):n:1:[*]	-0.307	8 out of 17

new dicoumarin.cdx

TOPKAT_Ocular_Irritancy_None_vs_Irritant

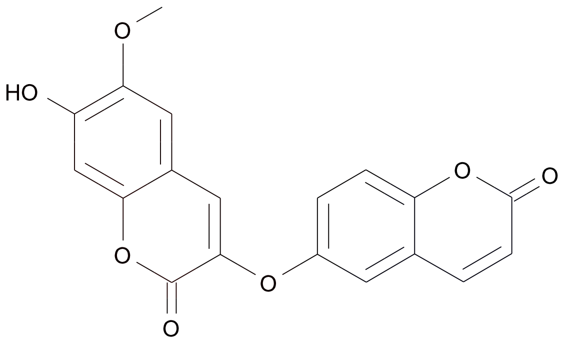

$C_{19}H_{12}O_7$
Molecular Weight: 352.29437
ALogP: 2.975
Rotatable Bonds: 3
Acceptors: 7
Donors: 1

Model Prediction

Prediction: Irritant
Probability: 1
Enrichment: 1.18
Bayesian Score: 0.645
Mahalanobis Distance: 8.38
Mahalanobis Distance p-value: 0.813

Prediction: Positive if the Bayesian score is above the estimated best cutoff value from minimizing the false positive and false negative rate.
Probability: The esimated probability that the sample is in the positive category. This assumes that the Bayesian score follows a normal distribution and is different from the prediction using a cutoff.
Enrichment: An estimate of enrichment, that is, the increased likelihood (versus random) of this sample being in the category.
Bayesian Score: The standard Laplacian-modified Bayesian score.
Mahalanobis Distance: The Mahalanobis distance (MD) is the distance to the center of the training data. The larger the MD, the less trustworthy the prediction.
Mahalanobis Distance p-value: The p-value gives the fraction of training data with an MD greater than or equal to the one for the given sample, assuming normally distributed data. The smaller the p-value, the less trustworthy the prediciton. For highly non-normal X properties (e.g., fingerprints), the MD p-value is wildly inaccurate.

Structural Similar Compounds

Name	COLCHICINE	2;5-DICHLORO-4(3'-METHYL-5' PYRAZOLON-1'-YL)BENZENE SULFONIC ACID	1;8;9-ANTHRACENETRIOL; TRIACETATE
Structure	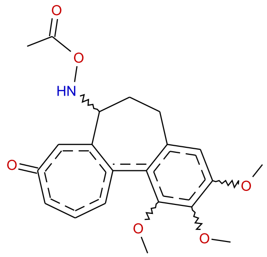	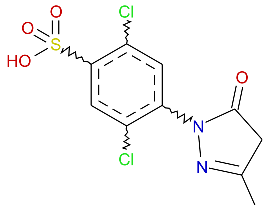	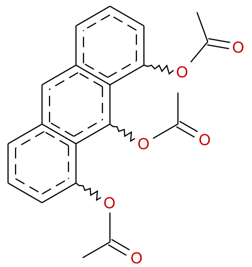
Actual Endpoint	Irritant	Irritant	Irritant
Predicted Endpoint	Irritant	Irritant	Irritant
Distance	0.616	0.697	0.728
Reference	AJOPAA 31;837;48	28ZPAK-;186;72	BJOPAL 53;819;69

Model Applicability

Unknown features are fingerprint features in the query molecule, but not found or appearing too infrequently in the training set.

1. All properties and OPS components are within expected ranges.

Feature Contribution

Top features for positive contribution				
**Fingerprint**	**Bit/Smiles**	**Feature Structure**	**Score**	**Irritant in training set**
FCFP_12	-1979033238	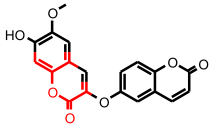 [*]C1=[*][c](:[*]):[c](OC1=O):[cH]:[*]	0.192	10 out of 10

FCFP_12	-1099193755	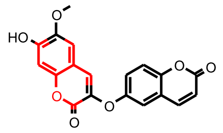 [*][c]1:[*]:[cH]:[c]2 C=[*]C(=[*])O[c]:2:[cH]:1	0.175	5 out of 5
FCFP_12	436915834	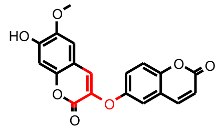 [*]O\C(=C\[*])\C(=[*])[*]	0.167	4 out of 4

Top Features for negative contribution

Fingerprint	Bit/Smiles	Feature Structure	Score	Irritant in training set
FCFP_12	-146015125	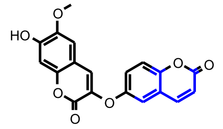 [*]=C1[*][c](:[*]):[c](:[cH]:[*])C=C1	-0.268	1 out of 2
FCFP_12	1673930087	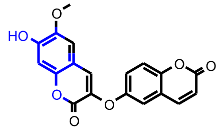 [*]O[c]1:[cH]:[c](O): [c]([*]):[*]:[c]:1[*]	-0.218	5 out of 8
FCFP_12	451371068	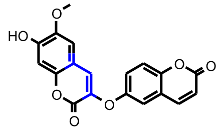 [*]C(=C[c](:[*]):[*]) [*]	-0.167	6 out of 9

remdesivir

TOPKAT_Ocular_Irritancy_None_vs_Irritant

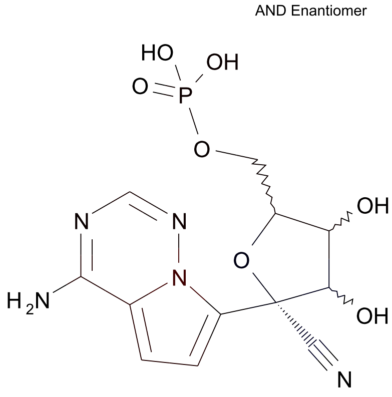

$C_{12}H_{14}N_5O_7P$
Molecular Weight: 371.24262
ALogP: -1.502
Rotatable Bonds: 4
Acceptors: 11
Donors: 5

Structural Similar Compounds

Name	1;3;6-NAPHTHALENE TRISULFONIC ACID;7-AMINO-	Methanol; (s-triazine-2;4;6-triyltrinitrilo)hexa-	2;2'-Biphenyldisulfonic acid; 4;4'-diamino-
Structure	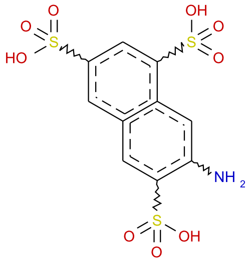	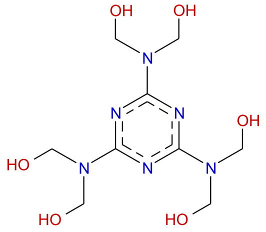	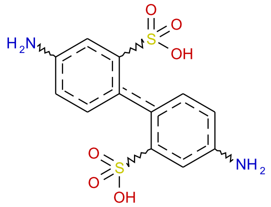
Actual Endpoint	Irritant	Irritant	Irritant
Predicted Endpoint	Irritant	Irritant	Irritant
Distance	0.766	0.795	0.859
Reference	28ZPAK-;190;72	Prehled Prumyslove Toxikologie; Organicke Latky; Marhold; J. -;876;86	Prehled Prumyslove Toxikologie; Organicke Latky; Marhold; J. pp 1061;86

Model Prediction

Prediction: Irritant
Probability: 1
Enrichment: 1.18
Bayesian Score: 1.33
Mahalanobis Distance: 10.7
Mahalanobis Distance p-value: 0.0147

Prediction: Positive if the Bayesian score is above the estimated best cutoff value from minimizing the false positive and false negative rate.
Probability: The esimated probability that the sample is in the positive category. This assumes that the Bayesian score follows a normal distribution and is different from the prediction using a cutoff.
Enrichment: An estimate of enrichment, that is, the increased likelihood (versus random) of this sample being in the category.
Bayesian Score: The standard Laplacian-modified Bayesian score.
Mahalanobis Distance: The Mahalanobis distance (MD) is the distance to the center of the training data. The larger the MD, the less trustworthy the prediction.
Mahalanobis Distance p-value: The p-value gives the fraction of training data with an MD greater than or equal to the one for the given sample, assuming normally distributed data. The smaller the p-value, the less trustworthy the prediciton. For highly non-normal X properties (e.g., fingerprints), the MD p-value is wildly inaccurate.

Model Applicability

Unknown features are fingerprint features in the query molecule, but not found or appearing too infrequently in the training set.

1. All properties and OPS components are within expected ranges.
2. Unknown FCFP_2 feature: 472180098: [*]OP(=O)(O)O
3. Unknown FCFP_2 feature: -124685461: [*]:n:[cH]:n:[*]
4. Unknown FCFP_2 feature: -1151884458: [*]:n:[c](N):[c](:[*]):[*]

Feature Contribution

Top features for positive contribution				
Fingerprint	Bit/Smiles	Feature Structure	Score	Irritant in training set

FCFP_12	1747237384	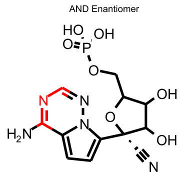 [*][c](:[*]):n:[cH]:[*]	0.208	44 out of 44
FCFP_12	178336375	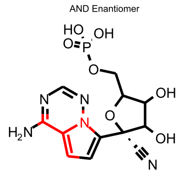 [*][c](:[*]):[c]1:[cH]:[*]:[*]:n:1:[*]	0.202	19 out of 19
FCFP_12	713358128	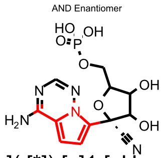 [*][c](:[*]):[c]1:[cH]:[cH]:[c]([*]):n:1:[*]	0.2	17 out of 17

Top Features for negative contribution

Fingerprint	Bit/Smiles	Feature Structure	Score	Irritant in training set
FCFP_12	-836603894	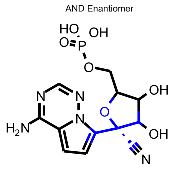 [*]C1[*][*]O[C@]1(C#[*])[c](:[*]):[*]	-0.592	0 out of 1
FCFP_12	-1277879912	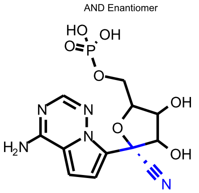 [*]C([*])([*])C#N	-0.0939	33 out of 45

FCFP_12	-1272768868	AND Enantiomer 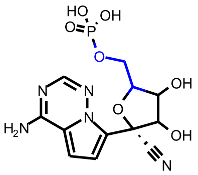 [*]OCC([*])[*]	0	396 out of 514

new dicoumarin.cdx

TOPKAT_Rat_Female_FDA_None_vs_Carcinogen

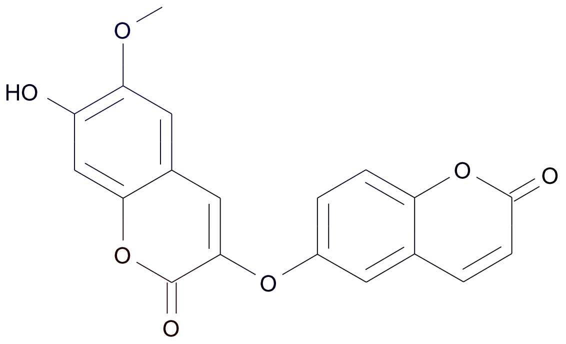

$C_{19}H_{12}O_7$

Molecular Weight: 352.29437

ALogP: 2.975

Rotatable Bonds: 3

Acceptors: 7

Donors: 1

Structural Similar Compounds

Name	Prazocin	Isradipine	Nifedipine
Structure	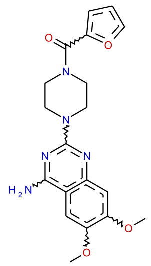	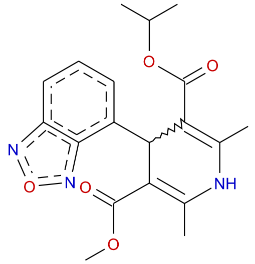	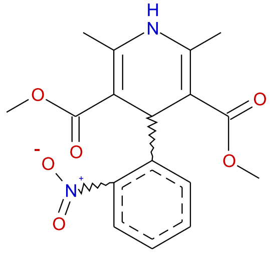
Actual Endpoint	Non-Carcinogen	Non-Carcinogen	Non-Carcinogen
Predicted Endpoint	Non-Carcinogen	Non-Carcinogen	Non-Carcinogen
Distance	0.587	0.605	0.617
Reference	US FDA (Centre for Drug Eval.& Res./Off. Testing & Res.) Sept. 1997	US FDA (Centre for Drug Eval.& Res./Off. Testing & Res.) Sept. 1997	US FDA (Centre for Drug Eval.& Res./Off. Testing & Res.) Sept. 1997

Model Prediction

Prediction: Carcinogen

Probability: 0.284

Enrichment: 0.883

Bayesian Score: -0.721

Mahalanobis Distance: 11.6

Mahalanobis Distance p-value: 0.0154

Prediction: Positive if the Bayesian score is above the estimated best cutoff value from minimizing the false positive and false negative rate.

Probability: The esimated probability that the sample is in the positive category. This assumes that the Bayesian score follows a normal distribution and is different from the prediction using a cutoff.

Enrichment: An estimate of enrichment, that is, the increased likelihood (versus random) of this sample being in the category.

Bayesian Score: The standard Laplacian-modified Bayesian score.

Mahalanobis Distance: The Mahalanobis distance (MD) is the distance to the center of the training data. The larger the MD, the less trustworthy the prediction.

Mahalanobis Distance p-value: The p-value gives the fraction of training data with an MD greater than or equal to the one for the given sample, assuming normally distributed data. The smaller the p-value, the less trustworthy the prediciton. For highly non-normal X properties (e.g., fingerprints), the MD p-value is wildly inaccurate.

Model Applicability

Unknown features are fingerprint features in the query molecule, but not found or appearing too infrequently in the training set.

1. All properties and OPS components are within expected ranges.
2. Unknown ECFP_2 feature: -741588105: [*]O\C(=C\[*])\C(=[*])[*]

Feature Contribution

Top features for positive contribution

Fingerprint	Bit/Smiles	Feature Structure	Score	Carcinogen in training set
ECFP_12	1573945311	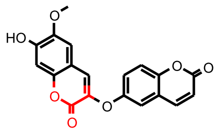 [*]OC(=O)C(=[*])[*]	0.575	3 out of 4

ECFP_12	683445015	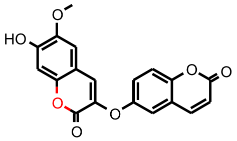 [*]O[*]	0.294	28 out of 66
ECFP_12	1336666212	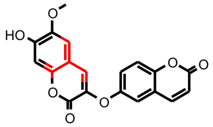 [*][c](:[*]):[c](C=[*]):[cH]:[*]	0.288	2 out of 4
**Top Features for negative contribution**				
**Fingerprint**	**Bit/Smiles**	**Feature Structure**	**Score**	**Carcinogen in training set**
ECFP_12	1408898974	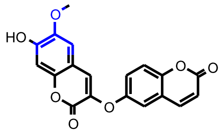 [*]O[c](:[cH]:[*]):[c]([*]):[*]	-0.517	5 out of 29
ECFP_12	1305253718	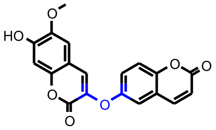 [*]C(=[*])O[c](:[*]): [*]	-0.485	0 out of 2
ECFP_12	1680623188	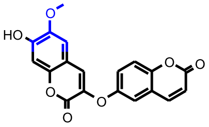 [*][c](:[*]):[c](OC): [cH]:[*]	-0.295	3 out of 14

remdesivir

TOPKAT_Rat_Female_FDA_None_vs_Carcinogen

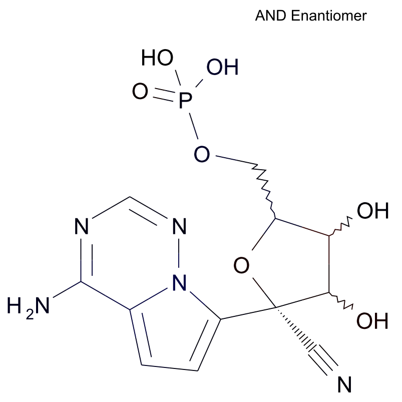

$C_{12}H_{14}N_5O_7P$
Molecular Weight: 371.24262
ALogP: -1.502
Rotatable Bonds: 4
Acceptors: 11
Donors: 5

Model Prediction

Prediction: Non-Carcinogen
Probability: 0.243
Enrichment: 0.756
Bayesian Score: -3.24
Mahalanobis Distance: 13.8
Mahalanobis Distance p-value: 5.04e-006

Prediction: Positive if the Bayesian score is above the estimated best cutoff value from minimizing the false positive and false negative rate.
Probability: The esimated probability that the sample is in the positive category. This assumes that the Bayesian score follows a normal distribution and is different from the prediction using a cutoff.
Enrichment: An estimate of enrichment, that is, the increased likelihood (versus random) of this sample being in the category.
Bayesian Score: The standard Laplacian-modified Bayesian score.
Mahalanobis Distance: The Mahalanobis distance (MD) is the distance to the center of the training data. The larger the MD, the less trustworthy the prediction.
Mahalanobis Distance p-value: The p-value gives the fraction of training data with an MD greater than or equal to the one for the given sample, assuming normally distributed data. The smaller the p-value, the less trustworthy the prediciton. For highly non-normal X properties (e.g., fingerprints), the MD p-value is wildly inaccurate.

Structural Similar Compounds

Name	Streptozocin	Tetracycline	Famotidine
Structure	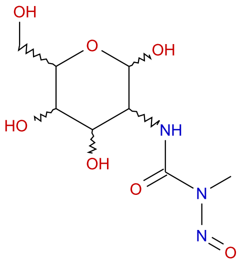	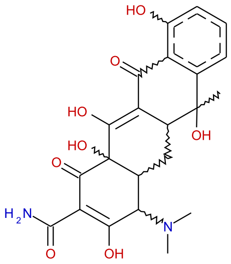	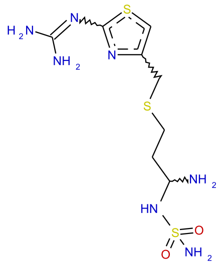
Actual Endpoint	Carcinogen	Non-Carcinogen	Non-Carcinogen
Predicted Endpoint	Carcinogen	Non-Carcinogen	Non-Carcinogen
Distance	0.810	0.858	0.861
Reference	US FDA (Centre for Drug Eval.& Res./Off. Testing & Res.) Sept. 1997	US FDA (Centre for Drug Eval.& Res./Off. Testing & Res.) Sept. 1997	US FDA (Centre for Drug Eval.& Res./Off. Testing & Res.) Sept. 1997

Model Applicability

Unknown features are fingerprint features in the query molecule, but not found or appearing too infrequently in the training set.

1. All properties and OPS components are within expected ranges.
2. Unknown ECFP_2 feature: 1126642748: [*]OP(=O)(O)O
3. Unknown ECFP_2 feature: -1250439909: [*]COP(=[*])([*])[*]
4. Unknown ECFP_2 feature: 1258791451: [*]C1[*][*]O[C@]1(C#[*])[c](:[*]):[*]
5. Unknown ECFP_2 feature: -1507082173: [*][c]1:[*]:[*]:[c](:[*]):n:1:n:[*]
6. Unknown ECFP_2 feature: -66263742: [*]C([*])([*])[c]1:[cH]:[*]:[*]:n:1:[*]

Feature Contribution

Top features for positive contribution				
**Fingerprint**	**Bit/Smiles**	**Feature Structure**	**Score**	**Carcinogen in training set**

ECFP_12	-553149446	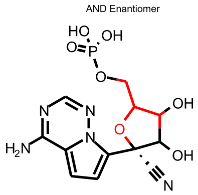 [*]CC1O[*][*]C1[*]	0.575	3 out of 4
ECFP_12	-1114776580	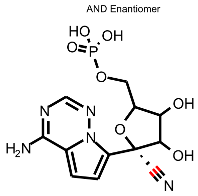 [*]C#[*]	0.461	10 out of 19
ECFP_12	-521596699	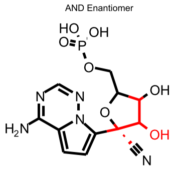 [*]C1[*][*]C([*])([*])C1O	0.445	3 out of 5
**Top Features for negative contribution**				
**Fingerprint**	**Bit/Smiles**	**Feature Structure**	**Score**	**Carcinogen in training set**
ECFP_12	-1687549011	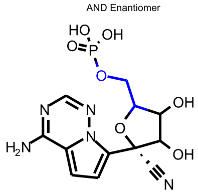 [*]OCC([*])[*]	-0.661	0 out of 3
ECFP_12	2024329577	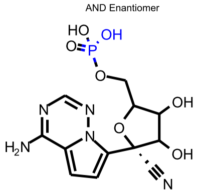 [*]P(=[*])([*])O	-0.661	0 out of 3

ECFP_12	-1734834311	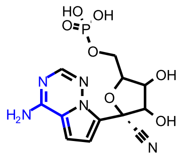 [*]:n:[c](N):[c](:[*]):[*]	-0.56	1 out of 8

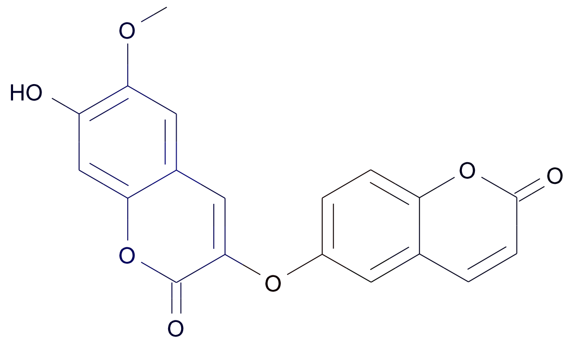

$C_{19}H_{12}O_7$
Molecular Weight: 352.29437
ALogP: 2.975
Rotatable Bonds: 3
Acceptors: 7
Donors: 1

Model Prediction

Prediction: Single-Carcinogen
Probability: 0.383
Enrichment: 1.02
Bayesian Score: -3.51
Mahalanobis Distance: 15.4
Mahalanobis Distance p-value: 1.35e-006

Prediction: Positive if the Bayesian score is above the estimated best cutoff value from minimizing the false positive and false negative rate.
Probability: The esimated probability that the sample is in the positive category. This assumes that the Bayesian score follows a normal distribution and is different from the prediction using a cutoff.
Enrichment: An estimate of enrichment, that is, the increased likelihood (versus random) of this sample being in the category.
Bayesian Score: The standard Laplacian-modified Bayesian score.
Mahalanobis Distance: The Mahalanobis distance (MD) is the distance to the center of the training data. The larger the MD, the less trustworthy the prediction.
Mahalanobis Distance p-value: The p-value gives the fraction of training data with an MD greater than or equal to the one for the given sample, assuming normally distributed data. The smaller the p-value, the less trustworthy the prediciton. For highly non-normal X properties (e.g., fingerprints), the MD p-value is wildly inaccurate.

Structural Similar Compounds

Name	Griseofulvin	Estrogens; conjug.	Omeprazole
Structure	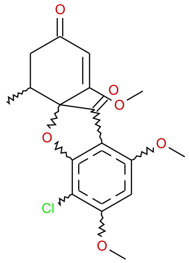	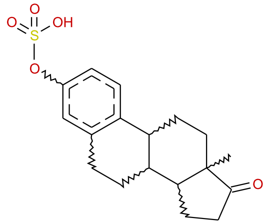	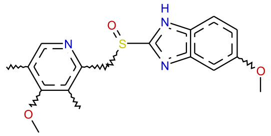
Actual Endpoint	Single-Carcinogen	Single-Carcinogen	Multiple-Carcinogen
Predicted Endpoint	Single-Carcinogen	Single-Carcinogen	Multiple-Carcinogen
Distance	0.612	0.647	0.651
Reference	US FDA (Centre for Drug Eval.& Res./Off. Testing & Res.) Sept. 1997	US FDA (Centre for Drug Eval.& Res./Off. Testing & Res.) Sept. 1997	US FDA (Centre for Drug Eval.& Res./Off. Testing & Res.) Sept. 1997

Model Applicability

Unknown features are fingerprint features in the query molecule, but not found or appearing too infrequently in the training set.

1. All properties and OPS components are within expected ranges.

Feature Contribution

Top features for positive contribution

Fingerprint	Bit/Smiles	Feature Structure	Score	Multiple-Carcinogen in training set
SCFP_4	-1971196727	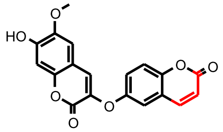 [*]\C=C/C(=[*])[*]	0.295	5 out of 11

SCFP_4	-1374800107	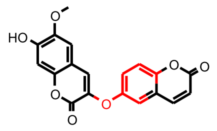 [*]O[c]1:[cH]:[*]:[c]([*]):[cH]:[cH]:1	0.288	10 out of 23
SCFP_4	-424425761	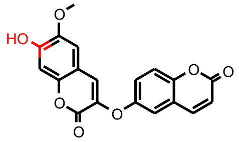 [*]:[c](:[*])O	0.201	6 out of 15
**Top Features for negative contribution**				
**Fingerprint**	**Bit/Smiles**	**Feature Structure**	**Score**	**Multiple-Carcinogen in training set**
SCFP_4	1132907712	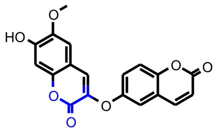 [*]OC(=O)C(=[*])[*]	-0.651	1 out of 9
SCFP_4	392579710	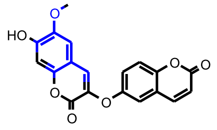 [*]O[c]1:[cH]:[c](C=[*]):[c]([*]):[*]:[c]:1[*]	-0.489	0 out of 2
SCFP_4	130348166	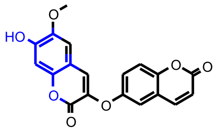 [*]O[c]1:[cH]:[c](O):[c]([*]):[*]:[c]:1[*]	-0.489	0 out of 2

new dicoumarin.cdx

TOPKAT_Rat_Male_FDA_None_vs_Carcinogen

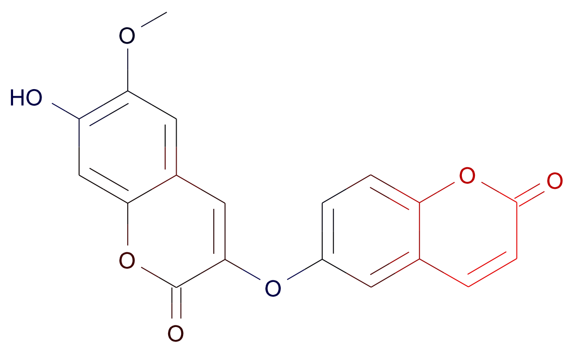

$C_{19}H_{12}O_7$
Molecular Weight: 352.29437
ALogP: 2.975
Rotatable Bonds: 3
Acceptors: 7
Donors: 1

Model Prediction

Prediction: Carcinogen
Probability: 0.495
Enrichment: 1.48
Bayesian Score: 4.21
Mahalanobis Distance: 13.1
Mahalanobis Distance p-value: 0.00122

Prediction: Positive if the Bayesian score is above the estimated best cutoff value from minimizing the false positive and false negative rate.
Probability: The esimated probability that the sample is in the positive category. This assumes that the Bayesian score follows a normal distribution and is different from the prediction using a cutoff.
Enrichment: An estimate of enrichment, that is, the increased likelihood (versus random) of this sample being in the category.
Bayesian Score: The standard Laplacian-modified Bayesian score.
Mahalanobis Distance: The Mahalanobis distance (MD) is the distance to the center of the training data. The larger the MD, the less trustworthy the prediction.
Mahalanobis Distance p-value: The p-value gives the fraction of training data with an MD greater than or equal to the one for the given sample, assuming normally distributed data. The smaller the p-value, the less trustworthy the prediciton. For highly non-normal X properties (e.g., fingerprints), the MD p-value is wildly inaccurate.

Structural Similar Compounds

Name	Prazocin	Griseofulvin	Terazosin
Structure	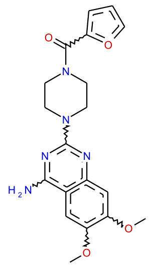	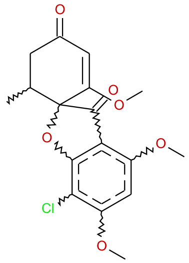	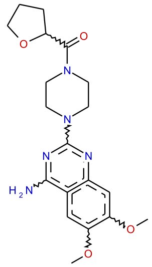
Actual Endpoint	Non-Carcinogen	Carcinogen	Carcinogen
Predicted Endpoint	Non-Carcinogen	Carcinogen	Carcinogen
Distance	0.562	0.624	0.642
Reference	US FDA (Centre for Drug Eval.& Res./Off. Testing & Res.) Sept. 1997	US FDA (Centre for Drug Eval.& Res./Off. Testing & Res.) Sept. 1997	US FDA (Centre for Drug Eval.& Res./Off. Testing & Res.) Sept. 1997

Model Applicability

Unknown features are fingerprint features in the query molecule, but not found or appearing too infrequently in the training set.

1. All properties and OPS components are within expected ranges.

Feature Contribution

Top features for positive contribution

Fingerprint	Bit/Smiles	Feature Structure	Score	Carcinogen in training set
SCFP_6	997923762	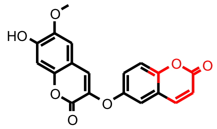 [*]:[c]1:[*]C=CC(=O)O 1	0.603	2 out of 2

SCFP_6	516730200	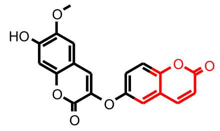 [*]:[cH]:[c]1C=CC(=O)O[c]:1:[*]	0.603	2 out of 2
SCFP_6	2109620068	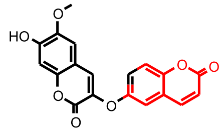 [*][c]1:[*]:[cH]:[c]2OC(=O)C=C[c]:2:[cH]:1	0.603	2 out of 2
**Top Features for negative contribution**				
**Fingerprint**	**Bit/Smiles**	**Feature Structure**	**Score**	**Carcinogen in training set**
SCFP_6	2116304939	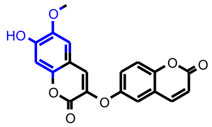 [*]O[c]1:[cH]:[*]:[c]([*]):[cH]:[c]:1O	-0.825	0 out of 4
SCFP_6	-609499983	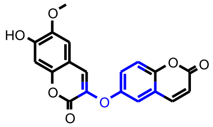 [*]C(=[*])O[c]1:[cH]:[cH]:[*]:[c]([*]):[cH]:1	-0.496	0 out of 2
SCFP_6	1264071919	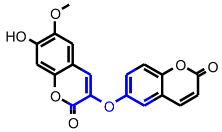 [*]\C=C(\O[c](:[cH]:[*]):[cH]:[*])/C(=[*])[*]	-0.496	0 out of 2

remdesivir

TOPKAT_Rat_Male_FDA_None_vs_Carcinogen

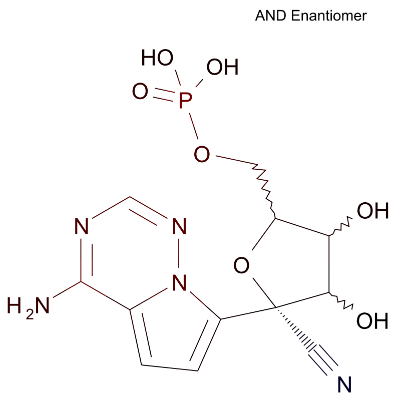

$C_{12}H_{14}N_5O_7P$
Molecular Weight: 371.24262
ALogP: -1.502
Rotatable Bonds: 4
Acceptors: 11
Donors: 5

Model Prediction

Prediction: Carcinogen
Probability: 0.481
Enrichment: 1.44
Bayesian Score: 3.82
Mahalanobis Distance: 14.1
Mahalanobis Distance p-value: 3.32e-005

Prediction: Positive if the Bayesian score is above the estimated best cutoff value from minimizing the false positive and false negative rate.
Probability: The esimated probability that the sample is in the positive category. This assumes that the Bayesian score follows a normal distribution and is different from the prediction using a cutoff.
Enrichment: An estimate of enrichment, that is, the increased likelihood (versus random) of this sample being in the category.
Bayesian Score: The standard Laplacian-modified Bayesian score.
Mahalanobis Distance: The Mahalanobis distance (MD) is the distance to the center of the training data. The larger the MD, the less trustworthy the prediction.
Mahalanobis Distance p-value: The p-value gives the fraction of training data with an MD greater than or equal to the one for the given sample, assuming normally distributed data. The smaller the p-value, the less trustworthy the prediciton. For highly non-normal X properties (e.g., fingerprints), the MD p-value is wildly inaccurate.

Structural Similar Compounds

Name	Streptozocin	Famotidine	Tetracycline
Structure	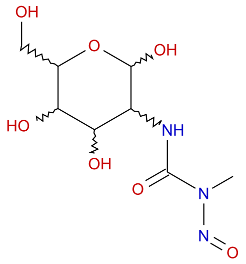	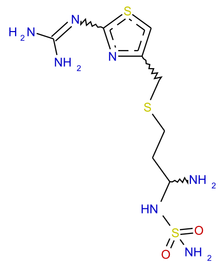	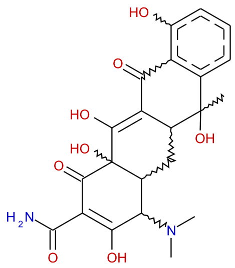
Actual Endpoint	Carcinogen	Non-Carcinogen	Non-Carcinogen
Predicted Endpoint	Carcinogen	Non-Carcinogen	Non-Carcinogen
Distance	0.789	0.850	0.856
Reference	US FDA (Centre for Drug Eval.& Res./Off. Testing & Res.) Sept. 1997	US FDA (Centre for Drug Eval.& Res./Off. Testing & Res.) Sept. 1997	US FDA (Centre for Drug Eval.& Res./Off. Testing & Res.) Sept. 1997

Model Applicability

Unknown features are fingerprint features in the query molecule, but not found or appearing too infrequently in the training set.

1. All properties and OPS components are within expected ranges.

Feature Contribution

Top features for positive contribution

Fingerprint	Bit/Smiles	Feature Structure	Score	Carcinogen in training set
SCFP_6	-1029620989	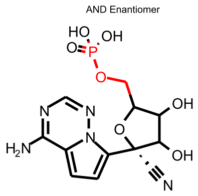 [*]COP(=[*])([*])[*]	0.712	3 out of 3

SCFP_6	1245795878	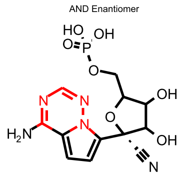 [*][c]1:[*]:n(:[*]):n :[cH]:n:1	0.603	2 out of 2
SCFP_6	149212520	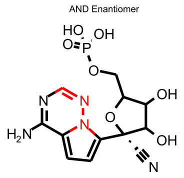 [*]:[cH]:n:n(:[*]):[*]	0.543	9 out of 15
**Top Features for negative contribution**				
**Fingerprint**	**Bit/Smiles**	**Feature Structure**	**Score**	**Carcinogen in training set**
SCFP_6	-1019297400	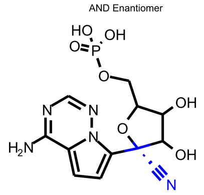 [*]C([*])([*])C#N	-0.674	0 out of 3
SCFP_6	194135988	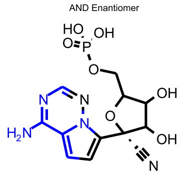 N[c]1:n:[cH]:[*]:n2:[*]:[*]:[cH]:[c]:1:2	-0.278	0 out of 1
SCFP_6	-424515134	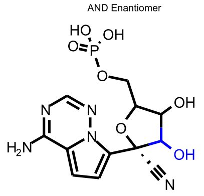 [*]C([*])O	-0.157	30 out of 110

new dicoumarin.cdx

TOPKAT_Rat_Male_FDA_Single_vs_Multiple

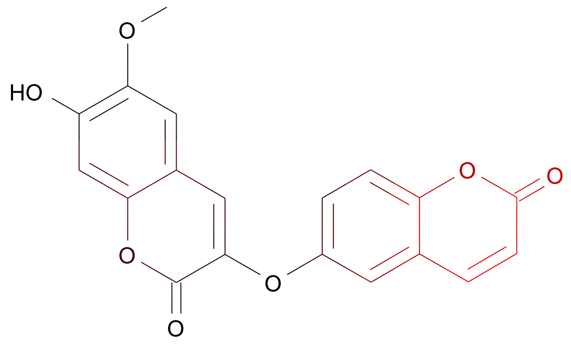

$C_{19}H_{12}O_7$
Molecular Weight: 352.29437
ALogP: 2.975
Rotatable Bonds: 3
Acceptors: 7
Donors: 1

Model Prediction

Prediction: Multiple-Carcinogen
Probability: 0.588
Enrichment: 1.42
Bayesian Score: 5.23
Mahalanobis Distance: 19.4
Mahalanobis Distance p-value: 1.03e-009

Prediction: Positive if the Bayesian score is above the estimated best cutoff value from minimizing the false positive and false negative rate.
Probability: The esimated probability that the sample is in the positive category. This assumes that the Bayesian score follows a normal distribution and is different from the prediction using a cutoff.
Enrichment: An estimate of enrichment, that is, the increased likelihood (versus random) of this sample being in the category.
Bayesian Score: The standard Laplacian-modified Bayesian score.
Mahalanobis Distance: The Mahalanobis distance (MD) is the distance to the center of the training data. The larger the MD, the less trustworthy the prediction.
Mahalanobis Distance p-value: The p-value gives the fraction of training data with an MD greater than or equal to the one for the given sample, assuming normally distributed data. The smaller the p-value, the less trustworthy the prediciton. For highly non-normal X properties (e.g., fingerprints), the MD p-value is wildly inaccurate.

Structural Similar Compounds

Name	Terazosin	Griseofulvin	Isradipine
Structure	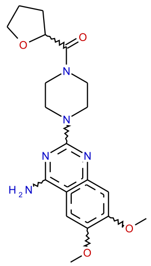	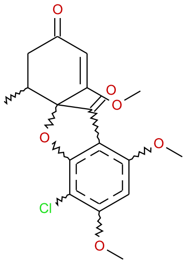	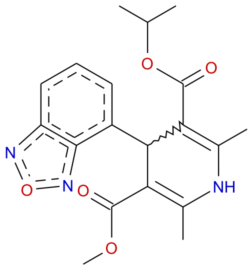
Actual Endpoint	Single-Carcinogen	Single-Carcinogen	Single-Carcinogen
Predicted Endpoint	Single-Carcinogen	Single-Carcinogen	Single-Carcinogen
Distance	0.669	0.674	0.681
Reference	US FDA (Centre for Drug Eval.& Res./Off. Testing & Res.) Sept. 1997	US FDA (Centre for Drug Eval.& Res./Off. Testing & Res.) Sept. 1997	US FDA (Centre for Drug Eval.& Res./Off. Testing & Res.) Sept. 1997

Model Applicability

Unknown features are fingerprint features in the query molecule, but not found or appearing too infrequently in the training set.

1. All properties and OPS components are within expected ranges.

Feature Contribution

Top features for positive contribution

Fingerprint	Bit/Smiles	Feature Structure	Score	Multiple-Carcinogen in training set
SCFP_8	-1977229858	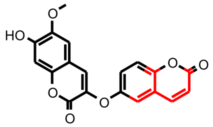 [*]=C1[*][c](:[*]):[c](:[cH]:[*])C=C1	0.553	2 out of 2

SCFP_8	966282057	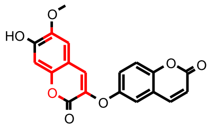 [*]C1=C[c]2:[cH]:[c]([*]):[*]:[cH]:[c]:2O [*]1	0.553	2 out of 2
SCFP_8	-392286499	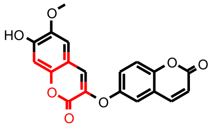 [*]C1=[*][c](:[*]):[c](OC1=O):[cH]:[*]	0.553	2 out of 2

Top Features for negative contribution

Fingerprint	Bit/Smiles	Feature Structure	Score	Multiple-Carcinogen in training set
SCFP_8	1132907712	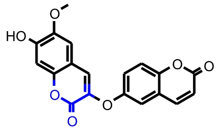 [*]OC(=O)C(=[*])[*]	-0.58	2 out of 12
SCFP_8	136239834	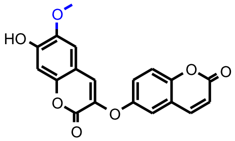 [*]OC	-0.358	3 out of 13
SCFP_8	616576836	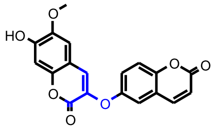 [*]O\C(=C\[*])\C(=[*])[*]	-0.31	0 out of 1

remdesivir

TOPKAT_Rat_Male_FDA_Single_vs_Multiple

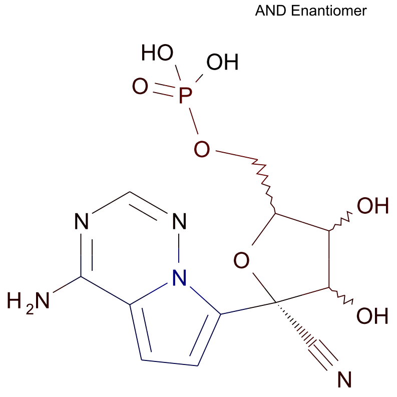

$C_{12}H_{14}N_5O_7P$
Molecular Weight: 371.24262
ALogP: -1.502
Rotatable Bonds: 4
Acceptors: 11
Donors: 5

Model Prediction

Prediction: Multiple-Carcinogen
Probability: 0.556
Enrichment: 1.34
Bayesian Score: 3.52
Mahalanobis Distance: 14
Mahalanobis Distance p-value: 8.72e-005

Prediction: Positive if the Bayesian score is above the estimated best cutoff value from minimizing the false positive and false negative rate.
Probability: The esimated probability that the sample is in the positive category. This assumes that the Bayesian score follows a normal distribution and is different from the prediction using a cutoff.
Enrichment: An estimate of enrichment, that is, the increased likelihood (versus random) of this sample being in the category.
Bayesian Score: The standard Laplacian-modified Bayesian score.
Mahalanobis Distance: The Mahalanobis distance (MD) is the distance to the center of the training data. The larger the MD, the less trustworthy the prediction.
Mahalanobis Distance p-value: The p-value gives the fraction of training data with an MD greater than or equal to the one for the given sample, assuming normally distributed data. The smaller the p-value, the less trustworthy the prediciton. For highly non-normal X properties (e.g., fingerprints), the MD p-value is wildly inaccurate.

Structural Similar Compounds

Name	Streptozocin	Minocycline	Ribavirin
Structure	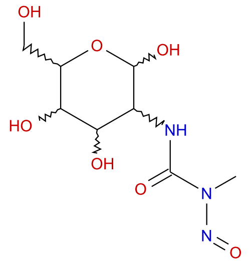	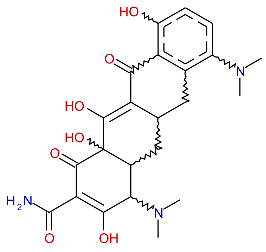	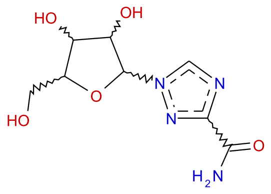
Actual Endpoint	Multiple-Carcinogen	Single-Carcinogen	Multiple-Carcinogen
Predicted Endpoint	Multiple-Carcinogen	Single-Carcinogen	Multiple-Carcinogen
Distance	0.817	0.908	0.929
Reference	US FDA (Centre for Drug Eval.& Res./Off. Testing & Res.) Sept. 1997	US FDA (Centre for Drug Eval.& Res./Off. Testing & Res.) Sept. 1997	US FDA (Centre for Drug Eval.& Res./Off. Testing & Res.) Sept. 1997

Model Applicability

Unknown features are fingerprint features in the query molecule, but not found or appearing too infrequently in the training set.

1. Num_H_Acceptors out of range. Value: 11. Training min, max, mean, SD: 0, 9, 3.8906, 2.196.

Feature Contribution

Top features for positive contribution				
**Fingerprint**	**Bit/Smiles**	**Feature Structure**	**Score**	**Multiple-Carcinogen in training set**
SCFP_8	-1029620989	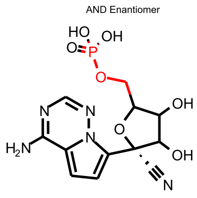 [*]COP(=[*])([*])[*]	0.649	3 out of 3

SCFP_8	2	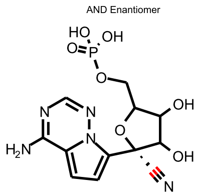 [*]C#[*]	0.584	6 out of 8
SCFP_8	-553385769	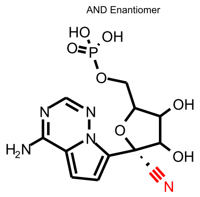 [*]C#N	0.553	2 out of 2
**Top Features for negative contribution**				
**Fingerprint**	**Bit/Smiles**	**Feature Structure**	**Score**	**Multiple-Carcinogen in training set**
SCFP_8	-1381862798	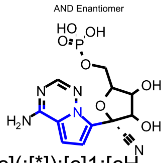 [*][c](:[*]):[c]1:[cH]:[cH]:[c]([*]):n:1:[*]	-0.572	1 out of 7
SCFP_8	1245795878	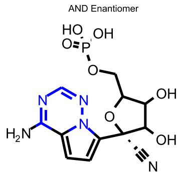 [*][c]1:[*]:n(:[*]):n:[cH]:n:1	-0.546	0 out of 2
SCFP_8	-1375522316	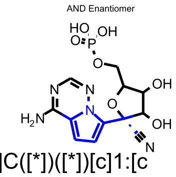 [*]C([*])([*])[c]1:[cH]:[cH]:[c](:[*]):n:1:[*]	-0.546	0 out of 2

new dicoumarin.cdx

TOPKAT_Skin_Irritancy_Mild_vs_Moderate_Severe

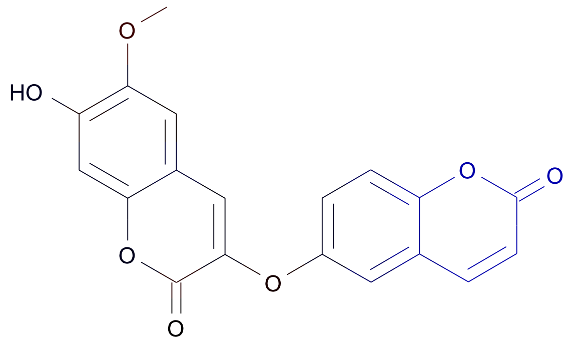

$C_{19}H_{12}O_7$
Molecular Weight: 352.29437
ALogP: 2.975
Rotatable Bonds: 3
Acceptors: 7
Donors: 1

Model Prediction

Prediction: Mild
Probability: 0.174
Enrichment: 0.471
Bayesian Score: -5.89
Mahalanobis Distance: 9.49
Mahalanobis Distance p-value: 0.11

Prediction: Positive if the Bayesian score is above the estimated best cutoff value from minimizing the false positive and false negative rate.
Probability: The esimated probability that the sample is in the positive category. This assumes that the Bayesian score follows a normal distribution and is different from the prediction using a cutoff.
Enrichment: An estimate of enrichment, that is, the increased likelihood (versus random) of this sample being in the category.
Bayesian Score: The standard Laplacian-modified Bayesian score.
Mahalanobis Distance: The Mahalanobis distance (MD) is the distance to the center of the training data. The larger the MD, the less trustworthy the prediction.
Mahalanobis Distance p-value: The p-value gives the fraction of training data with an MD greater than or equal to the one for the given sample, assuming normally distributed data. The smaller the p-value, the less trustworthy the prediciton. For highly non-normal X properties (e.g., fingerprints), the MD p-value is wildly inaccurate.

Structural Similar Compounds

Name	Pregna-1,4-diene-3,20-dione, 21-(acetyloxy)-11-hydroxy-6-methyl-17- (1-oxopropoxy)-, (6-alpha,11-beta)-	1,4-Pentadien-3-one, 1,5-bis(p-azidophenyl)-	Phosphorothioic acid, O,O-dimethyl O-(3,5,6-trichloro-2-pyridyl) es ter
Structure	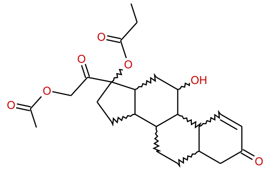	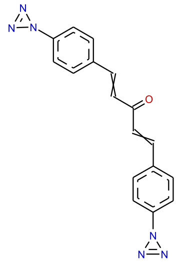	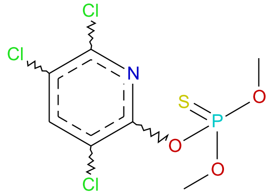
Actual Endpoint	Mild	Mild	Mild
Predicted Endpoint	Mild	Mild	Mild
Distance	0.752	0.767	0.774
Reference	YACHDS Yakuri to Chiryo. Pharmacology and Therapeutics. (Raifu Saiensu Shup pan K.K., 2-5-13, Yaesu, Chuo-ku, Tokyo 104, Japan) V.1- 1972- Volume(issue) /page/year: 19,3103,1991	85JCAE "Prehled Prumyslove Toxikologie; Organicke Latky," Marhold, J., Prague , Czechoslovakia, Avicenum, 1986 Volume(issue)/page/year: -,733,1986	TXAPA9 Toxicology and Applied Pharmacology. (Academic Press, Inc., 1 E. Firs t St., Duluth, MN 55802) V.1- 1959- Volume(issue)/page/year: 21,369,1972

Model Applicability

Unknown features are fingerprint features in the query molecule, but not found or appearing too infrequently in the training set.

1. All properties and OPS components are within expected ranges.

Feature Contribution

Top features for positive contribution

Fingerprint	Bit/Smiles	Feature Structure	Score	Moderate_Severe in training set

FCFP_12	-1977641857	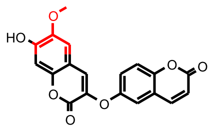 [*][c](:[*]):[c](OC):[cH]:[*]	0.416	18 out of 32
FCFP_12	136627117	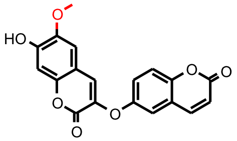 [*]OC	0.361	47 out of 90
FCFP_12	-1099193755	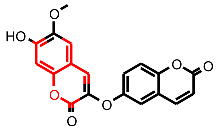 [*][c]1:[*]:[cH]:[c]2C=[*]C(=[*])O[c]:2:[cH]:1	0.343	6 out of 11
**Top Features for negative contribution**				
**Fingerprint**	**Bit/Smiles**	**Feature Structure**	**Score**	**Moderate_Severe in training set**
FCFP_12	-395337223	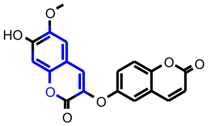 [*]C1=C[c]2:[cH]:[c]([*]):[*]:[cH]:[c]:2O[*]1	-0.733	0 out of 3
FCFP_12	2140586096	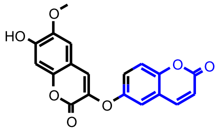 [*][c]1:[*]:[cH]:[c]2OC(=O)C=C[c]:2:[cH]:1	-0.543	0 out of 2

FCFP_12	-938580853	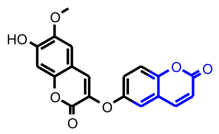 [*]:[cH]:[c]1C=CC(=O)O[c]:1:[*]	-0.543	0 out of 2

remdesivir **TOPKAT_Skin_Irritancy_Mild_vs_Moderate_Severe**

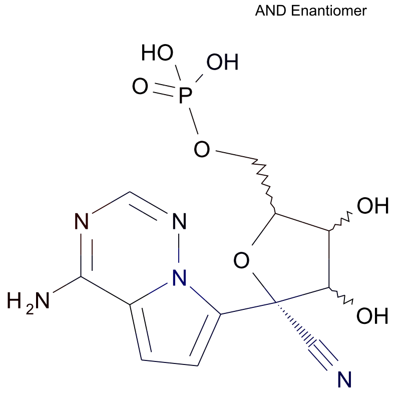

$C_{12}H_{14}N_5O_7P$
Molecular Weight: 371.24262
ALogP: -1.502
Rotatable Bonds: 4
Acceptors: 11
Donors: 5

Structural Similar Compounds

Name	1,3,6-Naphthalenetrisulfonic acid, 7-amino-	2,7-Anthracenedisulfonic acid, 9,10-dihydro-4,5-diamino-9,10-dioxo- 1-hydroxy-, disodium salt	1,5-Naphthalenedisulfonic acid, 2-amino-
Structure	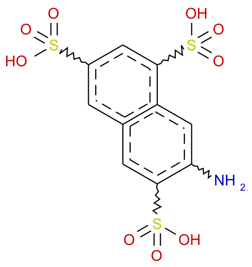	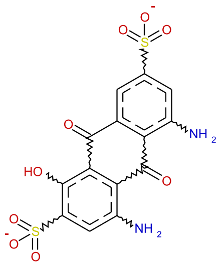	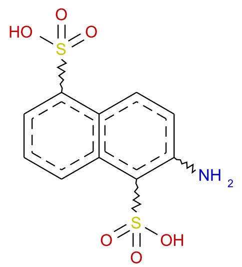
Actual Endpoint	Mild	Mild	Mild
Predicted Endpoint	Mild	Mild	Mild
Distance	0.759	1.033	1.137
Reference	85JCAE "Prehled Prumyslove Toxikologie; Organicke Latky," Marhold, J., Prague , Czechoslovakia, Avicenum, 1986 Volume(issue)/page/year: -,1058,1986	28ZPAK "Sbornik Vysledku Toxixologickeho Vysetreni Latek A Pripravku," Marhold d, J.V., Institut Pro Vychovu Vedoucicn Pracovniku Chemickeho Prumyclu Praha, Cz echoslovakia, 1972 Volume(issue)/page/year: -,239,1	85JCAE "Prehled Prumyslove Toxikologie; Organicke Latky," Marhold, J., Prague , Czechoslovakia, Avicenum, 1986 Volume(issue)/page/year: -,1058,1986

Model Prediction

Prediction: Mild
Probability: 0.0911
Enrichment: 0.247
Bayesian Score: -8.73
Mahalanobis Distance: 13.5
Mahalanobis Distance p-value: 1.21e-009

Prediction: Positive if the Bayesian score is above the estimated best cutoff value from minimizing the false positive and false negative rate.
Probability: The esimated probability that the sample is in the positive category. This assumes that the Bayesian score follows a normal distribution and is different from the prediction using a cutoff.
Enrichment: An estimate of enrichment, that is, the increased likelihood (versus random) of this sample being in the category.
Bayesian Score: The standard Laplacian-modified Bayesian score.
Mahalanobis Distance: The Mahalanobis distance (MD) is the distance to the center of the training data. The larger the MD, the less trustworthy the prediction.
Mahalanobis Distance p-value: The p-value gives the fraction of training data with an MD greater than or equal to the one for the given sample, assuming normally distributed data. The smaller the p-value, the less trustworthy the prediciton. For highly non-normal X properties (e.g., fingerprints), the MD p-value is wildly inaccurate.

Model Applicability

Unknown features are fingerprint features in the query molecule, but not found or appearing too infrequently in the training set.

1. All properties and OPS components are within expected ranges.
2. Unknown FCFP_2 feature: 472180098: [*]OP(=O)(O)O
3. Unknown FCFP_2 feature: -332197802: [*][c]1:[*]:[*]:[c](:[*]):n:1:n:[*]

Feature Contribution

Top features for positive contribution

Fingerprint	Bit/Smiles	Feature Structure	Score	Moderate_Severe in training set

FCFP_12	76292238	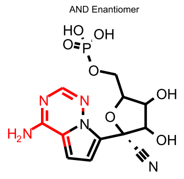 [*]:[c]1:[*]:n:[cH]:n:[c]:1N	0.385	1 out of 1
FCFP_12	-1151884458	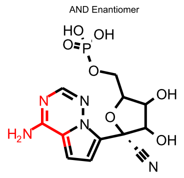 [*]:n:[c](N):[c](:[*]):[*]	0.385	1 out of 1
FCFP_12	-124685461	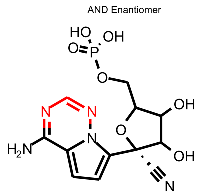 [*]:n:[cH]:n:[*]	0.206	2 out of 4

Top Features for negative contribution

Fingerprint	Bit/Smiles	Feature Structure	Score	Moderate_Severe in training set
FCFP_12	4427049	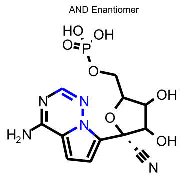 [*]:[cH]:n:n(:[*]):[*]	-0.893	0 out of 4
FCFP_12	-1277879912	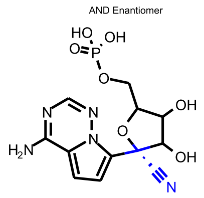 [*]C([*])([*])C#N	-0.548	5 out of 26

FCFP_12	-836603894	AND Enantiomer<br>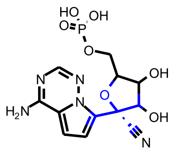<br>[*]C1[*][*]O[C@]1(C#[*])[c](:[*]):[*]	-0.543	0 out of 2

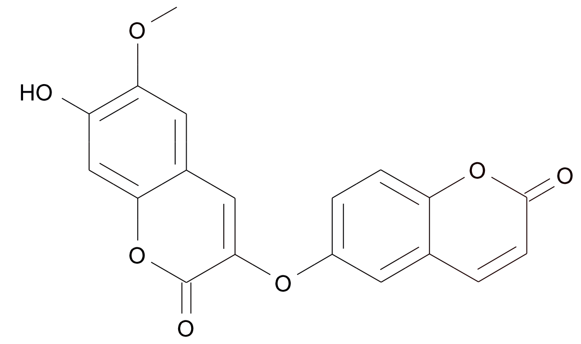

$C_{19}H_{12}O_7$

Molecular Weight: 352.29437

ALogP: 2.975

Rotatable Bonds: 3

Acceptors: 7

Donors: 1

Structural Similar Compounds

Name	Pregna-1,4-diene-3,20-dione, 21-(acetyloxy)-11-hydroxy-6-methyl-17- (1-oxopropoxy)-, (6-alpha,11-beta)-	1,4-Pentadien-3-one, 1,5-bis(p-azidophenyl)-	Phosphorothioic acid, O,O-dimethyl O-(3,5,6-trichloro-2-pyridyl) es ter
Structure	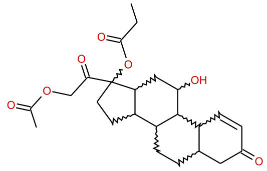	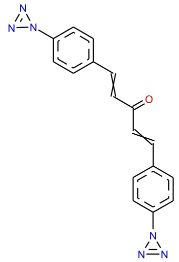	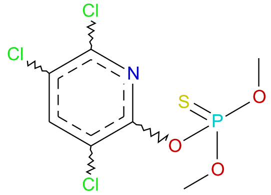
Actual Endpoint	Irritant	Irritant	Irritant
Predicted Endpoint	Irritant	Irritant	Irritant
Distance	0.760	0.766	0.777
Reference	YACHDS Yakuri to Chiryo. Pharmacology and Therapeutics. (Raifu Saiensu Shup pan K.K., 2-5-13, Yaesu, Chuo-ku, Tokyo 104, Japan) V.1- 1972- Volume(issue) /page/year: 19,3103,1991	85JCAE "Prehled Prumyslove Toxikologie; Organicke Latky," Marhold, J., Prague , Czechoslovakia, Avicenum, 1986 Volume(issue)/page/year: -,733,1986	TXAPA9 Toxicology and Applied Pharmacology. (Academic Press, Inc., 1 E. Firs t St., Duluth, MN 55802) V.1- 1959- Volume(issue)/page/year: 21,369,1972

Model Prediction

Prediction: Irritant

Probability: 0.995

Enrichment: 1.08

Bayesian Score: -0.0259

Mahalanobis Distance: 9.01

Mahalanobis Distance p-value: 0.416

Prediction: Positive if the Bayesian score is above the estimated best cutoff value from minimizing the false positive and false negative rate.
Probability: The esimated probability that the sample is in the positive category. This assumes that the Bayesian score follows a normal distribution and is different from the prediction using a cutoff.
Enrichment: An estimate of enrichment, that is, the increased likelihood (versus random) of this sample being in the category.
Bayesian Score: The standard Laplacian-modified Bayesian score.
Mahalanobis Distance: The Mahalanobis distance (MD) is the distance to the center of the training data. The larger the MD, the less trustworthy the prediction.
Mahalanobis Distance p-value: The p-value gives the fraction of training data with an MD greater than or equal to the one for the given sample, assuming normally distributed data. The smaller the p-value, the less trustworthy the prediciton. For highly non-normal X properties (e.g., fingerprints), the MD p-value is wildly inaccurate.

Model Applicability

Unknown features are fingerprint features in the query molecule, but not found or appearing too infrequently in the training set.

1. All properties and OPS components are within expected ranges.

Feature Contribution

Top features for positive contribution

Fingerprint	Bit/Smiles	Feature Structure	Score	Irritant in training set

FCFP_12	-146015125	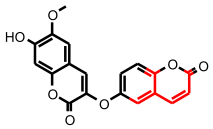 [*]=C1[*][c](:[*]):[c](:[cH]:[*])C=C1	0.085	24 out of 24
FCFP_12	-1979033238	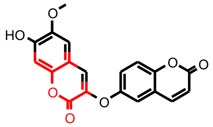 [*]C1=[*][c](:[*]):[c](OC1=O):[cH]:[*]	0.0841	19 out of 19
FCFP_12	-2132692875	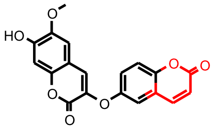 [*]:[c]1:[*]OC(=O)C=C 1	0.0756	6 out of 6

Top Features for negative contribution

Fingerprint	Bit/Smiles	Feature Structure	Score	Irritant in training set
FCFP_12	7	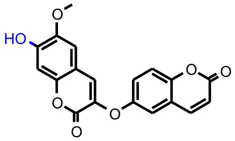 [*]O	-0.118	104 out of 128
FCFP_12	74595001	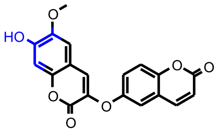 [*][c](:[*]):[c](O):[cH]:[*]	-0.11	54 out of 66

FCFP_12	-549108873	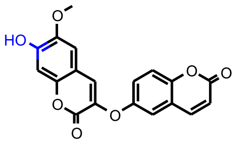 [*]:[c](:[*])O	-0.11	54 out of 66

remdesivir

TOPKAT_Skin_Irritancy_None_vs_Irritant

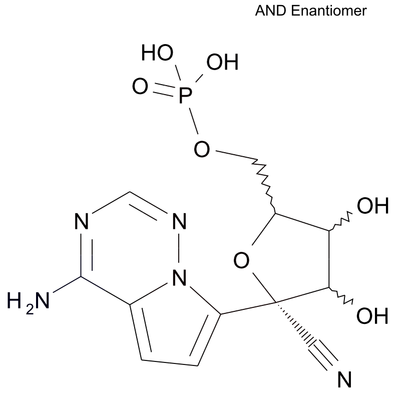

$C_{12}H_{14}N_5O_7P$

Molecular Weight: 371.24262

ALogP: -1.502

Rotatable Bonds: 4

Acceptors: 11

Donors: 5

Model Prediction

Prediction: Irritant

Probability: 0.976

Enrichment: 1.06

Bayesian Score: -0.492

Mahalanobis Distance: 13.2

Mahalanobis Distance p-value: 3.18e-008

Prediction: Positive if the Bayesian score is above the estimated best cutoff value from minimizing the false positive and false negative rate.
Probability: The esimated probability that the sample is in the positive category. This assumes that the Bayesian score follows a normal distribution and is different from the prediction using a cutoff.
Enrichment: An estimate of enrichment, that is, the increased likelihood (versus random) of this sample being in the category.
Bayesian Score: The standard Laplacian-modified Bayesian score.
Mahalanobis Distance: The Mahalanobis distance (MD) is the distance to the center of the training data. The larger the MD, the less trustworthy the prediction.
Mahalanobis Distance p-value: The p-value gives the fraction of training data with an MD greater than or equal to the one for the given sample, assuming normally distributed data. The smaller the p-value, the less trustworthy the prediciton. For highly non-normal X properties (e.g., fingerprints), the MD p-value is wildly inaccurate.

Structural Similar Compounds

Name	1,3,6-Naphthalenetrisulfonic acid, 7-amino-	2,2'-Benzidine disulfonic acid	2,7-Anthracenedisulfonic acid, 9,10-dihydro-4,5-diamino-9,10-dioxo- 1-hydroxy-, disodium salt
Structure	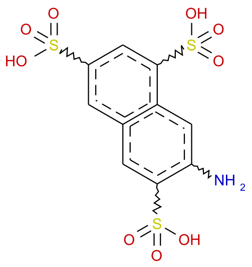	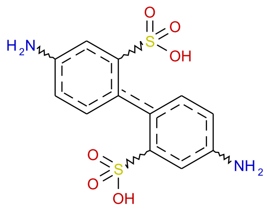	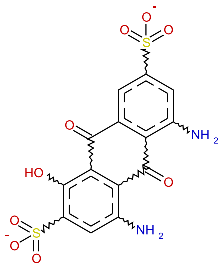
Actual Endpoint	Irritant	Non-Irritant	Irritant
Predicted Endpoint	Non-Irritant	Non-Irritant	Non-Irritant
Distance	0.755	0.896	1.025
Reference	85JCAE "Prehled Prumyslove Toxikologie; Organicke Latky," Marhold, J., Prague , Czechoslovakia, Avicenum, 1986 Volume(issue)/page/year: -,1058,1986	28ZPAK -,191,72	28ZPAK "Sbornik Vysledku Toxixologickeho Vysetreni Latek A Pripravku," Marhol d, J.V., Institut Pro Vychovu Vedoucicn Pracovniku Chemickeho Prumyclu Praha, Cz echoslovakia, 1972 Volume(issue)/page/year: -,239,1

Model Applicability

Unknown features are fingerprint features in the query molecule, but not found or appearing too infrequently in the training set.

1. All properties and OPS components are within expected ranges.
2. Unknown FCFP_2 feature: 472180098: [*]OP(=O)(O)O
3. Unknown FCFP_2 feature: -332197802: [*][c]1:[*]:[*]:[c](:[*]):n:1:n:[*]

Feature Contribution

Top features for positive contribution				
**Fingerprint**	**Bit/Smiles**	**Feature Structure**	**Score**	**Irritant in training set**

FCFP_12	654335567	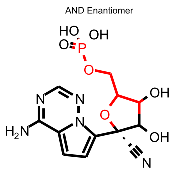 [*]C1[*][*]OC1COP(=[*])([*])[*]	0.0856	29 out of 29
FCFP_12	-1539132615	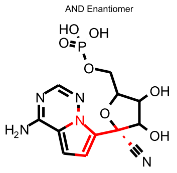 [*]C([*])([*])[c]1:[cH]:[*]:[*]:n:1:[*]	0.0795	9 out of 9
FCFP_12	-1280036918	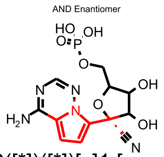 [*]C([*])([*])[c]1:[cH]:[cH]:[c](:[*]):n:1:[*]	0.0772	7 out of 7

Top Features for negative contribution

Fingerprint	Bit/Smiles	Feature Structure	Score	Irritant in training set
FCFP_12	1069584379	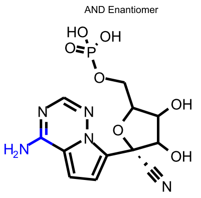 [*]:[c](:[*])N	-0.439	38 out of 65
FCFP_12	1618154665	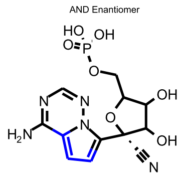 [*][c](:[*]):[cH]:[c]([*]):[*]	-0.0845	412 out of 490

FCFP_12	16	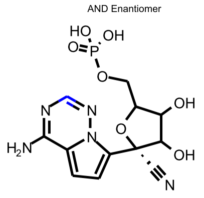 [*][c](:[*]):[*]	-0.0843	423 out of 503

new dicoumarin.cdx

TOPKAT_Carcinogenic_Potency_TD50_Mouse

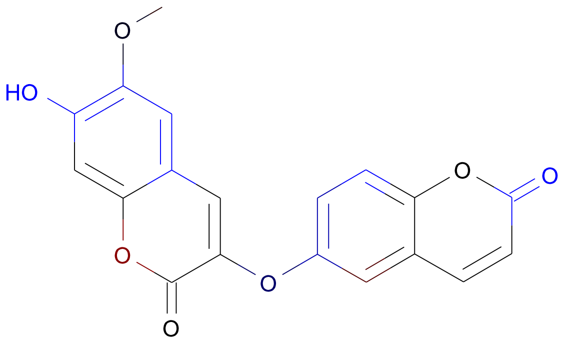

$C_{19}H_{12}O_7$

Molecular Weight: 352.29437

ALogP: 2.975

Rotatable Bonds: 3

Acceptors: 7

Donors: 1

Model Prediction

Prediction: 101

Unit: mg/kg_body_weight/day

Mahalanobis Distance: 10.1

Mahalanobis Distance p-value: 0.0278

Mahalanobis Distance: The Mahalanobis distance (MD) is a generalization of the Euclidean distance that accounts for correlations among the X properties. It is calculated as the distance to the center of the training data. The larger the MD, the less trustworthy the prediction.

Mahalanobis Distance p-value: The p-value gives the fraction of training data with an MD greater than or equal to the one for the given sample, assuming normally distributed data. The smaller the p-value, the less trustworthy the prediciton. For highly non-normal X properties (e.g., fingerprints), the MD p-value is wildly inaccurate.

Structural Similar Compounds

Name	Sterigmatocystin s	C.I. pigment red 3	Griseofulvin s
Structure	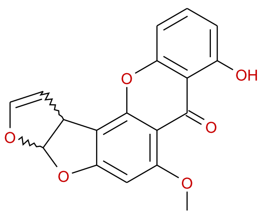	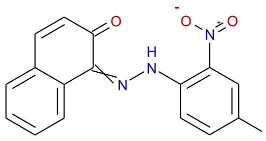	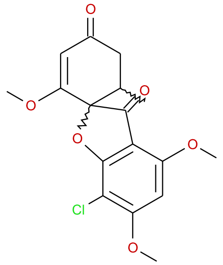
Actual Endpoint (-log C)	5.55284	0.937339	4.40761
Predicted Endpoint (-log C)	3.6442	3.17837	3.87261
Distance	0.612	0.656	0.703
Reference	CPDB	CPDB	CPDB

Model Applicability

Unknown features are fingerprint features in the query molecule, but not found or appearing too infrequently in the training set.

1. All properties and OPS components are within expected ranges.

Feature Contribution

Top features for positive contribution

Fingerprint	Bit/Smiles	Feature Structure	Score
ECFP_6	683445015	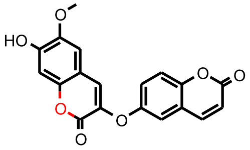 [*]O[*]	0.136

ECFP_6	-176455838	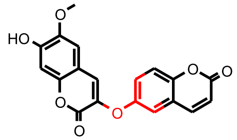 [*]O[c](:[cH]:[*]):[cH]:[*]	0.0818
ECFP_6	734603939	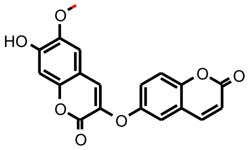 [*]C	0.0424

Top Features for negative contribution

Fingerprint	Bit/Smiles	Feature Structure	Score
ECFP_6	2106656448	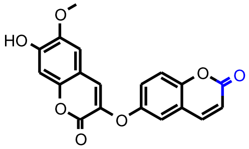 [*]C(=O)[*]	-0.275
ECFP_6	2019062761	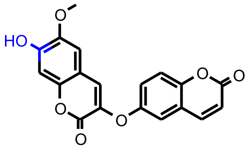 [*]:[c](:[*])O	-0.258
ECFP_6	1996767644	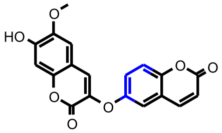 [*][c](:[*]):[cH]:[cH]:[*]	-0.251

remdesivir

TOPKAT_Carcinogenic_Potency_TD50_Mouse

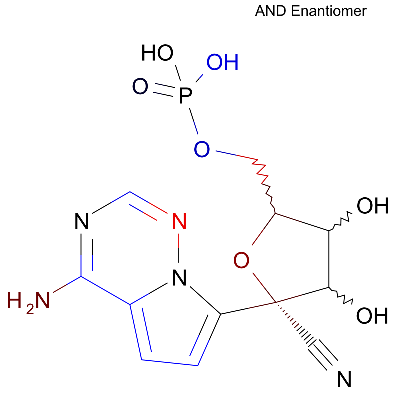

$C_{12}H_{14}N_5O_7P$

Molecular Weight: 371.24262

ALogP: -1.502

Rotatable Bonds: 4

Acceptors: 11

Donors: 5

Model Prediction

Prediction: 9.25

Unit: mg/kg_body_weight/day

Mahalanobis Distance: 14

Mahalanobis Distance p-value: 2.59e-010

Mahalanobis Distance: The Mahalanobis distance (MD) is a generalization of the Euclidean distance that accounts for correlations among the X properties. It is calculated as the distance to the center of the training data. The larger the MD, the less trustworthy the prediction.
Mahalanobis Distance p-value: The p-value gives the fraction of training data with an MD greater than or equal to the one for the given sample, assuming normally distributed data. The smaller the p-value, the less trustworthy the prediciton. For highly non-normal X properties (e.g., fingerprints), the MD p-value is wildly inaccurate.

Structural Similar Compounds

Name	377	(N-6)-(Methylnitroso)adenosine	338
Structure	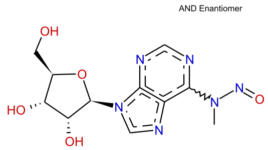	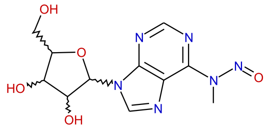	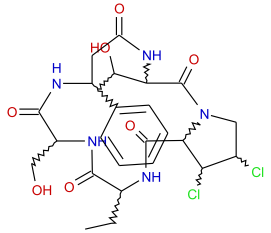
Actual Endpoint (-log C)	4.22928	4.22928	4.39533
Predicted Endpoint (-log C)	5.36013	5.36013	4.31268
Distance	0.852	0.852	0.919
Reference	CPDB	CPDB	CPDB

Model Applicability

Unknown features are fingerprint features in the query molecule, but not found or appearing too infrequently in the training set.

1. All properties and OPS components are within expected ranges.
2. Unknown ECFP_2 feature: 1126642748: [*]OP(=O)(O)O
3. Unknown ECFP_2 feature: 2024329577: [*]P(=[*])([*])O
4. Unknown ECFP_2 feature: -194719409: [*]C1[*][*]C([*])([*])O1
5. Unknown ECFP_2 feature: 1258791451: [*]C1[*][*]O[C@]1(C#[*])[c](:[*]):[*]
6. Unknown ECFP_2 feature: -264833661: [*]C([*])([*])C#N
7. Unknown ECFP_2 feature: -1507082173: [*][c]1:[*]:[*]:[c](:[*]):n:1:n:[*]
8. Unknown ECFP_2 feature: -676555381: [*]:[cH]:n:n(:[*]):[*]
9. Unknown ECFP_2 feature: -66263742: [*]C([*])([*])[c]1:[cH]:[*]:[*]:n:1:[*]

Feature Contribution

Top features for positive contribution			
**Fingerprint**	**Bit/Smiles**	**Feature Structure**	**Score**

ECFP_6	655739385	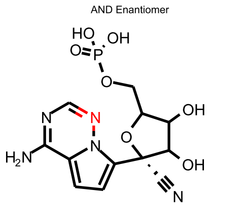 [*]:n:[*]	0.229
ECFP_6	1572579716	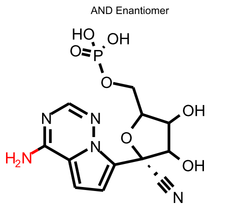 [*]N	0.225
ECFP_6	1559650422	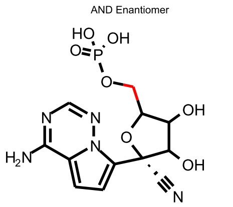 [*]C[*]	0.203

Top Features for negative contribution

Fingerprint	Bit/Smiles	Feature Structure	Score
ECFP_6	1996767644	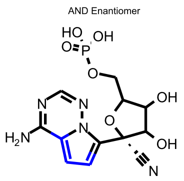 [*][c](:[*]):[cH]:[cH]:[*]	-0.251
ECFP_6	642810091	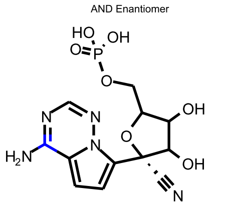 [*][c](:[*]):[*]	-0.247

ECFP_6	-182236392	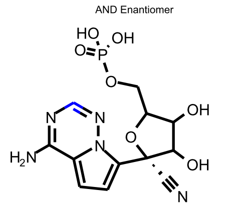<br>[*]:[cH]:[*]	-0.232

new dicoumarin.cdx

TOPKAT_Carcinogenic_Potency_TD50_Rat

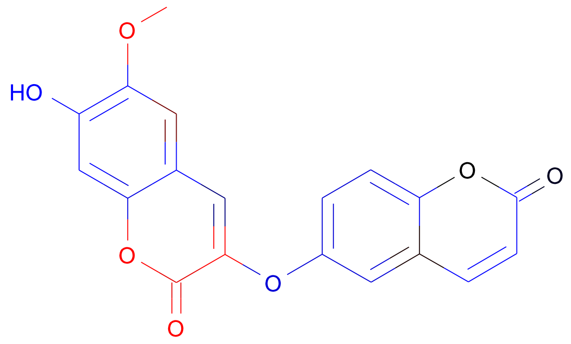

$C_{19}H_{12}O_7$
Molecular Weight: 352.29437
ALogP: 2.975
Rotatable Bonds: 3
Acceptors: 7
Donors: 1

Model Prediction

Prediction: 7.88
Unit: mg/kg_body_weight/day
Mahalanobis Distance: 14.4
Mahalanobis Distance p-value: 2.26e-009

Mahalanobis Distance: The Mahalanobis distance (MD) is a generalization of the Euclidean distance that accounts for correlations among the X properties. It is calculated as the distance to the center of the training data. The larger the MD, the less trustworthy the prediction.
Mahalanobis Distance p-value: The p-value gives the fraction of training data with an MD greater than or equal to the one for the given sample, assuming normally distributed data. The smaller the p-value, the less trustworthy the prediciton. For highly non-normal X properties (e.g., fingerprints), the MD p-value is wildly inaccurate.

Structural Similar Compounds

Name	5,6-Dimethoxysterigmatocystin	Sterigmatocystin s	C.I. pigment red 3
Structure	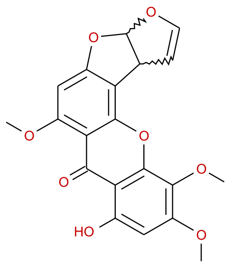	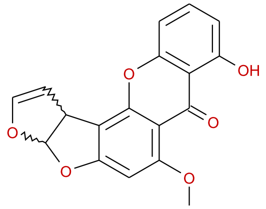	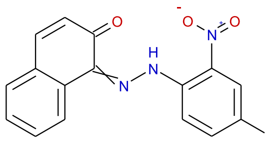
Actual Endpoint (-log C)	6.02361	6.32908	2.41938
Predicted Endpoint (-log C)	4.98771	4.10741	4.26375
Distance	0.555	0.587	0.632
Reference	CPDB	CPDB	CPDB

Model Applicability

Unknown features are fingerprint features in the query molecule, but not found or appearing too infrequently in the training set.

1. All properties and OPS components are within expected ranges.

Feature Contribution

Top features for positive contribution			
Fingerprint	Bit/Smiles	Feature Structure	Score
FCFP_6	136627117	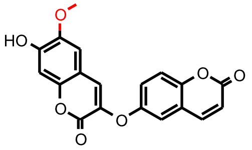 [*]OC	0.69

FCFP_6	565998553	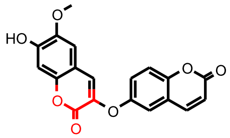<br>[*]OC(=O)C(=[*])[*]	0.357
FCFP_6	1	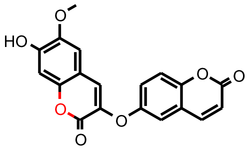<br>[*]O[*]	0.234
**Top Features for negative contribution**			
**Fingerprint**	**Bit/Smiles**	**Feature Structure**	**Score**
FCFP_6	451847724	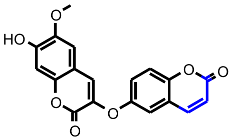<br>[*]\C=C/C(=[*])[*]	-0.436
FCFP_6	7	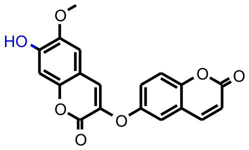<br>[*]O	-0.372
FCFP_6	16	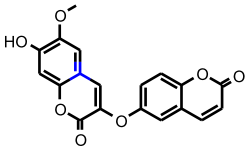<br>[*][c](:[*]):[*]	-0.354

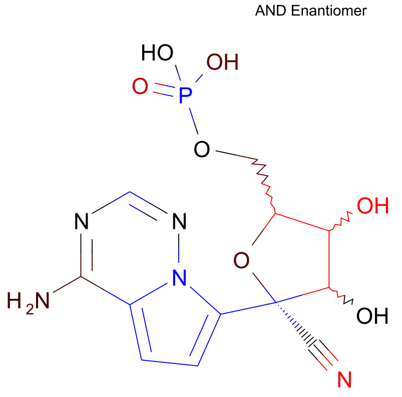

$C_{12}H_{14}N_5O_7P$
Molecular Weight: 371.24262
ALogP: -1.502
Rotatable Bonds: 4
Acceptors: 11
Donors: 5

Model Prediction

Prediction: 1.01
Unit: mg/kg_body_weight/day
Mahalanobis Distance: 16.2
Mahalanobis Distance p-value: 4.38e-015

Mahalanobis Distance: The Mahalanobis distance (MD) is a generalization of the Euclidean distance that accounts for correlations among the X properties. It is calculated as the distance to the center of the training data. The larger the MD, the less trustworthy the prediction.
Mahalanobis Distance p-value: The p-value gives the fraction of training data with an MD greater than or equal to the one for the given sample, assuming normally distributed data. The smaller the p-value, the less trustworthy the prediciton. For highly non-normal X properties (e.g., fingerprints), the MD p-value is wildly inaccurate.

Structural Similar Compounds

Name	b-Thioguanine deoxyriboside	Hexamethylmelamine	604
Structure	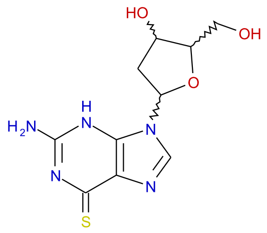	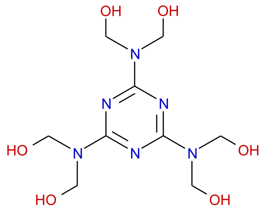	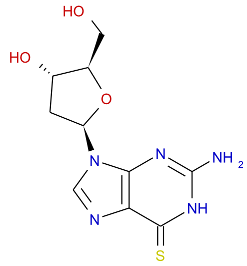
Actual Endpoint (-log C)	5.13004	4.47751	5.13004
Predicted Endpoint (-log C)	4.82552	3.76275	4.96687
Distance	0.805	0.832	0.835
Reference	CPDB	CPDB	CPDB

Model Applicability

Unknown features are fingerprint features in the query molecule, but not found or appearing too infrequently in the training set.

1. All properties and OPS components are within expected ranges.
2. Unknown FCFP_2 feature: 472180098: [*]OP(=O)(O)O
3. Unknown FCFP_2 feature: -836603894: [*]C1[*][*]O[C@]1(C#[*])[c](:[*]):[*]

Feature Contribution

Top features for positive contribution			
**Fingerprint**	**Bit/Smiles**	**Feature Structure**	**Score**
FCFP_6	-1043250487	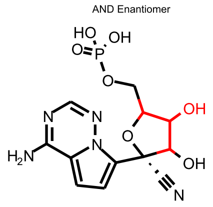 [*]C1[*][*]C([*])C1O	1.15

FCFP_6	9	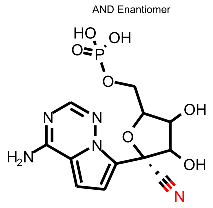 [*]#N	0.385
FCFP_6	1	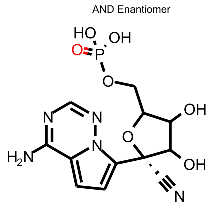 [*]O[*]	0.234
**Top Features for negative contribution**			
**Fingerprint**	**Bit/Smiles**	**Feature Structure**	**Score**
FCFP_6	-1280036918	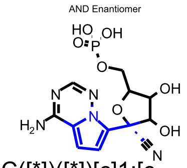 [*]C([*])([*])[c]1:[cH]:[cH]:[c](:[*]):n:1:[*]	-0.363
FCFP_6	16	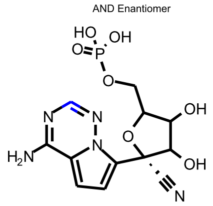 [*][c](:[*]):[*]	-0.354
FCFP_6	17	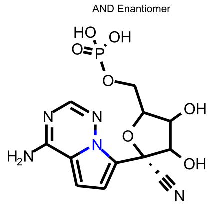 [*]:n(:[*]):[*]	-0.149

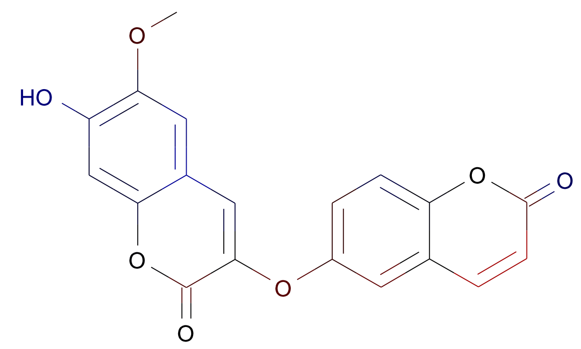

$C_{19}H_{12}O_7$

Molecular Weight: 352.29437

ALogP: 2.975

Rotatable Bonds: 3

Acceptors: 7

Donors: 1

Structural Similar Compounds

Name	ROTENONE	3;3'-DIMETHOXYBENZIDINE-4;4'-DIISOCYANATE	SODIUM ACIFLUORFEN
Structure	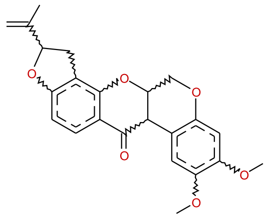	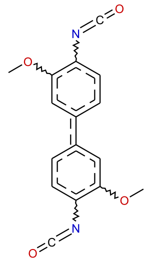	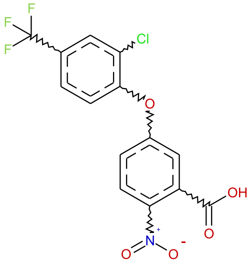
Actual Endpoint (-log C)	5.0219	2.2956	4.16036
Predicted Endpoint (-log C)	4.24871	3.47444	4.65915
Distance	0.590	0.601	0.623
Reference	NTP REPORT # 320	NTP 128 31	EPA COVER SHEET 0192;891101;(1)

Model Prediction

Prediction: 0.0221

Unit: g/kg_body_weight

Mahalanobis Distance: 28.1

Mahalanobis Distance p-value: 3.16e-021

Mahalanobis Distance: The Mahalanobis distance (MD) is a generalization of the Euclidean distance that accounts for correlations among the X properties. It is calculated as the distance to the center of the training data. The larger the MD, the less trustworthy the prediction.
Mahalanobis Distance p-value: The p-value gives the fraction of training data with an MD greater than or equal to the one for the given sample, assuming normally distributed data. The smaller the p-value, the less trustworthy the prediciton. For highly non-normal X properties (e.g., fingerprints), the MD p-value is wildly inaccurate.

Model Applicability

Unknown features are fingerprint features in the query molecule, but not found or appearing too infrequently in the training set.

1. All properties and OPS components are within expected ranges.
2. Unknown ECFP_6 feature: 1336666212: [*][c](:[*]):[c](C=[*]):[cH]:[*]
3. Unknown ECFP_6 feature: -570915357: [*]O[c](:[cH]:[*]):[c]([*]):[*]
4. Unknown ECFP_6 feature: 464808839: [*]C(=C[c](:[*]):[*])[*]
5. Unknown ECFP_6 feature: -741588105: [*]O\C(=C\[*])\C(=[*])[*]
6. Unknown ECFP_6 feature: -560785749: [*]C(=[*])O[c](:[*]):[*]
7. Unknown ECFP_6 feature: 1573945311: [*]OC(=O)C(=[*])[*]
8. Unknown ECFP_6 feature: 1305253718: [*]C(=[*])O[c](:[*]):[*]
9. Unknown ECFP_6 feature: 1745066357: [*]\C=C/[c](:[*]):[*]
10. Unknown ECFP_6 feature: -1885846789: [*]OC(=O)C=[*]
11. Unknown ECFP_6 feature: 1307307440: [*]:[c](:[*])OC
12. Unknown ECFP_6 feature: 2019062761: [*]:[c](:[*])O

Feature Contribution

Top features for positive contribution			
Fingerprint	Bit/Smiles	Feature Structure	Score

FCFP_6	451847724	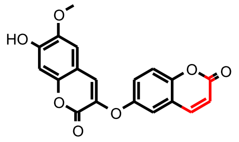 [*]\C=C/C(=[*])[*]	0.16
ECFP_6	-176455838	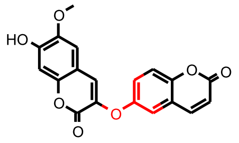 [*]O[c](:[cH]:[*]):[cH]:[*]	0.106
ECFP_6	683445015	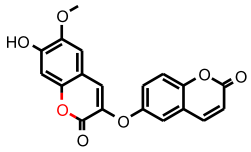 [*]O[*]	0.0734

Top Features for negative contribution

Fingerprint	Bit/Smiles	Feature Structure	Score
ECFP_6	2106656448	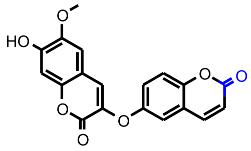 [*]C(=O)[*]	-0.11
FCFP_6	1	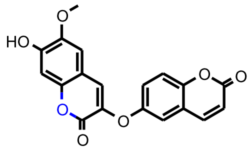 [*]O[*]	-0.102

FCFP_6	203677720	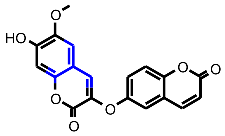<br>[*][c](:[*]):[c](C=[*<br>]):[cH]:[*]	-0.0713

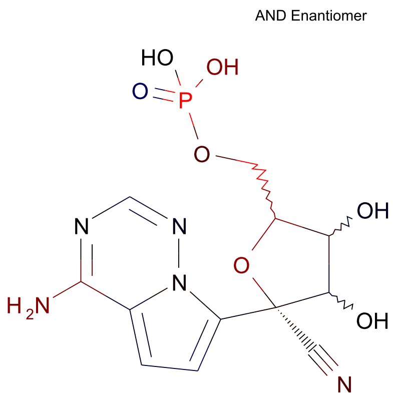

$C_{12}H_{14}N_5O_7P$
Molecular Weight: 371.24262
ALogP: -1.502
Rotatable Bonds: 4
Acceptors: 11
Donors: 5

Model Prediction

Prediction: 0.00379
Unit: g/kg_body_weight
Mahalanobis Distance: 47.7
Mahalanobis Distance p-value: 2.93e-054

Mahalanobis Distance: The Mahalanobis distance (MD) is a generalization of the Euclidean distance that accounts for correlations among the X properties. It is calculated as the distance to the center of the training data. The larger the MD, the less trustworthy the prediction.
Mahalanobis Distance p-value: The p-value gives the fraction of training data with an MD greater than or equal to the one for the given sample, assuming normally distributed data. The smaller the p-value, the less trustworthy the prediciton. For highly non-normal X properties (e.g., fingerprints), the MD p-value is wildly inaccurate.

Structural Similar Compounds

Name	TETRACYCLINE .HCL	4;4'-DIAMINO-2;2'-STILBENEDIS	OXYTETRACYCLINE .HCL
Structure	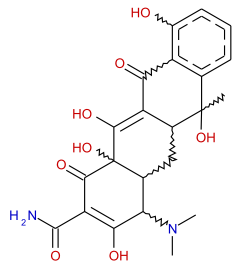	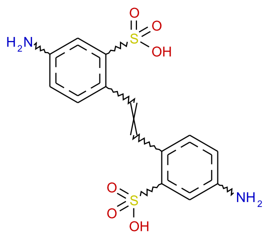	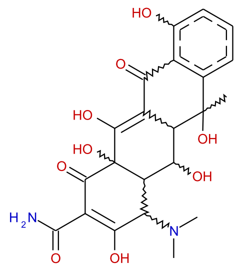
Actual Endpoint (-log C)	2.85193	2.47175	2.56626
Predicted Endpoint (-log C)	3.94748	3.53715	3.75581
Distance	0.746	0.746	0.802
Reference	NTP REPORT # 344	NTP 412 82	NTP REPORT # 315

Model Applicability

Unknown features are fingerprint features in the query molecule, but not found or appearing too infrequently in the training set.

1. All properties and OPS components are within expected ranges.
2. Unknown FCFP_2 feature: 472180098: [*]OP(=O)(O)O
3. Unknown FCFP_2 feature: -332197802: [*][c]1:[*]:[*]:[c](:[*]):n:1:n:[*]
4. Unknown ECFP_6 feature: -1114776580: [*]C#[*]
5. Unknown ECFP_6 feature: -1101847286: [*]#N
6. Unknown ECFP_6 feature: 672362763: [*]:n(:[*]):[*]
7. Unknown ECFP_6 feature: 1126642748: [*]OP(=O)(O)O
8. Unknown ECFP_6 feature: 2100964382: [*]P(=O)([*])[*]
9. Unknown ECFP_6 feature: 2024329577: [*]P(=[*])([*])O
10. Unknown ECFP_6 feature: -1250439909: [*]COP(=[*])([*])[*]
11. Unknown ECFP_6 feature: -1687549011: [*]OCC([*])[*]
12. Unknown ECFP_6 feature: -194719409: [*]C1[*][*]C([*])([*])O1
13. Unknown ECFP_6 feature: -553149446: [*]CC1O[*][*]C1[*]
14. Unknown ECFP_6 feature: 305695353: [*]C1[*][*]C([*])C1O
15. Unknown ECFP_6 feature: -521596699: [*]C1[*][*]C([*])([*])C1O
16. Unknown ECFP_6 feature: 1258791451: [*]C1[*][*]O[C@]1(C#[*])[c](:[*]):[*]
17. Unknown ECFP_6 feature: 2024749573: [*]C([*])O
18. Unknown ECFP_6 feature: -264833661: [*]C([*])([*])C#N
19. Unknown ECFP_6 feature: 1412053881: [*]C#N

20. Unknown ECFP_6 feature: -1507082173: [*][c]1:[*]:[*]:[c](:[*]):n:1:n:[*]
21. Unknown ECFP_6 feature: -676555381: [*]:[cH]:n:n(:[*]):[*]
22. Unknown ECFP_6 feature: -710237522: [*]:n:[cH]:n:[*]
23. Unknown ECFP_6 feature: -677309799: [*][c](:[*]):n:[cH]:[*]
24. Unknown ECFP_6 feature: -1734834311: [*]:n:[c](N):[c](:[*]):[*]
25. Unknown ECFP_6 feature: 1334415134: [*][c](:[*]):[c]1:[cH]:[*]:[*]:n:1:[*]
26. Unknown ECFP_6 feature: -66263742: [*]C([*])([*])[c]1:[cH]:[*]:[*]:n:1:[*]
27. Unknown ECFP_6 feature: -938530932: [*]:[c](:[*])N

Feature Contribution

Top features for positive contribution

Fingerprint	Bit/Smiles	Feature Structure	Score
ECFP_6	-167460056	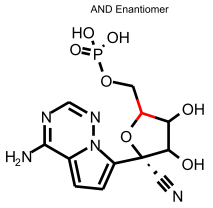 [*]C([*])[*]	0.136
FCFP_6	-1143715940	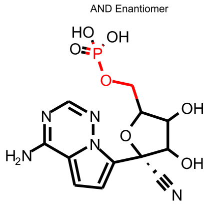 [*]COP(=[*])([*])[*]	0.13
ECFP_6	1559650422	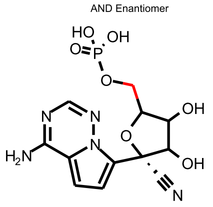 [*]C[*]	0.129

Top Features for negative contribution

Fingerprint	Bit/Smiles	Feature Structure	Score

FCFP_6	1	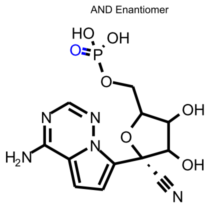[*]O[*]	-0.102
ECFP_6	1996767644	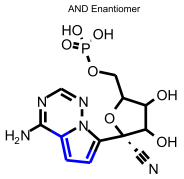[*][c](:[*]):[cH]:[cH]:[*]	-0.0497
FCFP_6	16	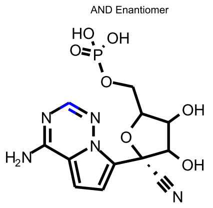[*][c](:[*]):[*]	-0.0462

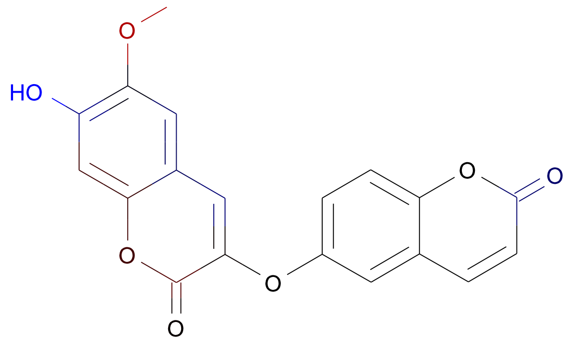

$C_{19}H_{12}O_7$
Molecular Weight: 352.29437
ALogP: 2.975
Rotatable Bonds: 3
Acceptors: 7
Donors: 1

Structural Similar Compounds

Name	3,3'-DIMETHOXYBENZIDINE-4,4'-DIISOCYANATE	ROTENONE	C.I.PIGMENT RED 3
Structure	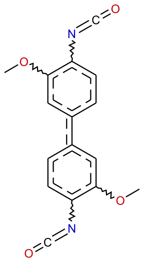	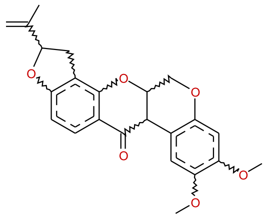	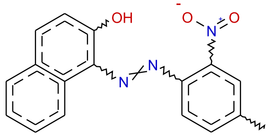
Actual Endpoint (-log C)	2.17504	5.06769	2.65635
Predicted Endpoint (-log C)	3.78717	4.11907	2.97957
Distance	0.532	0.592	0.594
Reference	NCI/NTP TR-128	NCI/NTP TR-320	NCI/NTP TR-407

Model Prediction

Prediction: 0.117
Unit: g/kg_body_weight
Mahalanobis Distance: 7.65
Mahalanobis Distance p-value: 0.0562

Mahalanobis Distance: The Mahalanobis distance (MD) is a generalization of the Euclidean distance that accounts for correlations among the X properties. It is calculated as the distance to the center of the training data. The larger the MD, the less trustworthy the prediction.
Mahalanobis Distance p-value: The p-value gives the fraction of training data with an MD greater than or equal to the one for the given sample, assuming normally distributed data. The smaller the p-value, the less trustworthy the prediciton. For highly non-normal X properties (e.g., fingerprints), the MD p-value is wildly inaccurate.

Model Applicability

Unknown features are fingerprint features in the query molecule, but not found or appearing too infrequently in the training set.

1. All properties and OPS components are within expected ranges.

Feature Contribution

Top features for positive contribution

Fingerprint	Bit/Smiles	Feature Structure	Score
FCFP_2	136627117	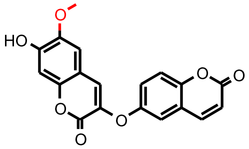 [*]OC	0.173

FCFP_2	1036089772	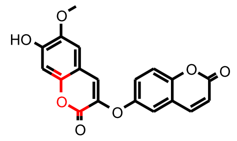 [*]C(=[*])O[c](:[*]):[*]	0.0749
FCFP_2	332760439	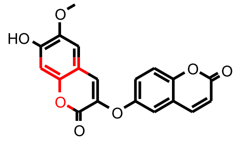 [*]O[c](:[cH]:[*]):[c]([*]):[*]	0.0611
**Top Features for negative contribution**			
**Fingerprint**	**Bit/Smiles**	**Feature Structure**	**Score**
FCFP_2	7	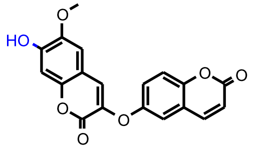 [*]O	-0.214
FCFP_2	-549108873	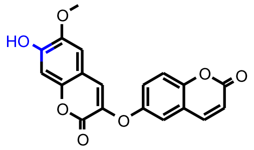 [*]:[c](:[*])O	-0.127
FCFP_2	1872154524	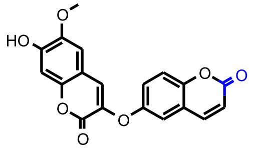 [*]C(=O)[*]	-0.105

remdesivir

TOPKAT_Rat_Maximum_Tolerated_Dose_Feed

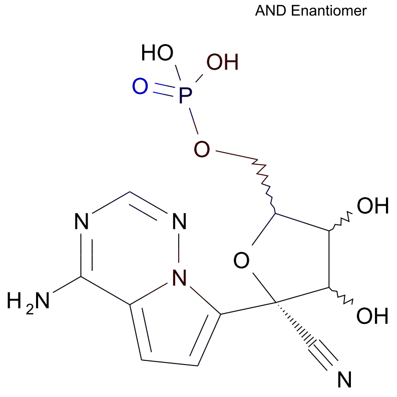

$C_{12}H_{14}N_5O_7P$

Molecular Weight: 371.24262

ALogP: -1.502

Rotatable Bonds: 4

Acceptors: 11

Donors: 5

Model Prediction

Prediction: 0.235

Unit: g/kg_body_weight

Mahalanobis Distance: 9.52

Mahalanobis Distance p-value: 0.000247

Mahalanobis Distance: The Mahalanobis distance (MD) is a generalization of the Euclidean distance that accounts for correlations among the X properties. It is calculated as the distance to the center of the training data. The larger the MD, the less trustworthy the prediction.
Mahalanobis Distance p-value: The p-value gives the fraction of training data with an MD greater than or equal to the one for the given sample, assuming normally distributed data. The smaller the p-value, the less trustworthy the prediciton. For highly non-normal X properties (e.g., fingerprints), the MD p-value is wildly inaccurate.

Structural Similar Compounds

Name	4,4'-DIAMINO-2,2'-STILBENEDISULFONIC ACID.2NaSALT	OXYTETRACYCLINE	50%1,4,5,8-TETRAAMINOANTHRAQUIONONE + DERIVATIVES
Structure	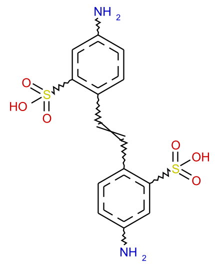	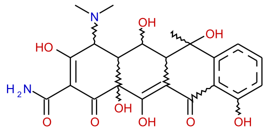	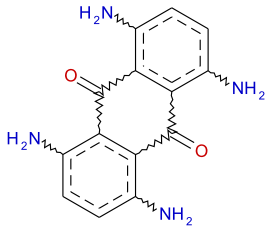
Actual Endpoint (-log C)	2.50759	2.36214	3.0764
Predicted Endpoint (-log C)	3.26068	2.77834	3.08142
Distance	0.743	0.818	0.989
Reference	NCI/NTP TR-412	NCI/NTP TR-315	NCI/NTP TR-299

Model Applicability

Unknown features are fingerprint features in the query molecule, but not found or appearing too infrequently in the training set.

1. Molecular_PolarSurfaceArea out of range. Value: 206.26. Training min, max, mean, SD: 0, 201.84, 63.052, 40.7.
2. Unknown FCFP_2 feature: 472180098: [*]OP(=O)(O)O
3. Unknown FCFP_2 feature: -836603894: [*]C1[*][*]O[C@]1(C#[*])[c](:[*]):[*]
4. Unknown FCFP_2 feature: -1277879912: [*]C([*])([*])C#N
5. Unknown FCFP_2 feature: -332197802: [*][c]1:[*]:[*]:[c](:[*]):n:1:n:[*]
6. Unknown FCFP_2 feature: -124685461: [*]:n:[cH]:n:[*]

Feature Contribution

Top features for positive contribution

Fingerprint	Bit/Smiles	Feature Structure	Score

FCFP_2	-1143715940	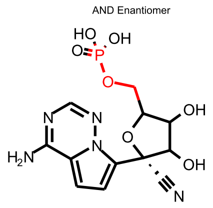<br>[*]COP(=[*])([*])[*]	0.095
FCFP_2	3	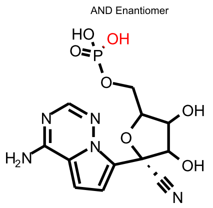<br>[*]O	0.0737
FCFP_2	17	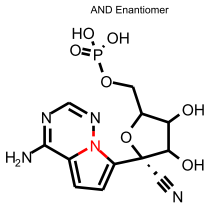<br>[*]:n(:[*]):[*]	0.0441
**Top Features for negative contribution**			
**Fingerprint**	**Bit/Smiles**	**Feature Structure**	**Score**
FCFP_2	1872154524	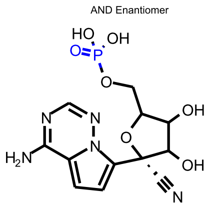<br>[*]C(=O)[*]	-0.105
FCFP_2	1	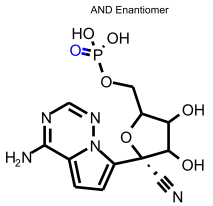<br>[*]O[*]	-0.0796

FCFP_2	16	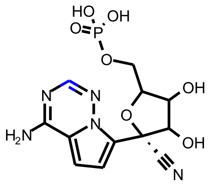 [*][c](:[*]):[*]	-0.0512

new dicoumarin.cdx

TOPKAT_Rat_Maximum_Tolerated_Dose_Gavage

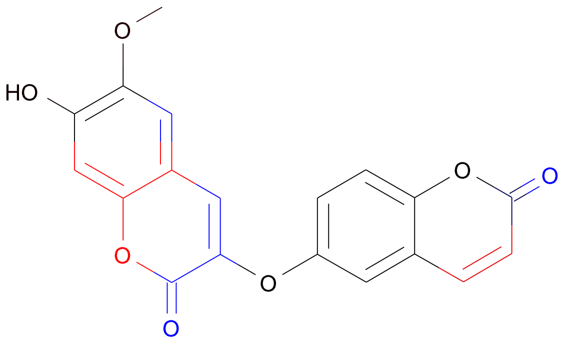

$C_{19}H_{12}O_7$
Molecular Weight: 352.29437
ALogP: 2.975
Rotatable Bonds: 3
Acceptors: 7
Donors: 1

Model Prediction

Prediction: 0.000177
Unit: g/kg_body_weight
Mahalanobis Distance: 12
Mahalanobis Distance p-value: 8.53e-009

Mahalanobis Distance: The Mahalanobis distance (MD) is a generalization of the Euclidean distance that accounts for correlations among the X properties. It is calculated as the distance to the center of the training data. The larger the MD, the less trustworthy the prediction.
Mahalanobis Distance p-value: The p-value gives the fraction of training data with an MD greater than or equal to the one for the given sample, assuming normally distributed data. The smaller the p-value, the less trustworthy the prediciton. For highly non-normal X properties (e.g., fingerprints), the MD p-value is wildly inaccurate.

Structural Similar Compounds

Name	PENICILLIN VK	PROBENECID	8-METHOXYPSORALEN
Structure	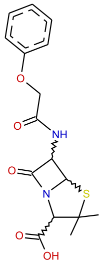	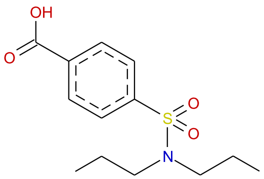	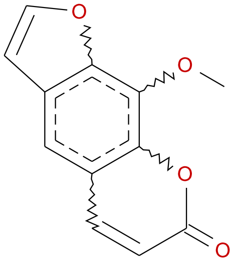
Actual Endpoint (-log C)	2.54455	2.85333	3.45978
Predicted Endpoint (-log C)	3.9702	2.4258	4.14745
Distance	0.835	0.867	0.868
Reference	NCI/NTP TR-336	NCI/NTP TR-395	NCI/NTP TR-359

Model Applicability

Unknown features are fingerprint features in the query molecule, but not found or appearing too infrequently in the training set.

1. Num_H_Acceptors out of range. Value: 7. Training min, max, mean, SD: 0, 6, 1.6146, 1.644.
2. OPS PC9 out of range. Value: 4.6388. Training min, max, SD, explained variance: -2.7086, 2.9267, 1.019, 0.0321.
3. Unknown FCFP_2 feature: 436915834: [*]O\C(=C\[*])\C(=[*])[*]

Feature Contribution

Top features for positive contribution

Fingerprint	Bit/Smiles	Feature Structure	Score
FCFP_2	332760439	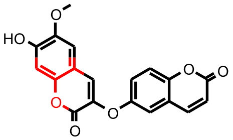 [*]O[c](:[cH]:[*]):[c]([*]):[*]	0.672

FCFP_2	1	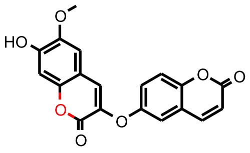 [*]O[*]	0.511
FCFP_2	451847724	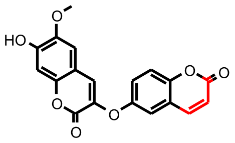 [*]\C=C/C(=[*])[*]	0.225
**Top Features for negative contribution**			
**Fingerprint**	**Bit/Smiles**	**Feature Structure**	**Score**
FCFP_2	203677720	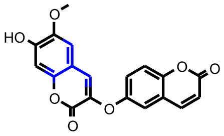 [*][c](:[*]):[c](C=[*<br>]):[cH]:[*]	-0.406
FCFP_2	565998553	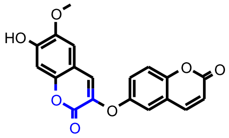 [*]OC(=O)C(=[*])[*]	-0.348
FCFP_2	1872154524	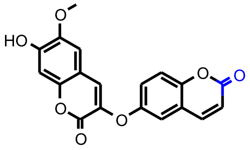 [*]C(=O)[*]	-0.307

remdesivir

TOPKAT_Rat_Maximum_Tolerated_Dose_Gavage

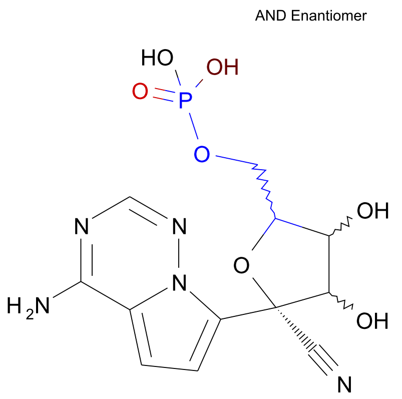

$C_{12}H_{14}N_5O_7P$

Molecular Weight: 371.24262

ALogP: -1.502

Rotatable Bonds: 4

Acceptors: 11

Donors: 5

Model Prediction

Prediction: 0.000298

Unit: g/kg_body_weight

Mahalanobis Distance: 17.2

Mahalanobis Distance p-value: 5.05e-016

Mahalanobis Distance: The Mahalanobis distance (MD) is a generalization of the Euclidean distance that accounts for correlations among the X properties. It is calculated as the distance to the center of the training data. The larger the MD, the less trustworthy the prediction.
Mahalanobis Distance p-value: The p-value gives the fraction of training data with an MD greater than or equal to the one for the given sample, assuming normally distributed data. The smaller the p-value, the less trustworthy the prediciton. For highly non-normal X properties (e.g., fingerprints), the MD p-value is wildly inaccurate.

Structural Similar Compounds

Name	AMPICILLIN TRIHYDRATE	OCHRATOXIN	PENICILLIN VK
Structure	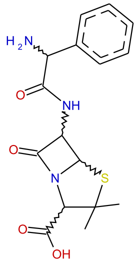	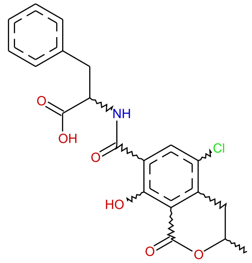	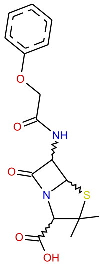
Actual Endpoint (-log C)	2.36724	6.28396	2.54455
Predicted Endpoint (-log C)	2.27651	5.12358	3.9702
Distance	1.255	1.482	1.498
Reference	NCI/NTP TR-318	NCI/NTP TR-358	NCI/NTP TR-336

Model Applicability

Unknown features are fingerprint features in the query molecule, but not found or appearing too infrequently in the training set.

1. Num_H_Donors out of range. Value: 5. Training min, max, mean, SD: 0, 3, 0.4375, 0.8311.
2. Num_H_Acceptors out of range. Value: 11. Training min, max, mean, SD: 0, 6, 1.6146, 1.644.
3. Molecular_PolarSASA out of range. Value: 321.97. Training min, max, mean, SD: 0, 223.97, 50.816, 55.15.
4. Molecular_PolarSurfaceArea out of range. Value: 206.26. Training min, max, mean, SD: 0, 138.03, 28.978, 32.1.
5. OPS PC1 out of range. Value: 9.0116. Training min, max, SD, explained variance: -4.0008, 7.9165, 2.861, 0.2531.
6. OPS PC5 out of range. Value: -4.1876. Training min, max, SD, explained variance: -3.4, 4.1587, 1.489, 0.0686.
7. OPS PC9 out of range. Value: -2.7276. Training min, max, SD, explained variance: -2.7086, 2.9267, 1.019, 0.0321.
8. Unknown FCFP_2 feature: 472180098: [*]OP(=O)(O)O
9. Unknown FCFP_2 feature: -836603894: [*]C1[*][*]O[C@]1(C#[*])[c](:[*]):[*]
10. Unknown FCFP_2 feature: -1277879912: [*]C([*])([*])C#N
11. Unknown FCFP_2 feature: -1362791977: [*]C#N
12. Unknown FCFP_2 feature: -332197802: [*][c]1:[*]:[*]:[c](:[*]):n:1:n:[*]
13. Unknown FCFP_2 feature: -124685461: [*]:n:[cH]:n:[*]

Feature Contribution

Top features for positive contribution

Fingerprint	Bit/Smiles	Feature Structure	Score
FCFP_2	1	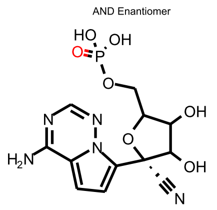 [*]O[*]	0.511
FCFP_2	3	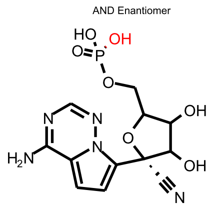 [*]O	0.104

Top Features for negative contribution

Fingerprint	Bit/Smiles	Feature Structure	Score
FCFP_2	1872154524	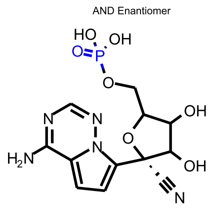 [*]C(=O)[*]	-0.307
FCFP_2	0	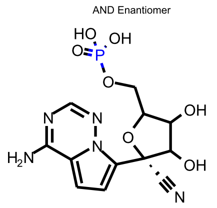 [*]C=[*]	-0.29

FCFP_2	-1272768868	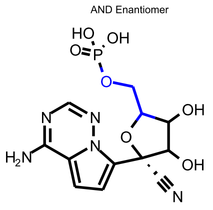 [*]OCC([*])[*]	-0.271

new dicoumarin.cdx

TOPKAT_Rat_Oral_LD50

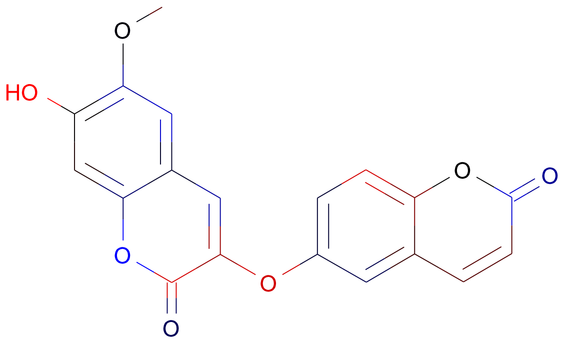

$C_{19}H_{12}O_7$
Molecular Weight: 352.29437
ALogP: 2.975
Rotatable Bonds: 3
Acceptors: 7
Donors: 1

Structural Similar Compounds

Name	STERIGMATOCYSTIN	DICOUMAROL	METHYL ORANGE; SODIUM SALT (Na STRIPPED)
Structure	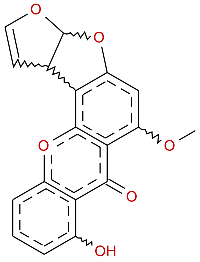	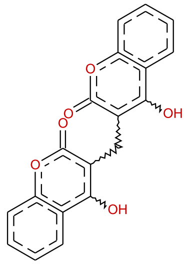	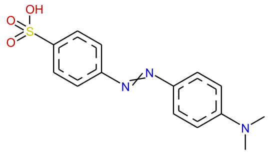
Actual Endpoint (-log C)	3.432	3.129	3.707
Predicted Endpoint (-log C)	3.69503	2.86156	2.64236
Distance	0.509	0.543	0.588
Reference	FCTXAV 7;135;69	SMWOAS 83;471;53	85JCAE -;1306;86

Model Prediction

Prediction: 0.288
Unit: g/kg_body_weight
Mahalanobis Distance: 16.5
Mahalanobis Distance p-value: 0.121

Mahalanobis Distance: The Mahalanobis distance (MD) is a generalization of the Euclidean distance that accounts for correlations among the X properties. It is calculated as the distance to the center of the training data. The larger the MD, the less trustworthy the prediction.
Mahalanobis Distance p-value: The p-value gives the fraction of training data with an MD greater than or equal to the one for the given sample, assuming normally distributed data. The smaller the p-value, the less trustworthy the prediciton. For highly non-normal X properties (e.g., fingerprints), the MD p-value is wildly inaccurate.

Model Applicability

Unknown features are fingerprint features in the query molecule, but not found or appearing too infrequently in the training set.

1. All properties and OPS components are within expected ranges.
2. Unknown FCFP_6 feature: 16: [*][c](:[*]):[*]
3. Unknown FCFP_6 feature: 1618154665: [*][c](:[*]):[cH]:[c]([*]):[*]
4. Unknown FCFP_6 feature: 74595001: [*][c](:[*]):[c](O):[cH]:[*]
5. Unknown FCFP_6 feature: 451371068: [*]C(=C[c](:[*]):[*])[*]
6. Unknown FCFP_6 feature: -549108873: [*]:[c](:[*])O

Feature Contribution

Top features for positive contribution			
Fingerprint	Bit/Smiles	Feature Structure	Score

ECFP_6	642810091	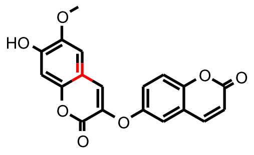 [*][c](:[*]):[*]	0.281
ECFP_6	-560785749	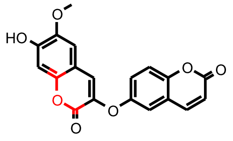 [*]C(=[*])O[c](:[*]):[*]	0.259
FCFP_6	436915834	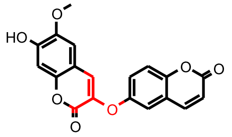 [*]O\C(=C\[*])\C(=[*])[*]	0.184

Top Features for negative contribution

Fingerprint	Bit/Smiles	Feature Structure	Score
ECFP_6	2106656448	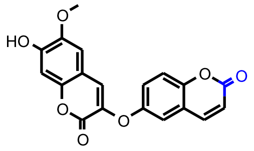 [*]C(=O)[*]	-0.352
ECFP_6	683445015	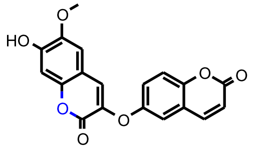 [*]O[*]	-0.266

ECFP_6	-176455838	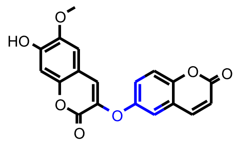<br>[*]O[c](:[cH]:[*]):[cH]:[*]	-0.257

remdesivir

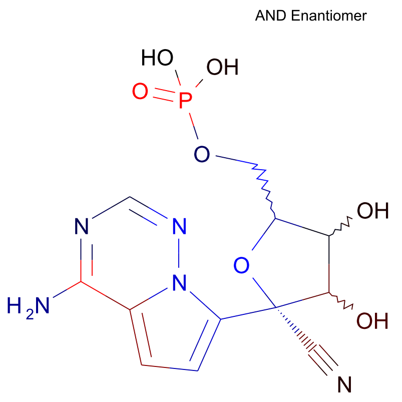

$C_{12}H_{14}N_5O_7P$
Molecular Weight: 371.24262
ALogP: -1.502
Rotatable Bonds: 4
Acceptors: 11
Donors: 5

Structural Similar Compounds

Name	5'-ADENYLIC ACID; POTASSIUM SALT (K STRIPPED)	INOSINATE; DISODIUM SALT (Na STRIPPED)	INOSINE-5'-PHOSPHORIC ACID
Structure	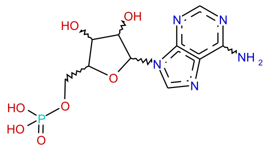	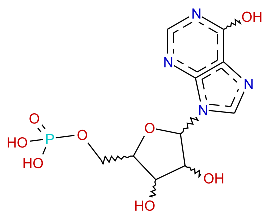	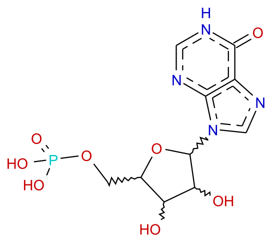
Actual Endpoint (-log C)	1.49	1.34	1.338
Predicted Endpoint (-log C)	2.45569	2.92201	1.35922
Distance	0.361	0.428	0.592
Reference	OYYAA2 4;689;70	AJINO* -;-;73	ARTODN 47;77;81

Model Prediction

Prediction: 0.309
Unit: g/kg_body_weight
Mahalanobis Distance: 29.4
Mahalanobis Distance p-value: 1.72e-059

Mahalanobis Distance: The Mahalanobis distance (MD) is a generalization of the Euclidean distance that accounts for correlations among the X properties. It is calculated as the distance to the center of the training data. The larger the MD, the less trustworthy the prediction.
Mahalanobis Distance p-value: The p-value gives the fraction of training data with an MD greater than or equal to the one for the given sample, assuming normally distributed data. The smaller the p-value, the less trustworthy the prediciton. For highly non-normal X properties (e.g., fingerprints), the MD p-value is wildly inaccurate.

Model Applicability

Unknown features are fingerprint features in the query molecule, but not found or appearing too infrequently in the training set.

1. OPS PC10 out of range. Value: 15.526. Training min, max, SD, explained variance: -6.0395, 14.892, 2.468, 0.0220.
2. Unknown ECFP_2 feature: 1258791451: [*]C1[*][*]O[C@]1(C#[*])[c](:[*]):[*]
3. Unknown ECFP_2 feature: -264833661: [*]C([*])([*])C#N
4. Unknown ECFP_2 feature: -66263742: [*]C([*])([*])[c]1:[cH]:[*]:[*]:n:1:[*]
5. Unknown FCFP_6 feature: 16: [*][c](:[*]):[*]
6. Unknown FCFP_6 feature: 472180098: [*]OP(=O)(O)O
7. Unknown FCFP_6 feature: -836603894: [*]C1[*][*]O[C@]1(C#[*])[c](:[*]):[*]
8. Unknown FCFP_6 feature: -332197802: [*][c]1:[*]:[*]:[c](:[*]):n:1:n:[*]
9. Unknown FCFP_6 feature: 4427049: [*]:[cH]:n:n(:[*]):[*]
10. Unknown FCFP_6 feature: -124685461: [*]:n:[cH]:n:[*]
11. Unknown FCFP_6 feature: 1747237384: [*][c](:[*]):n:[cH]:[*]
12. Unknown FCFP_6 feature: -1151884458: [*]:n:[c](N):[c](:[*]):[*]
13. Unknown FCFP_6 feature: 1618154665: [*][c](:[*]):[cH]:[c]([*]):[*]
14. Unknown FCFP_6 feature: 1069584379: [*]:[c](:[*])N

Feature Contribution

Top features for positive contribution

Fingerprint	Bit/Smiles	Feature Structure	Score
ECFP_6	642810091	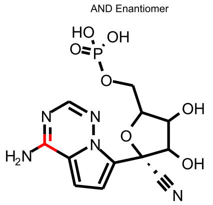 [*][c](:[*]):[*]	0.281
ECFP_6	-826638028	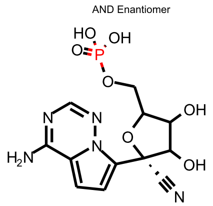 [*]P(=[*])([*])[*]	0.225
ECFP_6	2100964382	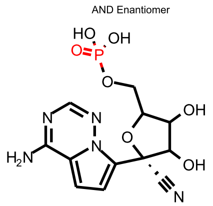 [*]P(=O)([*])[*]	0.166

Top Features for negative contribution

Fingerprint	Bit/Smiles	Feature Structure	Score
ECFP_6	683445015	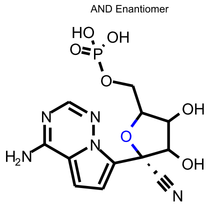 [*]O[*]	-0.266

ECFP_6	655739385	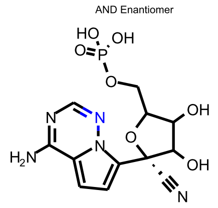 [*]:n:[*]	-0.239
FCFP_6	-1539132615	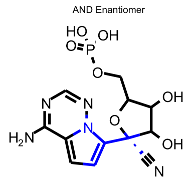 [*]C([*])([*])[c]1:[cH]:[*]:[*]:n:1:[*]	-0.2
